# Metabolic and Transcriptomic Profile Revealing the Differential Accumulating Mechanism in Different Parts of *Dendrobium nobile*

**DOI:** 10.3390/ijms25105356

**Published:** 2024-05-14

**Authors:** Ruoxi Zhao, Shou Yan, Yadong Hu, Dan Rao, Hongjie Li, Ze Chun, Shigang Zheng

**Affiliations:** 1 Chengdu Institute of Biology, Chinese Academy of Sciences, Chengdu 610041, China; zhaorx@cib.ac.cn (R.Z.);; 2 Hejiang Public Inspection and Testing Center, Sichuan Quality Supervision and Inspection Center for Se-rich and Zn-rich Products, Luzhou 646200, China; 3 College of Life Sciences, University of Chinese Academy of Sciences, Beijing 100041, China

**Keywords:** medicinal plant, chemical components, accumulation, biosynthesis, transporting, regulation

## Abstract

*Dendrobium nobile* is an important orchid plant that has been used as a traditional herb for many years. For the further pharmaceutical development of this resource, a combined transcriptome and metabolome analysis was performed in different parts of *D. nobile*. First, saccharides, organic acids, amino acids and their derivatives, and alkaloids were the main substances identified in *D. nobile*. Amino acids and their derivatives and flavonoids accumulated strongly in flowers; saccharides and phenols accumulated strongly in flowers and fruits; alkaloids accumulated strongly in leaves and flowers; and a nucleotide and its derivatives and organic acids accumulated strongly in leaves, flowers, and fruits. Simultaneously, genes for lipid metabolism, terpenoid biosynthesis, and alkaloid biosynthesis were highly expressed in the flowers; genes for phenylpropanoids biosynthesis and flavonoid biosynthesis were highly expressed in the roots; and genes for other metabolisms were highly expressed in the leaves. Furthermore, different members of metabolic enzyme families like cytochrome P450 and 4-coumarate-coA ligase showed differential effects on tissue-specific metabolic accumulation. Members of transcription factor families like AP2-EREBP, bHLH, NAC, MADS, and MYB participated widely in differential accumulation. ATP-binding cassette transporters and some other transporters also showed positive effects on tissue-specific metabolic accumulation. These results systematically elucidated the molecular mechanism of differential accumulation in different parts of *D. nobile* and enriched the library of specialized metabolic products and promising candidate genes.

## 1. Introduction

*Dendrobium nobile* Lindl., one of the endangered orchids, has been used as a medicinal plant for many years in China, Japan, India, and some other countries [1]. It is famous for many health-beneficial bioactivities, such as eye protection, liver protection, cardiovascular protection, gastric protection, and neuro-protection [2].

For sustainable utilization of this valuable resource, pharmaceutical development is required. This is closely related to the chemical composition of *D. nobile*. Recently, more and more bioactive metabolites have been successively revealed in *D. nobile* [1,3]. These research studies focused mainly on polysaccharides, alkaloids, polyphenols, and some other metabolic categories. Polysaccharides of *D. nobile* showed ameliorating effects on spermatogenic disorder, photodamage, and cerebral ischemic injury [4]. Alkaloids from *D. nobile* showed alleviating effects on neurotoxicity, synaptic deficits, and Alzheimer’s disease-like symptoms [5]. Flavonoids and phenolic acids from *D. nobile* showed extracellular and intracellular antioxidant activities [6,7]. However, the systematic composition and distribution of all bioactive components in *D. nobile* were poorly reported.

Moreover, the molecular accumulating mechanism of bioactive metabolites has also attracted the interest of researchers of medicinal plants. Along with revealing *Dendrobium* genomes, more and more genes were identified to play a key role in the biosynthesis of some important components [8,9,10]. Transcriptome sequencing revealed that 30 genes were related to the biosynthesis of the dendrobine sesquiterpene backbone in *D. nobile*, such as *DnAACT*, *DnMVD*, *DnPMK*, and *DnTPS21* [11]. Transcriptomic and metabolic analyses elucidated that some genes played vital roles in the transition from carbohydrate to alkaloid synthesis in the stems of *D. nobile*, such as *DnAROG*, *DnPYK*, *DnDXS*, *DnACEE*, and *DnHMGCR* [12]. Combined transcriptome and metabolome analysis revealed that *DnPHT1* was one of the key genes regulating flavonoid biosynthesis in *D. nobile* [13]. Transcriptome analysis revealed that some genes like *DnPMR6*, *DnPECS-2.1*, *DnSS1*, and *DnGLU3* led to an increase in the biosynthesis of polysaccharides in *D. nobile* [9]. Transcriptome and metabolome analysis revealed that some genes could lead to flavonoid changes in *D. nobile*, such as *DnCHS*, *DnF3′H*, *DnDFR*, and *DnGT1* [14]. But these studies focused only on one or a few classes of metabolites in the stems or flowers of *D. nobile*. So, a more systematic and comprehensive evaluation is needed in detailed parts of *D. nobile*.

For the further pharmaceutical development of this traditional medicinal resource, a systematic and comprehensive understanding of the distribution patterns and accumulation mechanisms of bioactive components is urgently needed. This paper will give some new insights into the biosynthesis, transport, and regulation of bioactive metabolites in *D. nobile* by co-analysis with RNA-seq and high-performance liquid chromatography–tandem mass spectrometry (HPLC-MS/MS).

## 2. Results

### 2.1. Overview of Metabolics and Transcriptomics in Different Parts of D. nobile

**Figure 1:**
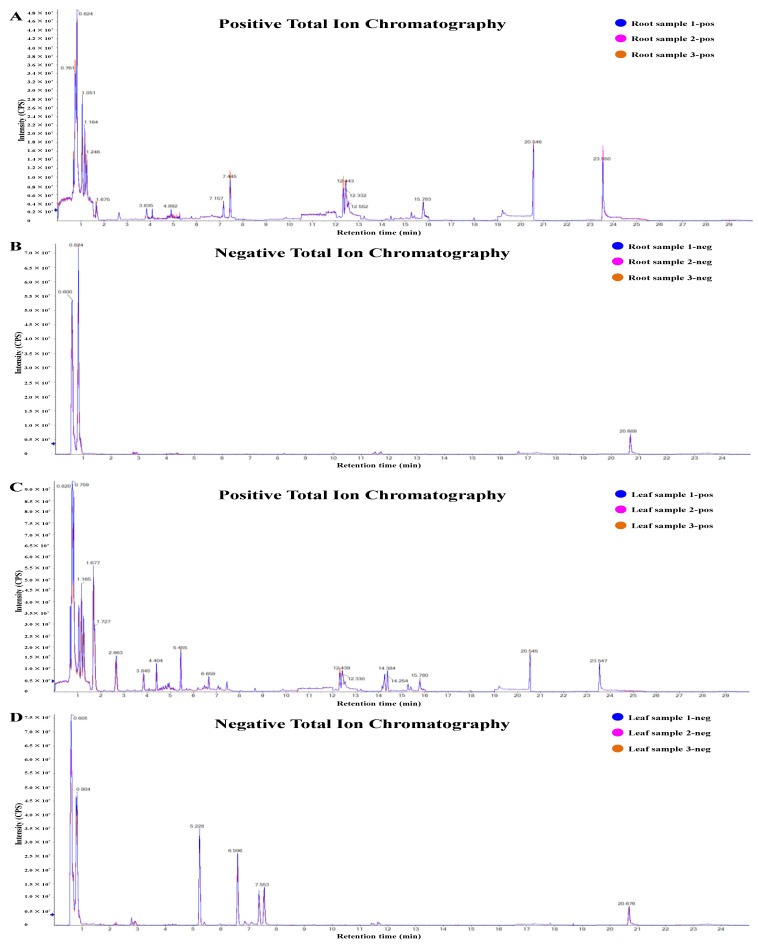
HPLC-MS/MS total ion chromatograms of extracts from different parts of *D. nobile*. (**A**) Positive ion mode of root extracts. (**B**) Negative ion mode of root extracts. (**C**) Positive ion mode of leaf extracts. (**D**) Negative ion mode of leaf extracts. The total ion chromatograms of the stem are shown in Figure S1. The total ion chromatograms of flowers and fruits and the detailed identification information on metabolites are shown in Rao et al. [6,7]. Detailed information on related metabolites is shown in Table S1.

**Figure 2:**
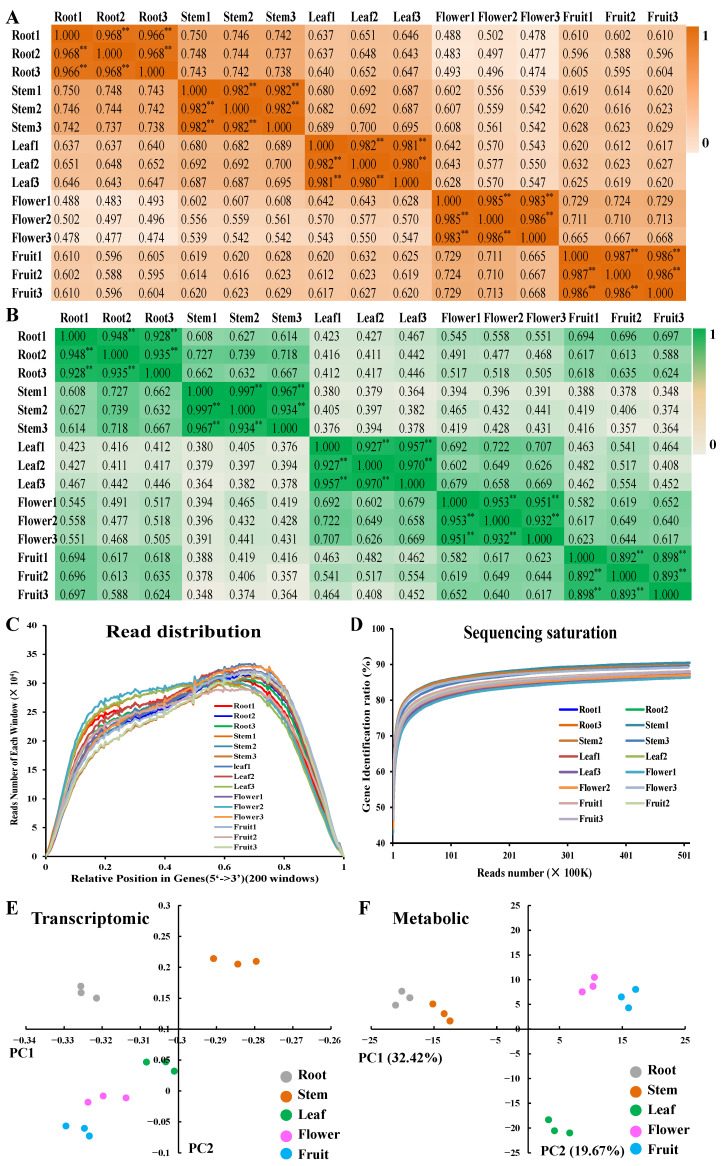
Overview of metabolic and transcriptomic data detected in *D. nobile*. (**A**) Pearson correlation of metabolic data. (**B**) Pearson correlation of transcriptomic data. (**C**) Read distribution randomness of transcriptomic data. (**D**) Sequencing saturation of RNA-seq. (**E**) Principal component analysis of transcriptomic data. (**F**) Principal component analysis of metabolic data. ** *p* < 0.01.

**Figure 3:**
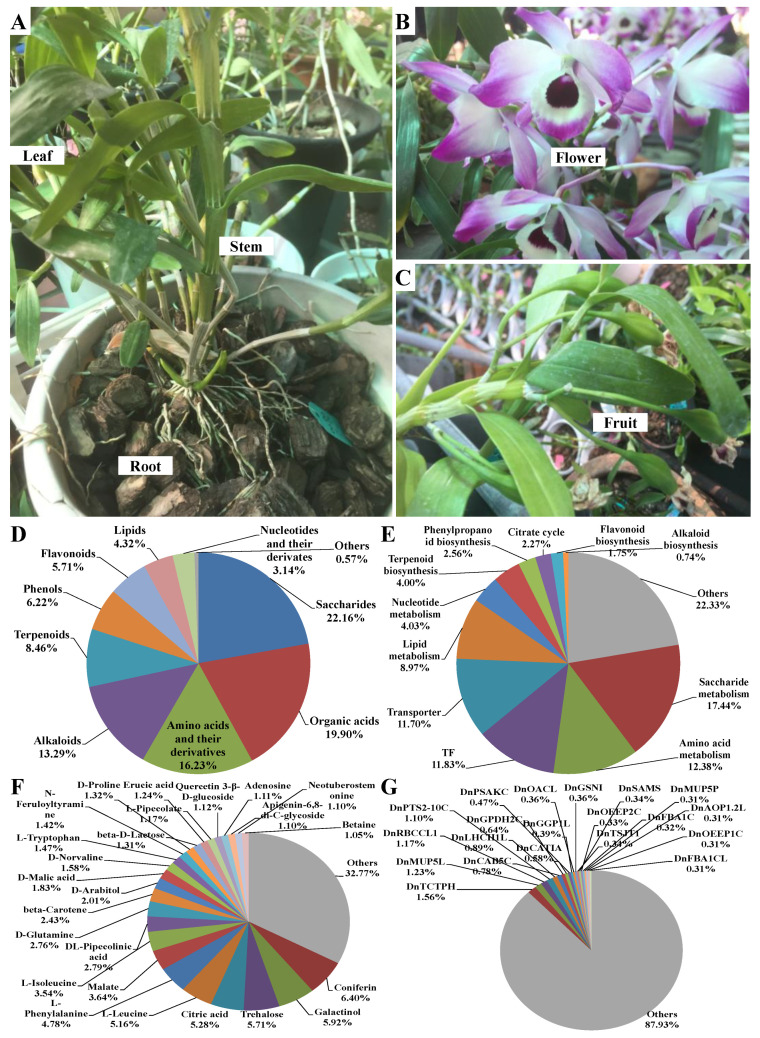
Different parts of *D. nobile* and identified metabolites and genes in *D. nobile*. (**A**) Root, stem, and leaf. (**B**) Flower. (**C**) Fruit. (**D**) Total distribution of different metabolic categories. (**E**) Total expression portion of metabolism-related genes. (**F**) Highly distributed metabolites in *D. nobile*. (**G**) Highly expressed genes in *D. nobile*.

A total of 712 metabolites were finally identified in the methanol extracts from different parts of *D. nobile* by HPLC-MS/MS (Table S1). A good coincidence was obviously observed in the chromatography peaks among three repeated samples in both modes (Figure 1A–D and Figure S1). The Pearson correlation analysis further indicated good consistency in repeated samples (coefficient > 0.96, Figure 2A). But the coefficients between each pair of roots, stems, leaves, flowers, and fruits were less than 0.8. The principal components analysis (PCA) analysis also indicated good consistency within each group and significant differences between the groups (Figure 2B). Simultaneously, a total of 5829 metabolic enzyme coding genes, 1541 transcription factor (TF) coding genes, and 1178 transporter coding genes were identified in the transcriptome of *D. nobile* by RNA-seq (Figure 2C,D). The Pearson correlation analysis and PCA analysis both indicated good consistency between repeated samples and significant differences among different parts of the transcriptomic data (Figure 2E,F). Furthermore, saccharides, organic acids, amino acids and their derivatives, and alkaloids showed a relatively high distribution in *D. nobile* (Figure 3D). Coniferin, galactinol, trehalose, and citric acid were the most highly accumulated metabolites identified in *D. nobile* (Figure 3F). Genes involved in saccharide metabolism, amino acid metabolism, TFs, and transporters showed relatively high expression levels in *D. nobile* (Figure 3E). *DnTCTPH*, *DnMUP5L*, *DnRBCCL1*, and *DnPTS2-10C* were the most active genes detected in *D. nobile* (Figure 3G). These results revealed the general distribution and expression of metabolites and genes in *D. nobile*.

### 2.2. Differentially Accumulated Metabolites and Differentially Expressed Genes in Each Part of D. nobile

**Figure 4:**
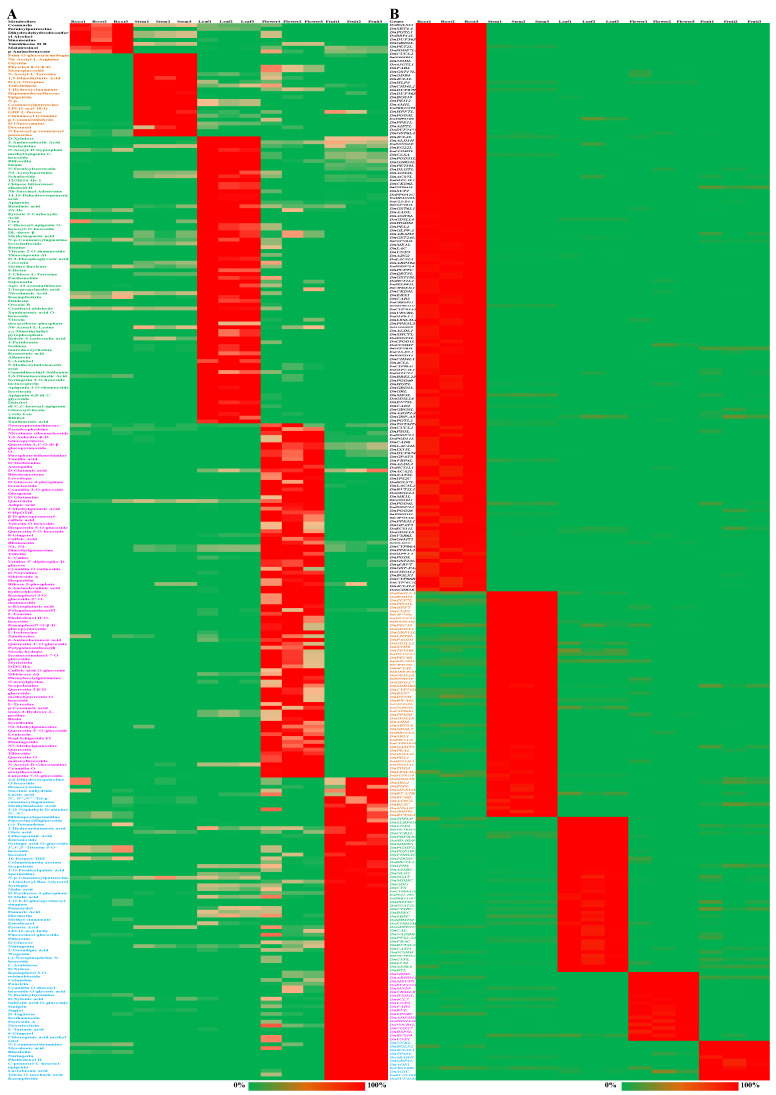
Hitmaps for DAMs and DEGs in different parts of *D. nobile*. (**A**) Significantly accumulated metabolites in the roots, stems, leaves, flowers, and fruits (Log_2_(FC) > 1, *p* < 0.01). (**B**) Significantly highly expressed genes in the roots, stems, leaves, flowers, and fruits (Log_2_(FC) > 2, *p* < 0.01). More details about DEMs and DEGs are shown in Tables S1 and S2.

Overall, 25, 23, 31, 23, and 21 metabolites showed a relative content of more than 1% in the roots, stems, leaves, flowers, and fruits, respectively (Figure S2A,C,E,G,I). Compared with each other part of *D. nobile*, the number of differentially accumulated metabolites (DAMs) was 7, 19, 65, 77, and 70 in the roots, stems, leaves, flowers, and fruits, respectively (Log_2_(FC) > 1, Figure 4A). Taken together, coumarin, feruloylputrescine, tanshinone II B, and p-aminobenzoate were the representative DAMs in the roots; Nα-acetyl-L-arginine, prim-O-glucosylcimifugin, 2-hydroxycinnamate, D-glucosamine, and epigoitrin were the representative DAMs in the stems; apigenin-6,8-di-C-glycoside, isovitexin, betaine, vitexin-2-O-rhamnoside, and di-C,C-hexosyl-apigenin were the representative DAMs in the leaves; L-leucine, L-isoleucine, D-glutamine, quercetin 3-β-D-glucoside, rutin, quercetin-3′-O-glucoside, D-norvaline, myricitrin, and caffeic acid were the representative DAMs in the flowers; and N-feruloyltyramine, D-malic acid, lactobionic acid, malic acid, and N-coumaroyltyramine were the representative DAMs in the fruits (Table S1). Simultaneously, 14, 14, 28, 16, and 12 genes showed a relative expression of more than 0.5% in roots, stems, leaves, flowers, and fruits, respectively (Figure S2B,D,F,H,J). Compared with each other part of *D. nobile*, the number of differentially expressed genes (DEGs) was 158, 63, 43, 19, and 11 in the roots, stems, leaves, flowers, and fruits, respectively (Log_2_(FC) > 2, Figure 4B). Taken together, *DnCCOMT* and *DnTOTMTL* were the representative DEGs in the roots; *DnTPPLP*, *DnRBCCL1*, *DnPTS2-10C*, *DnSHMTM*, *DnCAL*, *DnPTS2-22C*, and *DnFBAC* were the representative DEGs in the leaves; and *DnKCS10* was the representative DEGs in the flowers. No representative DEGs were identified for the stems or fruits (Table S2). These results clearly display the distribution of DAMs and the expression of DEGs in different parts of *D. nobile*.

### 2.3. Distribution of Different Metabolic Categories and Expression of Genes Involved in Different Metabolic Pathways

**Figure 5:**
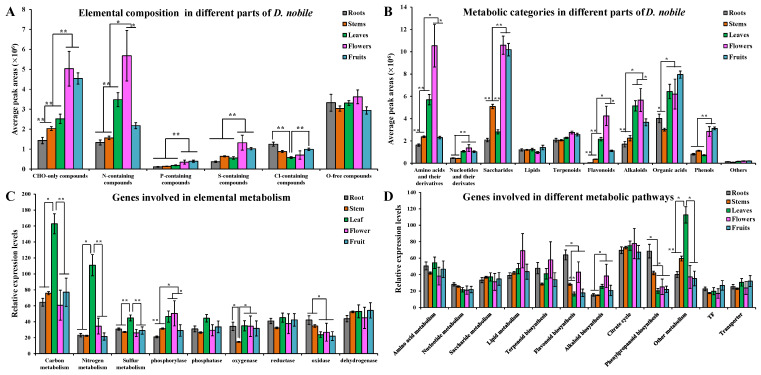
Comparison of element composition- and metabolic category-based distributions and expressions in different parts of *D. nobile*. (**A**) Distribution of metabolites with different element compositions. (**B**) Distribution of different metabolic categories. (**C**) Expression of genes involved in elemental metabolism. (**D**) Expression of genes involved in different metabolic pathways. * *p* < 0.05, ** *p* < 0.01.

Based on element composition, carbon, hydrogen, and oxygen (CHO)-only compounds, phosphorus (P)-containing compounds, and sulfur (S)-containing compounds were significantly accumulated in the flowers and fruits (*p* < 0.01); Nitrogen (N)-containing compounds were significantly accumulated in the flowers and leaves (*p* < 0.05); and chlorine (Cl)-containing compounds were significantly accumulated in the roots (*p* < 0.01, Figure 5A). The genes for carbon metabolism, nitrogen metabolism, sulfur metabolism, and phosphatase families were significantly highly expressed in the leaves (*p* < 0.05); the genes for phosphorylase families were significantly highly expressed in the flowers and leaves (*p* < 0.05); the genes for oxidase families were significantly highly expressed in the roots (*p* < 0.05); and the genes for oxygenase families were significantly lowly expressed in the stems (*p* < 0.05, Figure 5C). Based on compound categories, amino acids and their derivatives and flavonoids were significantly accumulated in the flowers (*p* < 0.05); saccharides and phenols were significantly accumulated in the flowers and fruits (*p* < 0.01); alkaloids were significantly accumulated in the leaves and flowers (*p* < 0.05); and nucleotides and their derivatives and organic acids were significantly accumulated in the leaves, flowers, and fruits (*p* < 0.05, Figure 5B). The genes for alkaloid biosynthesis were significantly highly expressed in the flowers (*p* < 0.05); the genes for phenylpropanoid biosynthesis and flavonoid biosynthesis were significantly highly expressed in the roots (*p* < 0.05); and then genes for other metabolism were significantly highly expressed in the leaves (*p* < 0.05, Figure 5D). These results revealed that different metabolic categories and gene classes showed variant distribution or expression patterns in different parts of *D. nobile*.

### 2.4. Expression of TFs and Transporters in Each Part of D. nobile

**Figure 6:**
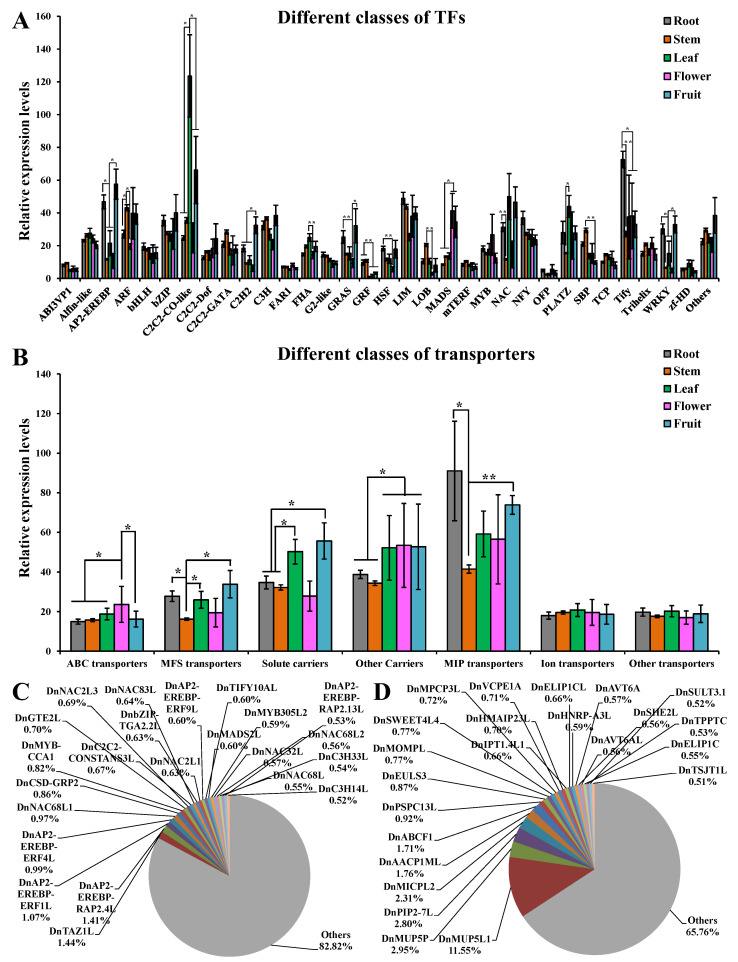
Expression of different TFs and transporter families in different parts of *D. nobile*. (**A**) Average expression of different TF classes in different parts of *D. nobile*. (**B**) Average expression of different transporter families in different parts of *D. nobile*. (**C**) Highly expressed TFs in *D. nobile*. (**D**) Highly expressed transporters in *D. nobile*. * *p* < 0.05, ** *p* < 0.01.

**Figure 7:**
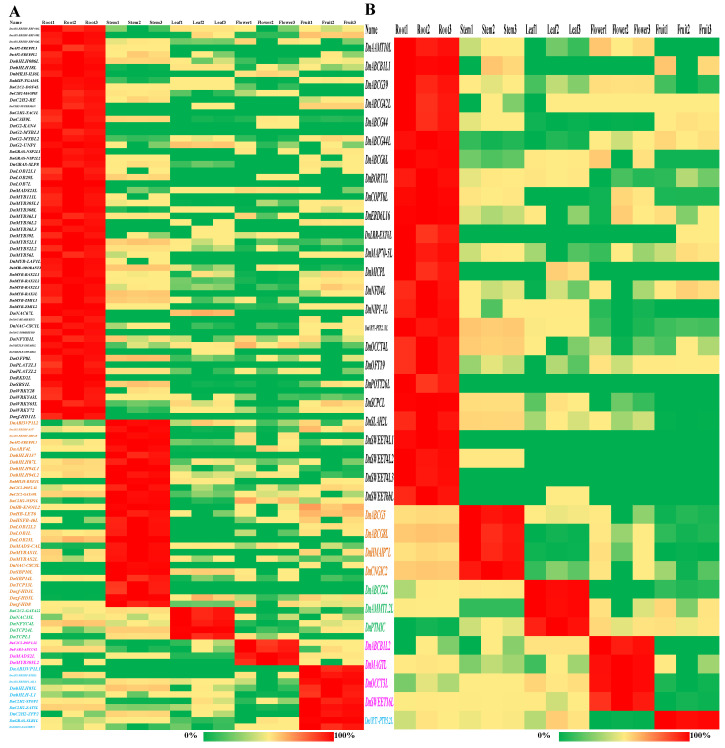
Hitmaps for TFs and transporters in different parts of *D. nobile*. (**A**) Significantly highly expressed TFs in the roots, stems, leaves, flowers, and fruits (Log_2_(FC) > 2, *p* < 0.01). (**B**) Significantly highly expressed transporters in the roots, stems, leaves, flowers, and fruits (Log_2_(FC) > 2, *p* < 0.01).

The TF-coding genes and transporter-coding genes were further classified into different classes. Compared with the other parts, Tify TFs showed significantly higher expression in the roots (*p* < 0.05); LOB and SBP TFs showed significantly higher expression in the stems (*p* < 0.01); C2C2-CO-like and PLATZ TFs showed significantly higher expression in the leaves (*p* < 0.05); and C2H2 TFs showed significantly higher expression in the fruits (*p* < 0.05, Log_2_(FC) > 1, Figure 6A). AP2-EREBP, GRAS, HSF, and WRKY TFs showed significantly higher expression in the roots and fruits (*p* < 0.05); GRF TFs showed significantly higher expression in the roots and stems (*p* < 0.01); MADS TFs showed significantly higher expression in the flowers and fruits (*p* < 0.05); ARF TFs showed significantly higher expression in the stems, flowers, and fruits (*p* < 0.05). ATP-binding cassette (ABC) transporters showed significantly higher expression in the flowers (*p* < 0.05); major facilitator superfamily (MFS) transporters showed significantly higher expression in the fruits (*p* < 0.05); major intrinsic protein (MIP) transporters showed significantly higher expression in the roots and fruits (*p* < 0.05); solute carriers showed significantly higher expression in the leaves and fruits (*p* < 0.05); and other carriers showed significantly higher expression in the leaves, flowers, and fruits (*p* < 0.05, Log_2_(FC) > 1, Figure 6B). Furthermore, *DnTAZ1L*, *DnAP2-EREBP-RAP2.4L*, and *DnAP2-EREBP-ERF1L* showed a relative expression of more than 1% in all TFs (Figure 6C). *DnMUP5L1, DnMUP5P, DnPIP2-7L, DnMICPL2, DnAACP1ML*, and *DnABCF1* showed a relative expression of more than 1% in all transporters (Figure 6D). Overall, 5, 4, 10, 11, and 11 TFs showed a relative expression of more than 1% in the roots, stems, leaves, flowers, and fruits, respectively (Figure S3A,C,E,G,I). Compared with each other part of *D. nobile*, the number of significantly highly expressed TFs was 61, 29, 5, 4, and 10 in the roots, stems, leaves, flowers, and fruits, respectively (Log_2_(FC) > 2, Figure 7A). Taken together, *DnC2C2-DOF1.2L*, *DnMADS2L*, and *DnMYB305L2* were the most specific TFs in the flowers. *DnC2H2-STOP1* was the most specific TF in the fruits. Overall, 6, 11, 9, 10, and 10 transporters showed a relative expression of more than 1% in the roots, stems, leaves, flowers, and fruits, respectively (Figure S3B,D,F,H,J). Compared with each other part of *D. nobile*, the number of significantly highly expressed transporters was 25, 4, 3, 4, and 1 in the roots, stems, leaves, flowers, and fruits, respectively (Log_2_(FC) > 2, Figure 7B). Taken together, *DnHMAIP7L* was the most specific transporter in the stems. These results clearly display the expression of TFs and transporters in different parts of *D. nobile*.

### 2.5. Extremely Significant Associations between Different Metabolic Categories and Gene Classes

As shown in Table 1, the expressions of metabolic enzyme-coding genes were usually positively correlated to the contents of the corresponding metabolites. The genes involved in lipid metabolism and alkaloid biosynthesis were positively and significantly correlated with amino acids and their derivatives, flavonoids, and N-containing compounds; the genes of phosphorylase families were positively and significantly correlated with alkaloids; and the genes for terpenoid biosynthesis were positively and significantly correlated with oxygen (O)-free compounds (correlation coefficient > 0.9, *p* < 0.01). Most of the TF families were negatively correlated with some categories of metabolites. G2-like TFs were significantly and negatively correlated with nucleotides and their derivatives, terpenoids, and CHO-only compounds; GRF TFs were significantly and negatively correlated with nucleotides and their derivatives and alkaloids; SBP TFs were significantly and negatively correlated with organic acids and the other categories; ABI3VP1 TFs were significantly and negatively correlated with organic acids; mTERF TFs were significantly and negatively correlated with the other categories; MYB TFs were significantly and negatively correlated with lipids; and C3H TFs were significantly and negatively correlated with O-free compounds (correlation coefficient < −0.9, *p* < 0.01). However, MADS TFs were positively and significantly correlated with saccharides, terpenoids, phenols, CHO-only compounds, P-containing compounds, and S-containing compounds; and MYB TFs were positively and significantly correlated with O-free compounds (correlation coefficient > 0.9, *p* < 0.01). Most of the transporter-coding genes were positively correlated with some categories of metabolites. ABC transporters were positively and significantly correlated with amino acids and their derivatives, flavonoids, and N-containing compounds; and the other carriers were positively and significantly correlated with nucleotides and their derivatives, organic acids, and the other categories (correlation coefficient > 0.9, *p* < 0.01). However, ion transporters were significantly and negatively correlated with Cl-containing compounds (correlation coefficient < −0.9, *p* < 0.01). These results revealed that the accumulation of some metabolic categories was co-affected by the related biosynthesis and regulating and transporting gene families.

### 2.6. Extremely Significant Associations between DAMs and DEGs

**Figure 8:**
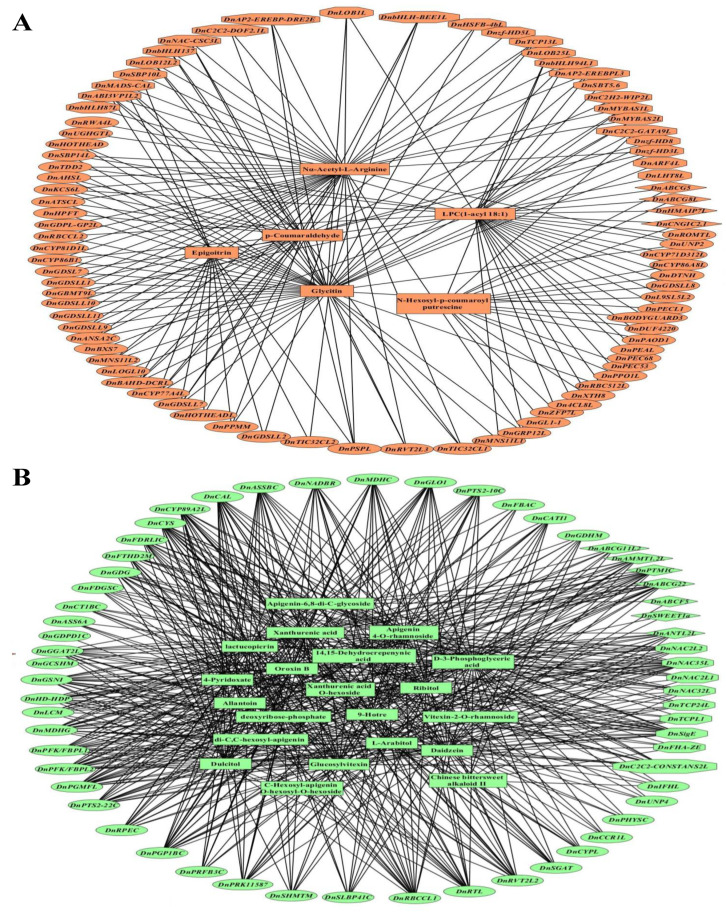
Collinear networks of extremely significantly correlated DAMs and DEGs in the stems (**A**) and leaves (**B**) of *D. nobile*. Square boxes indicate DAMs, ellipse boxes indicate DEGs of metabolic enzyme-coding genes, diamond boxes indicate DEGs of transporters, and octagon boxes indicate DEGs of TFs. Each link means coefficient > 0.99 and *p* < 0.01.

**Figure 9:**
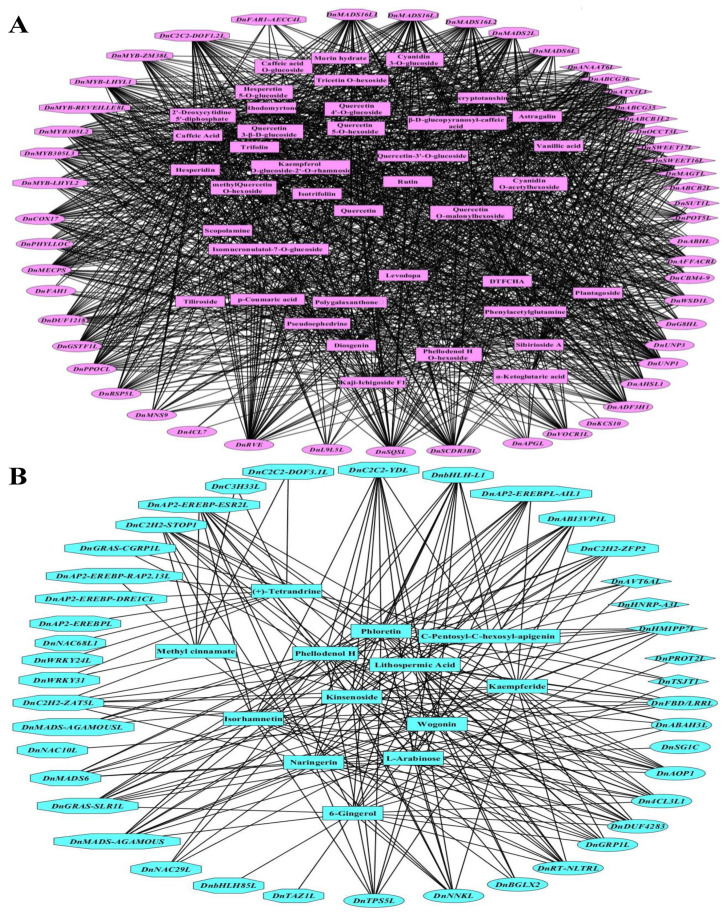
Collinear networks of extremely significantly correlated DAMs and DEGs in the flowers (**A**) and fruits (**B**) of *D. nobile*. Square boxes indicate DAMs, ellipse boxes indicate DEGs of metabolic enzyme coding genes, diamond boxes indicate DEGs of transporters, and octagon boxes indicate DEGs of TFs. Each link means coefficient > 0.99 and *p* < 0.01.

The connection between key DAMs and DEGs was further analyzed in different parts of *D. nobile*. In the roots, only sinomenine showed significantly positive correlations with 12 metabolic enzyme coding genes, such as *DnBGS18*, *DnALD1H*, *DnGST24L*, *DnUGT90A2L*, *DnUGT73B5L*, *DnGDSLL4*, and *DnGDSLL5*, five TFs, such as *DnAP2-EREBP-ERF013L*, *DnbHLH18L*, and *DnNAC67L*, and two transporters, such as *DnBORT1L* (coefficient > 0.99, *p* < 0.01, Figure S4). In the stems, six metabolites, such as glycitin, Nα-acetyl-L-arginine, and p-coumaraldehyde, showed significantly positive correlations with 55 metabolic enzyme-coding genes, such as *DnCYP77A4L*, *DnBAHD-DCRL*, *DnGDSLL7*, *DnPSPL*, and *DnKCS6L*, 26 TFs, such as *DnABI3VP1L2*, *DnMADS-CAL*, *DnAP2-EREBP-DRE2E*, *DnAP2-EREBPL3*, *DnbHLH137*, *DnbHLH94L1*, *DnbHLH-BEE1L*, *DnLOB12L2*, *DnLOB1L*, *DnMYBAS1L*, *DnMYBAS2L*, *DnNAC-CSC3L*, *DnSBP14L*, *DnTCP13L*, and *Dnzf-HD3L*, and four transporters, such as *DnABCG5*, *DnABCG8L*, and *DnHMAIP7L* (coefficient > 0.99, *p* < 0.01, Figure 8A). In the leaves, 21 metabolites, such as D-3-phosphoglyceric acid, daidzein, apigenin 4-O-rhamnoside, xanthurenic acid, ribitol, 9-hotre, dulcitol, and glucosylvitexin, showed significantly positive correlations with 43 metabolic enzyme-coding genes, such as *DnRBCCL1*, *DnGGAT2L*, *DnPFK/FBPL1*, *DnPGP1BC*, *DnMDHG*, *DnPGMFL*, *DnCYS*, *DnCAL*, *DnPFK/FBPL2*, and *DnCYP89A2L*, nine TFs, such as *DnNAC35L*, *DnTCPL1*, and *DnTCP24L*, and seven transporters, such as *DnABCG22*, *DnABCG11L2*, and *DnSWEET1a* (coefficient > 0.99, *p* < 0.01, Figure 8B). In the flowers, 40 metabolites, such as rhodomyrtone, diosgenin, caffeic acid, levodopa, p-coumaric acid, astragalin, quercetin O-malonylhexoside, cyanidin O-acetylhexoside, phellodenol H O-hexoside, pseudoephedrine, hesperidin, rutin, and methylquercetin O-hexoside, showed significantly positive correlations with 26 metabolic enzyme-coding genes, such as *DnMECPS*, *DnRVE*, *DnUNP1*, *DnCOX17*, *DnADF3H1*, *DnUNP3*, *DnWSD1L*, *DnDUF1218*, *DnPHYLLOC*, *DnCBM4-9*, *DnVOCR1L*, *DnG8HL*, *DnRSP5L*, and *DnMNS9*, 13 TFs, such as *DnC2C2-DOF1.2L*, *DnMADS2L*, *DnMYB305L2*, and *DnFAR1-AECC4L*, and 12 transporters, such as *DnSWEET16L*, *DnSWEET17L*, *DnABCB1L2*, *DnABCB2L*, *DnABCG35*, *DnABCG36*, and *DnOCCT3L* (coefficient > 0.99, *p* < 0.01, Figure 9A). In the fruits, 13 metabolites, such as kaempferide, phloretin, kinsenoside, naringerin, isorhamnetin, 6-gingerol, wogonin, and L-arabinose, showed significantly positive correlations with 12 metabolic enzyme-coding genes, such as *DnTPS5L*, *DnSG1C*, *DnAOP1*, *Dn4CL3L1*, *DnFBD/LRRL*, *DnRT-NLTRL*, *DnDUF4283*, *DnNNKL*, and *DnGRP1L*, 25 TFs, such as *DnAP2-EREBP-ESR2L*, *DnbHLH-L1*, *DnC2H2-STOP1*, *DnGRAS-SLR1L*, *DnMADS-AGAMOUS*, *DnAP2-EREBPL-AIL1*, *DnC2H2-ZAT5L*, and *DnC2H2-ZFP2*, and four transporters, such as *DnHMIPP7L* and *DnTSJT1* (coefficient > 0.99, *p* < 0.01, Figure 9B). These results further indicated that the accumulation of some specific DAMs was the result of the corresponding biosynthesis and regulating and transporting DEGs.

### 2.7. Biosynthesis, Regulation, and Transporting of DAMs in Different Parts of D. nobile

**Figure 10:**
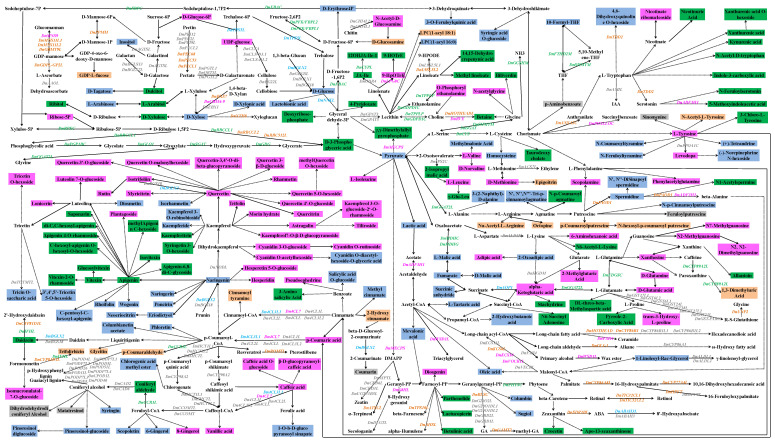
Biosynthesis map of significantly highly accumulated metabolites and highly expressed genes in each part of *D. nobile*. The map is mainly based on KEGG databases (https://www.kegg.jp/, accessed on 10 May 2024). Italic items are genes, and the others are metabolites. Gray indicates metabolites or genes highly distributed or expressed in roots, orange indicates metabolites or genes highly distributed or expressed in stems, green indicates metabolites or genes highly distributed or expressed in leaves, pink indicates metabolites or genes highly distributed or expressed in flowers, and blue indicates metabolites or genes highly distributed or expressed in fruits.

**Figure 11:**
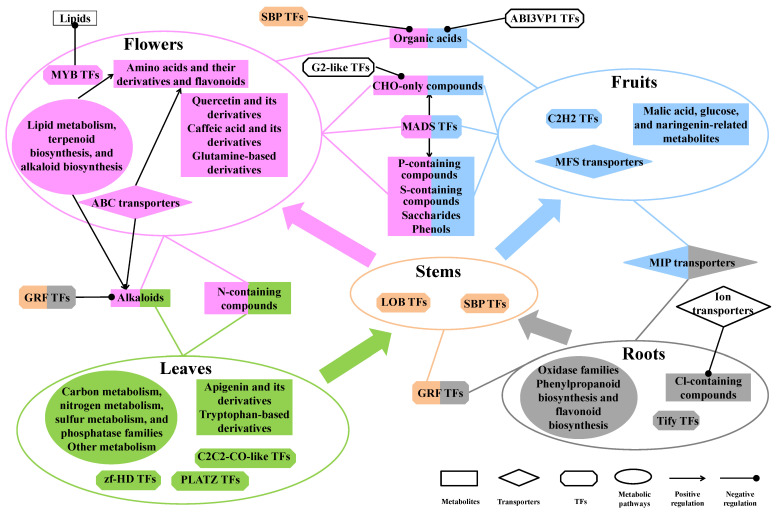
Sketch map showing biosynthesis, regulation, and transportation in different parts of *D. nobile*. Gray indicates metabolites or genes highly distributed or expressed in roots. Orange indicates metabolites or genes highly distributed or expressed in stems. Green indicates metabolites or genes highly distributed or expressed in leaves. Pink indicates metabolites or genes highly distributed or expressed in flowers. Blue indicates metabolites or genes highly distributed or expressed in fruits. The large colored arrows indicate the predicted metabolic accumulation direction in different parts of *D. nobile*.

Most of the DAMs and DEGs in each part of *D. nobile* were placed together in a KEGG-based metabolic map (Figure 10). The metabolites accumulated in the leaves were mainly concentrated in apigenin-based derivatives and tryptophan-based derivatives. The metabolites accumulated in the flowers were mainly concentrated in quercetin-based derivatives, caffeic acid-based derivatives, and glutamine-based derivatives. The metabolites accumulated in the fruits were mainly concentrated in malic acid, glucose, and naringenin-related metabolites. The metabolites accumulated in roots and stems were too few without obvious clustering. The biosynthesis genes showing consistency between expression and content were displayed in this map, such as *DnTPS5L* for D-glucose and *DnAOP1* for succinic anhydride, fumarate, and D/L-malic acid in the fruits; *DnCYPL* for 12OHJA-Ile-1, *DnCCR1L* for coniferyl aldehyde, and *DnGGAT2L*, *DnRBCCL1*, *DnPGP1BC*, *DnGLO1*, *DnSGAT*, and *DnGDG* for D-3-phosphoglyceric acid in the leaves; *DnBGS18* for coumarin in the roots; *DnCYP81D1L* for trifolirhizin in the stems; and *DnLPG8C* for 9-HpOTrE in the flowers. However, many genes involved in the biosynthesis of some specialized metabolites did not show consistency between expression and content, such as *DnAGTSL* (highly expressed in the roots) for inositol (accumulated in the fruits), *DnBXS7* (highly expressed in the stems), *DnCBM4-9* (highly expressed in the flowers), and *DnEBX1* (highly expressed in the roots) for D-xylose (accumulated in the fruits), *DnFTHD2M* (highly expressed in the leaves) for 10-formyl-THF (accumulated in the fruits), *DnPPOA1C* (highly expressed in the roots) for levodopa (accumulated in the flowers), *DnCYP89A2L* (highly expressed in the leaves) for 1,3-dimethyluric acid (accumulated in the stems), and *DnADF3H1* (highly expressed in the flowers) for 5-methoxyindoleacetic acid (accumulated in the leaves). Different members of some other families had different accumulating effects on the metabolites, such as 4-coumarate-coA ligase (4CL) and cytochrome P450 (CYP). Many metabolites showed high accumulation in some specific parts of *D. nobile* but without correlated enzyme-coding genes, such as apigenin-based flavonoids in the leaves and quercetin-based flavonoids in the flowers. Together, the accumulation and regulation of some main DAMs in each part of *D. nobile* are shown in Figure 11. Except for metabolic enzyme-coding genes, TFs and transporters also played important roles in the accumulation of some specific metabolites in different parts of *D. nobile*. MADS TFs, which are highly expressed in flowers and fruits, showed positive effects on CHO-only compounds, P-containing compounds, S-containing compounds, saccharides, and phenols (highly accumulated in the flowers and fruits). SBP TFs, which are highly expressed in the stems, showed negative effects on organic acids (highly accumulated in the flowers and fruits). GRF TFs, which are highly expressed in roots and stems, showed negative effects on alkaloids (highly accumulated in the leaves and flowers). ABC transporters, which are highly expressed in the flowers, showed positive effects on amino acids and their derivatives and flavonoids (highly accumulated in the flowers). These results indicated the co-effects of metabolic enzyme-coding genes, TFs, and transporters on DAMs in different parts of *D. nobile*.

## 3. Discussion

### 3.1. Different Members of Metabolic Enzyme Families Showed Differential Effects on Tissue-Specific Metabolic Accumulation

Metabolic enzyme-coding genes were directly associated with the biosynthesis of secondary metabolites, resulting in their differential accumulation [8]. Here, the expression of metabolic enzyme-coding genes was usually positively correlated with the contents of some specialized compounds. Genes involved in alkaloid biosynthesis were significantly and positively correlated with the contents of N-containing compounds. This was consistent with the previous reports on *Dendrobium* [15]. Some new insights into the biosynthesis and accumulation of some specialized metabolites were also identified. For example, phosphorylase families were found to correlate positively and significantly with the content of alkaloids. This provides more consideration for improving the production of some important chemical components, such as alkaloids, in *Dendrobium*. Specifically, some individual candidate genes that played a key role in the biosynthesis of some specialized metabolites were also indicated. For example, the CYP family played a key role in the biosynthesis of many secondary metabolites [10]. Two new members of CYP, i.e., *DnoNew4*3 and *DnoNew50*, were recently reported to coincide with the distribution of the dendrobine content in *Dendrobium* stems and leaves [16]. Here, *DnCYPL*, a biosynthesis gene for 12OHJA-Ile-1, was highly expressed in the leaves and resulted in the metabolic transformation from JA-Ile (accumulated in the leaves) to 12OHJA-Ile-1 (accumulated in the leaves). *DnCYP81D1L*, a trifolirhizin biosynthesis gene, was highly expressed in the stems and resulted in the metabolic transformation from formononetin to Trifolirhizin (accumulated in the stems). Some other CYP genes, such as *DnCYP86B1*, *DnCYP77A4L, DnCYP86A8L*, *DnCYP71D312L*, and *DnCYP89A2L,* were also correlated with specialized metabolites in different tissues of *D. nobile*. The 4CL family played a key role in the biosynthesis of multiple polyphenols and flavonoids by transforming p-coumaric acid to p-coumaroyl-CoA, cinnamate to cinnamoyl-CoA, and caffeic acid to caffeoyl-CoA [17]. But they were poorly reported in *Dendrobium*. Here, some members of the 4CL family showed differential accumulating effects on specialized metabolites in different parts of *D. nobile*. *Dn4CL8L*, which is highly expressed in the stems, might be correlated with the accumulation of cinnamoyl tyramine, p-coumaraldehyde, and glycitin in the stems. *Dn4CL1L, Dn4CL2L, Dn4CLL*, and *Dn4CL3L2,* which are highly expressed in the roots, might be correlated with the accumulation of dihydrodehydrodiconiferyl alcohol and matairesinol in the roots. *Dn4CL7*, which is highly expressed in the flowers, might be correlated with the accumulation of 8-gingerol, vanillic acid, and a series of flavonoids in the flowers. *Dn4CL3L1*, which is highly expressed in the fruits, might be correlated with the accumulation of 6-gingerol, scopoletin, syringin, pinoresinol glucoside, chlorogenic acid methyl ester, phloretin, columbianetin acetate, and a series of flavonoids in the fruits. Some more candidate genes that played key roles in the differential metabolic accumulation are shown in the biosynthesis map. They could be used to improve the production of the corresponding compounds in pharmaceutical applications by enhancing their biosynthesis levels. However, many metabolic enzyme-coding genes showed no direct correlation with the corresponding metabolites, and many metabolites showed no direct correlation with enzyme-coding genes. For example, genes involved in phenylpropanoid biosynthesis were highly expressed in the roots and stems. But the related products did not accumulate in the roots and stems. A series of flavonoids accumulated in the leaves, flowers, or fruits. But almost no metabolic enzyme-coding genes were found to be the core of their accumulation. These results strongly indicated that some other factors affected the differential accumulation of medicinal components in *D. nobile* in addition to the metabolic-enzyme coding genes [18]. So, the effects of TFs and transporters were further analyzed.

### 3.2. Many TF Families Participated Widely in Tissue-Specific Metabolic Accumulation

In medicinal plants, TFs regulates the spatio-temporal accumulation patterns of some specialized metabolites, such as terpenoids, alkaloids, and phenolic acids, within different plant tissues [18]. Here, many TF subfamilies showed positive or negative effects on the accumulation of some specialized metabolites in different parts of *D. nobile*. The overall expression of G2-like TFs was significantly and negatively correlated with the content of terpenoids. The overall expression of GRF TFs was significantly and negatively correlated with the content of alkaloids. The overall expression of SBP TFs and ABI3VP1 TFs was significantly and negatively correlated with the content of organic acids. The overall expression of MADS TFs was significantly and positively correlated with the contents of saccharides, terpenoids, phenols, CHO-only compounds, P-containing compounds, and S-containing compounds. The overall expression of MYB TFs was significantly and negatively correlated with the content of lipids and was significantly and positively correlated with the content of O-free compounds. Similar regulation patterns were reported in *D. huoshanense*, in which different *DhbHLH* TFs played positive or negative regulator roles for the content of alkaloids [19]. Furthermore, candidate TFs with promising potential for specialized medicinal compounds were also identified. For example, different members of AP2-EREBP TFs were highly correlated with different accumulated metabolites in the roots (*DnAP2-EREBP-ERF013L*), stems (*DnAP2-EREBP-DRE2E* and *DnAP2-EREBPL3*), and fruits (*DnAP2-EREBP-ESR2L* and *DnAP2-EREBPL-AIL1*). Different members of bHLH TFs were highly correlated with different accumulated metabolites in the roots (*DnbHLH18L*), stems (*DnbHLH137, DnbHLH87L, DnbHLH94L1,* and *DnbHLH-BEE1L,*), and fruits (*DnbHLH-L1* and *DnbHLH85L*). Different members of NAC TFs were highly correlated with different accumulated metabolites in the roots (*DnNAC67L*), stems (*DnNAC-CSC3L*), leaves (*DnNAC2L1, DnNAC2L2, DnNAC32L,* and *DnNAC35L*), and fruits (*DnNAC10L, DnNAC29L,* and *DnNAC68L1*). Different members of C2C2 TFs were highly correlated with different accumulated metabolites in the stems (*DnC2C2-DOF2.1L* and *DnC2C2-GATA9L*), leaves (*DnC2C2-CONSTANS2L*), flowers (*DnC2C2-DOF1.2L*), and fruits (*DnC2C2-DOF3.1L* and *DnC2C2-YDL*). Some MADS TFs (*DnMADS2L, DnMADS6L, DnMADS16L1, DnMADS 16L2,* and *DnMADS16L3*) and MYB TFs (*DnMYB305L2*, *DnMYB305L3*, *DnMYB-LHYL1, DnMYB-LHYL2, DnMYB-ZM38L,* and *DnMYB-REVEILLE8L*) showed positive effects on the accumulation of flavonoids like astragalin, quercetin, cyanidin, hesperidin, and rutin in the flowers. These results were partially confirmed in similar research studies in some other *Dendrobium* species. *DoAP2/ERF89* was reported to positively regulate the biosynthesis of β-patchoulene in *D. officinale* [20]. *DcMYB61* showed positive regulation effects on the production of dendrobine in *D. catenatum* [21]. *DoMYB5* and *DobHLH24* could stimulate the accumulation of anthocyanin in *D. officinale* [22]. Two MYB TFs were highly connected with flavonoid biosynthesis in *D. moniliforme* [23]. Some members belonging to the AP2/ERF and MYB TF families were predicted to regulate alkaloid biosynthesis in *D. officinale* [24]. Some TFs of ERF, NAC, and MYB played a significant role in terpenoid backbone biosynthesis in the flower of *D. chrysotoxum* [25]. This study further enriched the candidate TFs with promising potential for the accumulation of some specialized medicinal compounds in *D. nobile*. These regulators could be used for targeted manipulation to enhance the contents of some important medicinal compounds.

### 3.3. ABC Transporters and Some Other Transporters also Showed Positive Effects on Tissue-Specific Metabolic Accumulation

Except for biosynthesis genes and TF regulators, transporters also played an important role in the differential metabolic accumulation in different parts of *D. nobile*. After comparing the ABC transporters, MFS transporters, MIP transporters, ion transporters, solute carriers, other carriers, and other transporters, ABC transporters showed a significant contribution to the differential accumulation of some medicinal compounds in *D. nobile*. This was confirmed in similar research studies in some other plants. For example, ABC transporters showed regulatory effects on flavonoid biosynthesis, transport, and tissue concentration, ultimately resulting in higher flavonoid concentrations in tomato leaves [26]. The ABCC and ABCG subfamilies of ABC transporters were suggested to be important for the accumulation of capsaicin and dihydrocapsaicin in pepper fruits [27]. Some ABCB and ABCG transporters exhibited a high correlation with the cannabinoid content in *Cannabis sativa* L. [28]. ABC transporters were also reported to be important for the accumulation of polysaccharide–zinc complexes in *D. nobile* [9]. Here, the accumulation of amino acids and their derivatives, flavonoids, and N-containing compounds in the leaves and flowers of *D. nobile* was also the result of some ABC transporters. In detail, the high accumulation of glycitin and Nα-acetyl-L-arginine in the stems might be the result of the high expression of *DnABCG5* and *DnABCG8L*. The high accumulation of daidzein, apigenin 4-O-rhamnoside, and glucosylvitexin in the leaves might be the result of the high expression of *DnABCG22* and *DnABCG11L2*. The high accumulation of astragalin, quercetin O-malonylhexoside, cyanidin O-acetylhexoside, hesperidin, rutin, and methylquercetin O-hexoside in the flowers might be the result of the high expression of *DnABCB1L2*, *DnABCB2L*, *DnABCG35*, and *DnABCG36*. Moreover, some other transporters also contributed to differential metabolic accumulation in different parts of *D. nobile*. The SWEET transporters were identified to be related to the accumulation of abundant medicinal compounds in *D. chrysotoxum* [29]. Some members of SWEET transporters, such as *DnSWEET1a*, *DnSWEET16L*, and *DnSWEET17L*, showed a high correlation with the content of some specific metabolites in the leaves and flowers of *D. nobile* in this study. *DnOCCT3L* and *DnTSJT1* of MFS transporters also showed a high correlation with the content of some specific metabolites in the flowers and fruits. These tissue-specific transporter members are responsible for transporting different substances and result in the differential accumulation of medicinal compounds in the roots, stems, leaves, flowers, and fruits of *D. nobile*. These transporters should also be considered to improve the production of target components in the pharmaceutical development of *D. nobile*.

## 4. Materials and Methods

### 4.1. Plant Materials

A line of *D. nobile*, named JC-1, was cultivated at Hejiang, Sichuan Province (28°49′ N, 105°50′ E) for continuous sampling. Roots, stems, leaves, and flowers were collected in May 2019, May 2020, and May 2021, and fruits were collected in November 2019, November 2020, and November 2021. The tissue samples were obtained from more than 30 individual plants for each collection. The tissue samples were washed with pure water, dried at 40 °C for a week, ground into a powder, and screened using a 50-mesh sieve for the extraction of metabolites. The remaining fresh samples were stored at −80 °C before RNA extraction.

### 4.2. Metabolite Extraction for HPLC-MS/MS

Each 100 mg fine powder sample was suspended with a 500 μL prechilled solution (80% methanol contained 0.1% formic acid) by well vortexing. The sample was incubated for 5 min and then centrifuged at 15,000 rpm for 10 min. The supernatant was diluted to a final concentration of 53% methanol by pure water. The sample was then transferred to a new tube and centrifuged at 15,000 rpm for 20 min. The supernatant was used for chromatography [6].

### 4.3. HPLC-MS/MS Analysis

HPLC-MS/MS analyses were performed using an ExionLC™ AD system coupled with a QTRAP^®^ 6500+ mass spectrometer (AB Sciex Pte. Ltd., Framingham, MA, USA) in Novogene Co., Ltd. (Beijing, China). The positive ion mode was as follows: the sample was injected onto a BEH C8 column (100 mm × 2.1 mm, 1.9 μm) using a 30 min linear gradient at a flow rate of 0.35 mL/min in the positive polarity mode. The eluents were eluent A (0.1% formic acid–water) and eluent B (0.1% formic acid–acetonitrile). The solvent gradient was established as follows: 5% B, 1 min; 5–100% B, 24.0 min; 100% B, 28.0 min; 100–5% B, 28.1 min; and 5% B, 30 min. A QTRAP^®^ 6500+ mass spectrometer was operated in positive polarity mode with a curtain gas of 35 psi, a collision gas of medium, an ionspray voltage of 5500 V, a temperature of 500 °C, an ion source gas of 1:55, and an ion source gas of 2:55. The negative ion mode was as follows: the sample was injected onto an HSS T3 column (100 mm × 2.1 mm) using a 25 min linear gradient at a flow rate of 0.35 mL/min in the negative polarity mode. The eluents were eluent A (0.1% formic acid–water) and eluent B (0.1% formic acid–acetonitrile). The solvent gradient was set as follows: 2% B, 1 min; 2–100% B, 18.0 min; 100% B, 22.0 min; 100–5% B, 22.1 min; and 5% B, 25 min. The QTRAP^®^ 6500+ mass spectrometer was operated in positive polarity mode with a curtain gas of 35 psi, a collision gas of medium, an ionspray voltage of −4500 V, a temperature of 500 °C, an ion source gas of 1:55, and an ion source gas of 2:55 [7].

### 4.4. novoDB Database of Standards

Chemical standards were used for key parameter collection under the chromatographic and mass spectrometry conditions above. Finally, a total of six parameters, including the parent ion (Q1), the daughter ion (Q3), declustering potential (DP), collision energy (CE), molecular weight (MW), and retention time (RT), were stored for each specific compound in the novoDB database. Then, it was used for quasi-targeted metabolic analysis under a certain LC-MS/MS method [30]. Currently, the novoDB database has been expanded to 3250+ plant compounds (https://cn.novogene.com/, accessed on 10 May 2024).

### 4.5. Metabolites Identification by Multiple Reaction Monitoring

To identify metabolites in the extracts of *D. nobile* quickly, accurately, and extensively, MRM was used for scanning mainly based on the above six key parameters [7,30]. For the Q1/Q3 scan, ± 0.7 was set; 0–300 was set for the DP scan; ± 150 was set for the CE scan; and ± 0.01 was set for the RT scan. If a compound matched a standard within the set scanning channel, this compound was detected as the standard. The MS parameters and chromatographic signals of all matched compounds were exported as a raw data file for further analysis.

### 4.6. Metabolite Quantification

The data files generated by HPLC-MS/MS were processed using SCIEX OS Version 1.4 (AB Sciex Pte. Ltd., Framingham, MA, USA) to integrate and correct the peak. The main parameters were established as a minimum peak height of 500, a signal/noise ratio of 5, and a Gaussian smooth width of 1. The screened signal peaks were used for peak area integration. The peak area of Q3 was used for relative quantification of the corresponding metabolite [31].

### 4.7. Metabolite Annotation and Classification

The metabolites were further annotated using the KEGG database (http://www.genome.jp/kegg/, accessed on 10 May 2024), the HMDB database (http://www.hmdb.ca/, accessed on 10 May 2024), and the Lipidmaps database (http://www.lipidmaps.org/, accessed on 10 May 2024) [7]. These annotations were used for a final classification of each compound (Table S1). Based on element composition, the identified metabolites were mainly classified into 6 classes, including CHO-only compounds (only containing carbon, hydrogen, and oxygen elements), N-containing compounds (containing nitrogen elements), P-containing compounds (containing phosphorus elements), S-containing compounds (containing sulfur elements), Cl-containing compounds (containing chlorine elements), and O-free compounds (oxygen-free). Based on the compound properties, the identified metabolites were mainly classified into 10 categories, including saccharides, organic acids, alkaloids, terpenoids, phenols, flavonoids, lipids, amino acids and their derivatives, nucleotides and their derivatives, and others.

### 4.8. Total RNA Extraction

The ethanol precipitation protocol and CTAB-PBIOZOL reagent (BIOER, Hangzhou, China) were used for the purification of total RNA from the plant tissue according to the manual instructions. Tissue samples of approximately 80 mg were ground to powder within liquid nitrogen and then transferred to a 1.5 mL centrifuge tube with preheated CTAB-pBIOZOL at 65 °C. After incubation in a thermo mixer (EPPENDORF, Hamburg, Germany) at 65 °C for 15 min, the samples were centrifuged at 12,000× *g* for 5 min at 4 °C. Then, 400 μL chloroform, 700 μL acidic phenol mixed with 200 μL chloroform, and an equal volume of chloroform were added to the supernatant and centrifuged at 12,000× *g* for 10 min at 4 °C, successively. An equal volume of isopropyl alcohol was added to the supernatant and placed at −20 °C for 2 h. The mix was centrifuged at 12,000× *g* for 20 min at 4 °C. After being washed with 1 mL of 75% ethanol, the RNA pellet was air-dried in a biosafety cabinet and dissolved with 50 μL of DEPC (diethyl pyrocarbonate)-treated water. Subsequently, total RNA was qualified and quantified using a Nano Drop and Agilent 2100 bioanalyzer (Thermo Fisher, Waltham, MA, USA).

### 4.9. RNA-Seq

The mRNA was modified from the total RNA by oligo (dT), RNaseH, and DNaseI. The cDNA library was constructed from mRNA using random hexamer (N6) primers. The cDNA libraries were sequenced on a BGISEQ-500 RS platform (BGI, Shenzhen, China). Quality control checks were conducted by SOAPnuke v1.5.2. The clean reads were separated from the raw data by removing adaptor sequences, reads with more than 5% of unknown bases, and low-quality reads (the ratio of bases with quality value less than 10 to total bases was more than 20%). The expression levels of each unigene were quantified by the RSEM software package v1.3.1 and presented as fragments per kilobase million (FPKM).

### 4.10. Genes Annotation

The high-quality clean reads were then mapped to the reference genome of Dendrobium catenatum (GCA_001605985.2) via HISAT2 software v2.1.0. Bowtie2 software v2.3.4 was employed for blasting clean reads to reference gene sequences. Four gene/protein databases were used for functional annotation, including PlantTFDB (http://plntfdb.bio.uni-potsdam.de/v3.0/, accessed on 10 May 2024), the KEGG GENES Database (https://www.kegg.jp/blastkoala/, accessed on 10 May 2024), the STRING database (https://string-db.org/, accessed on 10 May 2024), and the NCBI gene database (https://www.ncbi.nlm.nih.gov/gene, accessed on 10 May 2024). Detailed annotation information and gene abbreviations are shown in Table S2.

### 4.11. Statistical Analysis

All measurements and experiments were repeated three times, and the data were expressed as the mean ± standard deviation (SD). Log_2_(fold change) (Log_2_(FC)) was used for the comparison of metabolic and transcriptomic data. Correlation analysis was performed using PASW statistics 18.0 (International Business Machines Corporation, New York, NY, USA). Collinear analysis of the correlated data was performed using Cytoscape v3.7.1 (National Resource for Network Biology, College Park, MD, USA). Pearson’s correlation coefficients and *p*-values were used to evaluate the correlations. Student’s t-test was used for comparison between two groups. One-way analysis of variance was used for comparison among three or more groups.

## 5. Conclusions

The metabolic accumulation pattern and transcriptomic expression pattern were systematically compared in the roots, stems, leaves, flowers, and fruits of *D. nobile*. The co-effects of metabolic enzyme-coding genes, transcription factors, and transporters contributed to the differential accumulation of medicinal components in different parts of *D. nobile*. Further studies on these interactions will be beneficial for enhancing the production of target compounds and the promising pharmaceutical development of *D. nobile*.

## Figures and Tables

**Table 1:** Co-relationships among different metabolic categories and gene classes in *D. nobile.*

Name	Amino Acids and Their Derivatives	Nucleotides and their Derivatives	Saccharides	Lipids	Terpenoids	Flavonoids	Alkaloids	Organic acids	Phenols	Other Categories	CHO-Only Compounds	N-Containing Compounds	P-Containing Compounds	S-Containing Compounds	Cl-Containing Compounds	O-Free Compounds
All	0.016	0.082	−0.623	0.174	−0.301	0.012	0.291	0.126	−0.624	−0.066	−0.389	0.025	−0.386	−0.504	−0.507	0.069
Metabolic genes	0.145	0.043	−0.658	−0.068	−0.348	0.094	0.303	−0.070	−0.707	−0.215	−0.441	0.119	−0.502	−0.488	−0.587	0.222
Carbon metabolism	0.068	0.208	−0.449	0.236	−0.165	0.093	0.407	0.263	−0.468	0.037	−0.222	0.095	−0.212	−0.344	−0.633	−0.028
Nitrogen metabolism	0.310	0.358	−0.400	−0.018	−0.030	0.315	0.565	0.277	−0.429	0.130	−0.129	0.325	−0.176	−0.229	−0.719	0.256
Sulfur metabolism	−0.009	0.126	−0.586	0.246	−0.245	0.007	0.301	0.228	−0.560	0.035	−0.338	0.017	−0.312	−0.483	−0.455	0.050
Phosphorylase	0.913 *	0.802	0.337	−0.585	0.598	0.912 *	0.936 **	0.374	0.219	0.421	0.533	0.914 *	0.358	0.565	−0.931 *	0.606
Phosphatase	0.098	0.373	−0.331	0.308	0.038	0.165	0.476	0.524	−0.276	0.344	−0.056	0.161	−0.006	−0.245	−0.486	0.059
Oxygenase	0.384	0.588	0.065	−0.085	0.479	0.446	0.492	0.634	0.197	0.735	0.318	0.449	0.331	0.159	−0.059	0.544
Reductase	0.003	0.394	−0.177	0.401	0.191	0.104	0.349	0.680	−0.033	0.582	0.078	0.094	0.186	−0.156	−0.107	0.079
Oxidase	−0.428	−0.812 *	−0.590	−0.209	−0.708	−0.570	−0.788	−0.858 *	−0.571	−0.729	−0.735	−0.528	−0.759	−0.614	0.688	0.093
Dehydrogenase	−0.330	0.034	0.085	0.694	−0.059	−0.224	0.084	0.326	0.053	0.016	0.068	−0.264	0.190	−0.029	−0.351	−0.761
Amino acid metabolism	−0.440	−0.204	−0.709	0.546	−0.457	−0.410	−0.137	0.142	−0.593	−0.046	−0.538	−0.402	−0.411	−0.724	0.080	−0.190
Nucleotide metabolism	−0.666	−0.923 *	−0.641	0.073	−0.812*	−0.778	−0.920 *	−0.806 *	−0.595	−0.756	−0.809 *	−0.745	−0.766	−0.731	0.786	−0.172
Saccharide metabolism	−0.500	−0.381	−0.501	0.563	−0.587	−0.490	−0.202	−0.163	−0.550	−0.475	−0.507	−0.499	−0.424	−0.557	−0.256	−0.640
Lipid metabolism	0.973 **	0.802	0.624	−0.774	0.799	0.966 **	0.804	0.331	0.538	0.539	0.734	0.968 **	0.548	0.815 *	−0.582	0.752
Terpenoid biosynthesis	0.724	0.513	0.168	−0.738	0.491	0.679	0.474	0.164	0.193	0.446	0.330	0.706	0.185	0.357	−0.108	0.941 **
Flavonoid biosynthesis	−0.017	−0.357	−0.258	−0.469	−0.217	−0.129	−0.424	−0.504	−0.200	−0.238	−0.313	−0.089	−0.376	−0.221	0.626	0.503
Alkaloid biosynthesis	0.975 **	0.911 *	0.578	−0.654	0.843 *	0.996 **	0.913 *	0.512	0.520	0.679	0.755	0.995 **	0.596	0.775	−0.656	0.745
Citrate cycle	0.866 *	0.482	0.079	−0.851 *	0.296	0.777	0.676	−0.091	−0.074	0.031	0.215	0.803	−0.022	0.343	−0.776	0.760
Phenylpropanoid biosynthesis	−0.528	−0.807 *	−0.582	−0.060	−0.685	−0.643	−0.832 *	−0.742	−0.518	−0.626	−0.717	−0.606	−0.695	−0.642	0.807 *	0.020
Other metabolic genes	0.077	0.063	−0.562	0.083	−0.318	0.055	0.318	0.012	−0.623	−0.195	−0.369	0.068	−0.403	−0.430	−0.651	0.027
TFs	−0.592	−0.060	0.106	0.838 *	0.060	−0.450	−0.286	0.514	0.290	0.399	0.093	−0.485	0.329	−0.111	0.550	−0.574
ABI3VP1	−0.490	−0.872 *	−0.394	−0.118	−0.720	−0.621	−0.813 *	−0.940 **	−0.456	−0.895 *	−0.645	−0.595	−0.672	−0.466	0.519	−0.231
Alfin-like	0.255	−0.098	−0.535	−0.382	−0.401	0.142	0.211	−0.434	−0.684	−0.517	−0.440	0.174	−0.588	−0.346	−0.642	0.260
AP2-EREBP	−0.618	−0.158	0.014	0.755	−0.019	−0.501	−0.391	0.394	0.212	0.327	−0.007	−0.527	0.218	−0.195	0.685	−0.479
ARF	0.040	−0.015	0.703	−0.117	0.304	0.055	−0.135	−0.112	0.622	−0.030	0.453	0.031	0.430	0.593	0.142	−0.268
bHLH	−0.692	−0.907 *	−0.631	0.123	−0.790	−0.792	−0.925 *	−0.751	−0.567	−0.703	−0.791	−0.762	−0.732	−0.732	0.828*	−0.187
bZIP	−0.592	−0.172	0.230	0.679	0.076	−0.477	−0.446	0.333	0.404	0.310	0.128	−0.508	0.340	−0.009	0.756	−0.524
C2C2-CO-like	0.006	0.313	−0.257	0.433	−0.009	0.085	0.440	0.490	−0.244	0.235	−0.040	0.070	0.024	−0.209	−0.583	−0.189
C2C2-Dof	0.044	0.468	0.869 *	0.385	0.679	0.200	0.257	0.680	0.899 *	0.633	0.794	0.145	0.896 *	0.720	−0.028	−0.424
C2C2-GATA	−0.513	−0.810 *	−0.361	0.049	−0.727	−0.617	−0.693	−0.845 *	−0.466	−0.918 *	−0.606	−0.603	−0.618	−0.442	0.249	−0.456
C2H2	−0.593	−0.053	0.219	0.849 *	0.106	−0.443	−0.290	0.514	0.385	0.394	0.169	−0.484	0.405	−0.018	0.536	−0.651
C3H	−0.917 *	−0.641	−0.117	0.833 *	−0.476	−0.858 *	−0.739	−0.154	−0.055	−0.350	−0.328	−0.885 *	−0.124	−0.381	0.582	−0.956 **
FAR1	0.471	0.074	0.397	−0.785	0.298	0.381	0.011	−0.340	0.330	−0.034	0.281	0.401	0.121	0.474	0.157	0.564
FHA	−0.172	0.068	−0.311	0.498	−0.220	−0.115	0.235	0.246	−0.345	−0.060	−0.189	−0.131	−0.123	−0.303	−0.554	−0.423
G2-like	−0.668	−0.946 **	−0.842 *	0.112	−0.963 **	−0.792	−0.841 *	−0.853 *	−0.832 *	−0.888 *	−0.963 **	−0.756	−0.934 *	−0.892 *	0.547	−0.214
GRAS	−0.738	−0.270	0.010	0.855 *	−0.113	−0.620	−0.489	0.316	0.188	0.202	−0.061	−0.651	0.181	−0.233	0.691	−0.660
GRF	−0.694	−0.957 **	−0.470	0.112	−0.793	−0.799	−0.942 **	−0.864 *	−0.479	−0.855 *	−0.719	−0.777	−0.681	−0.595	0.695	−0.377
HSF	−0.882 *	−0.535	−0.355	0.833 *	−0.451	−0.815 *	−0.676	0.037	−0.185	−0.099	−0.421	−0.829 *	−0.191	−0.575	0.749	−0.642
LIM	−0.477	−0.636	−0.003	0.000	−0.310	−0.533	−0.791	−0.541	0.048	−0.390	−0.255	−0.524	−0.210	−0.153	0.881 *	−0.183
LOB	−0.650	−0.812 *	−0.527	0.305	−0.829 *	−0.724	−0.663	−0.712	−0.608	−0.893 *	−0.713	−0.715	−0.674	−0.621	0.160	−0.615
MADS	0.626	0.810 *	0.968 **	−0.233	0.968 **	0.727	0.643	0.675	0.956 **	0.801	0.991 **	0.693	0.949 **	0.983 **	−0.292	0.231
mTERF	−0.459	−0.792	−0.480	0.000	−0.781	−0.576	−0.624	−0.867 *	−0.597	−0.956 **	−0.682	−0.557	−0.714	−0.522	0.133	−0.379
MYB	0.879 *	0.514	0.221	−0.943 **	0.471	0.797	0.562	−0.015	0.154	0.259	0.344	0.827 *	0.127	0.455	−0.366	0.967 **
NAC	−0.268	0.226	−0.191	0.697	0.045	−0.141	0.181	0.655	−0.053	0.453	−0.001	−0.164	0.166	−0.250	−0.057	−0.292
NFY	−0.439	−0.678	−0.749	−0.056	−0.679	−0.548	−0.664	−0.604	−0.661	−0.510	−0.771	−0.506	−0.752	−0.754	0.670	0.173
OFP	0.492	0.314	0.335	−0.617	0.474	0.458	0.167	0.071	0.388	0.392	0.367	0.476	0.271	0.419	0.268	0.728
PLATZ	0.236	0.461	−0.295	0.139	0.144	0.291	0.537	0.547	−0.222	0.449	0.009	0.295	0.031	−0.175	−0.432	0.286
SBP	−0.321	−0.784	−0.449	−0.261	−0.720	−0.473	−0.643	−0.973 **	−0.560	−0.946 **	−0.658	−0.441	−0.736	−0.455	0.252	−0.117
TCP	0.319	−0.133	−0.300	−0.540	−0.314	0.189	0.146	−0.571	−0.492	−0.591	−0.305	0.217	−0.488	−0.130	−0.586	0.246
Tify	−0.245	−0.360	−0.512	−0.084	−0.337	−0.308	−0.430	−0.264	−0.375	−0.113	−0.467	−0.273	−0.437	−0.505	0.660	0.343
Trihelix	0.602	0.163	0.339	−0.780	0.215	0.504	0.278	−0.374	0.151	−0.226	0.251	0.518	0.039	0.477	−0.444	0.405
WRKY	−0.648	−0.214	−0.126	0.753	−0.119	−0.545	−0.419	0.338	0.075	0.261	−0.125	−0.565	0.098	−0.319	0.683	−0.446
zf-HD	0.429	0.235	−0.485	−0.341	−0.143	0.364	0.502	−0.042	−0.568	−0.113	−0.252	0.392	−0.379	−0.257	−0.735	0.458
Other TFs	−0.561	−0.087	0.358	0.841 *	0.065	−0.420	−0.232	0.375	0.410	0.161	0.223	−0.471	0.421	0.116	0.208	−0.905 *
Transporters	−0.200	0.367	0.080	0.735	0.243	−0.040	0.284	0.799	0.201	0.588	0.238	−0.076	0.412	−0.007	−0.136	−0.391
ABC transporters	0.997 **	0.846 *	0.526	−0.747	0.769	0.992 **	0.883 *	0.379	0.441	0.554	0.686	0.996 **	0.499	0.742	−0.691	0.778
MFS transporters	−0.395	0.143	0.055	0.733	0.167	−0.250	−0.053	0.659	0.247	0.557	0.144	−0.280	0.353	−0.093	0.371	−0.351
Solute carriers	−0.354	0.214	0.025	0.844 *	0.104	−0.197	0.147	0.692	0.131	0.434	0.134	−0.237	0.327	−0.098	−0.093	−0.571
Other carriers	0.617	0.951 **	0.557	−0.022	0.850 *	0.744	0.873 *	0.936 **	0.599	0.943 **	0.784	0.717	0.780	0.640	−0.541	0.314
MIP transporters	−0.367	−0.144	−0.211	0.326	−0.065	−0.324	−0.332	0.202	−0.009	0.274	−0.156	−0.320	−0.020	−0.303	0.706	0.037
Ion transporters	0.505	0.427	−0.079	−0.233	0.113	0.493	0.669	0.153	−0.216	0.021	0.091	0.495	−0.026	0.102	−0.955 **	0.203
Other transporters	−0.477	−0.214	−0.676	0.581	−0.434	−0.439	−0.179	0.169	−0.541	−0.006	−0.514	−0.434	−0.372	−0.709	0.170	−0.207

* *p* < 0.05, ** *p* < 0.01. The coefficient values of more than 0.9 and *p* < 0.01 are highlighted in bold.

## Data Availability

The datasets generated by RNA-seq for this study can be found in the NCBI databases under the accession of PRJNA721927 (https://www.ncbi.nlm.nih.gov/bioproject/, release date 1 May 2024).

## Supplementary Materials

The following supporting information can be downloaded at: https://www.mdpi.com/article/10.3390/ijms25105356/s1.
