# Supplementary material for: Metabolic and Transcriptomic Profile Revealing the Differential Accumulating Mechanism in Different Parts of Dendrobium nobile
Source: Int J Mol Sci. 2024 May 14;25(10):5356. doi: 10.3390/ijms25105356 (PMC11121218; doi:10.3390/ijms25105356)
Supplement: Supplementary file 1 [file ijms-25-05356-s001.zip › Figure S1.pdf]

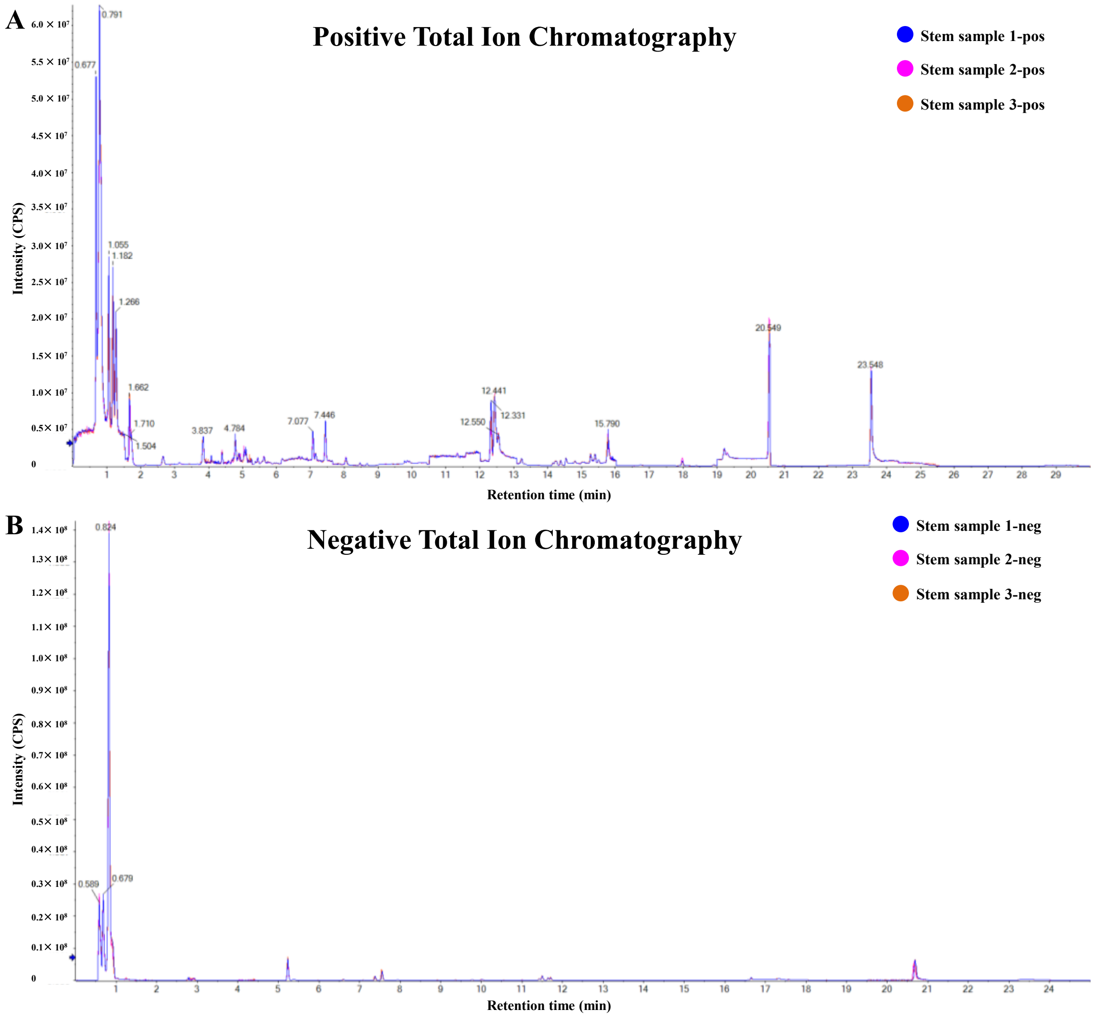

Figure S1. HPLC-MS/MS total ion chromatograms of extracts from stems of *D. nobile*. (**A**) Positive ion mode. (**B**) Negative ion mode.
