# Supplementary material for: Metabolic and Transcriptomic Profile Revealing the Differential Accumulating Mechanism in Different Parts of Dendrobium nobile
Source: Int J Mol Sci. 2024 May 14;25(10):5356. doi: 10.3390/ijms25105356 (PMC11121218; doi:10.3390/ijms25105356)
Supplement: Supplementary file 1 [file ijms-25-05356-s001.zip › Figure S2.pdf]

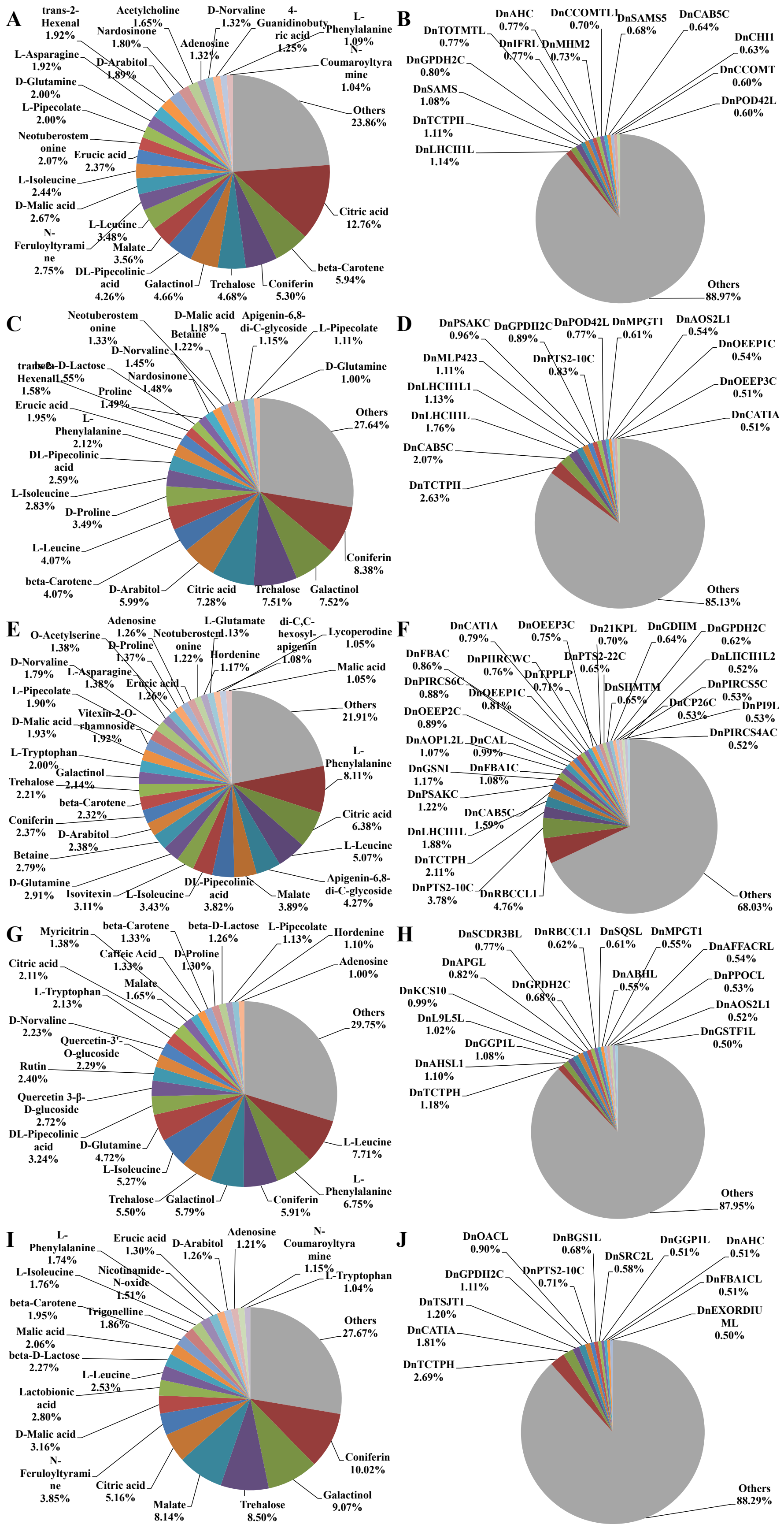

Figure S2. Highly distributed metabolites and highly expressed genes in different parts of *D. nobile*. Highly accumulated metabolites in roots (**A**), stems (**C**), leaves (**E**), flowers (**G**), and fruits (**I**). Highly expressed genes in roots (**B**), stems (**D**), leaves (**F**), flowers (**H**), and fruits (**J**). Only the metabolites with more than 1% distribution portion and the genes with more than 0.5% expression portion were shown here.
