# Supplementary material for: Metabolic and Transcriptomic Profile Revealing the Differential Accumulating Mechanism in Different Parts of Dendrobium nobile
Source: Int J Mol Sci. 2024 May 14;25(10):5356. doi: 10.3390/ijms25105356 (PMC11121218; doi:10.3390/ijms25105356)
Supplement: Supplementary file 1 [file ijms-25-05356-s001.zip › Figure S4.pdf]

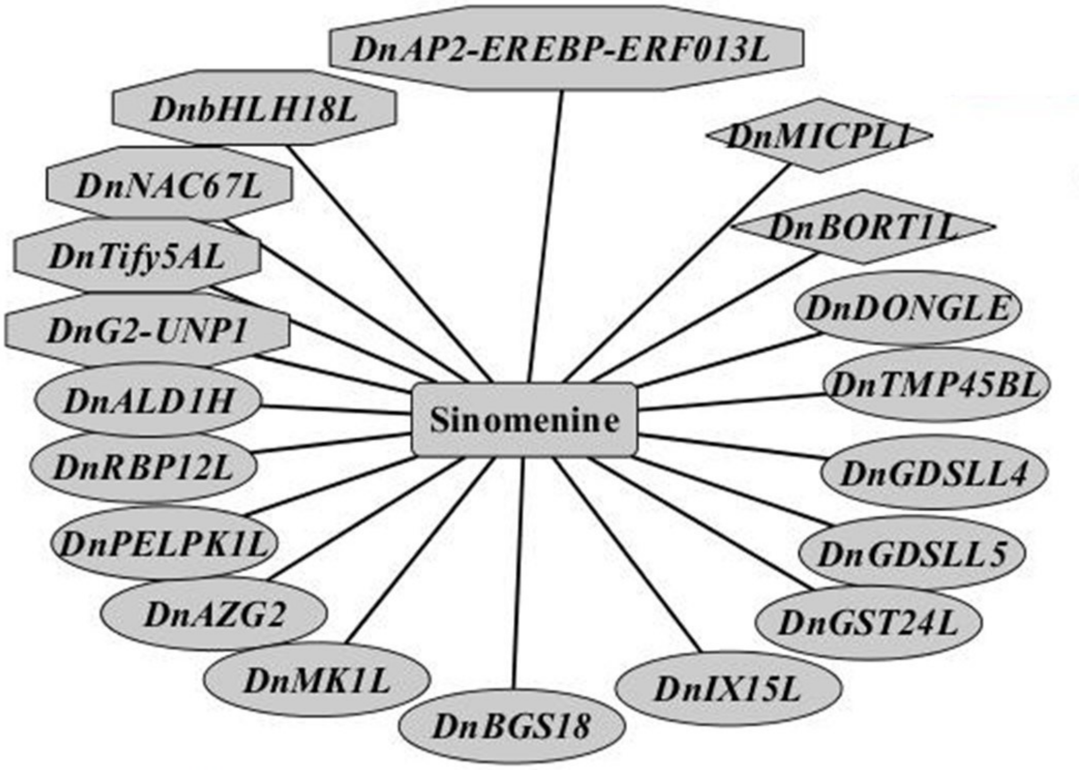

Figure S4. Collinear networks of extremely significantly correlated DAMs and DEGs in roots of *D. nobile*. Square boxes indicate DAMs, ellipse boxes indicate DEGs of metabolic enzyme coding genes, diamond boxes indicate DEGs of transporters, octagon boxes indicate DEGs of TFs. Each link means coefficient > 0.99 and $p < 0.01$.
