# Supplementary material for: Metabolic and Transcriptomic Profile Revealing the Differential Accumulating Mechanism in Different Parts of Dendrobium nobile
Source: Int J Mol Sci. 2024 May 14;25(10):5356. doi: 10.3390/ijms25105356 (PMC11121218; doi:10.3390/ijms25105356)
Supplement: Supplementary file 1 [file ijms-25-05356-s001.zip › Table S1.pdf]

Table S1 Detailed information of differentially accumulated metabolites in different parts of *D. nobile*

Compound_ID	Name	Formula	Molecular Weight	RT (min)	Q1 (m/z)	Categories	Peak areas						Kegg_ID	HMDB_ID	Lipidmaps_ID
							Roots	Stems	Leaves	Flowers	Fruits	QC			
Com_1_neg	Mevalonic acid	C6H12O4	148.157	0.75	147.100	Organic acids	94556.67 ± 23113.14	147166.67 ± 18267.34	132100 ± 21003.97	218230 ± 155374.52	649666.67 ± 13292.69	277400 ± 29596.28	C00418	HMDB0000227	LMFA01050352
Com_10_neg	Lactobionic acid	C12H22O	358.296	0.59	357.102	Saccharides	1264333.33 ± 103824.	1431000 ± 211472.61	3693666.67 ± 205449.	14023333.33 ± 32882	65636666.67 ± 19581	33000000 ± 4762695.32			
Com_106_neg	γ,γ-Dimethylallyl pyrophosphate	C5H12O7	246.092	0.71	244.998	Others	37673.33 ± 10656.23	28233.33 ± 878.04	523566.67 ± 100919.1	117833.33 ± 25900.62	54203.33 ± 9654.13	138266.67 ± 18044.45			
Com_108_neg	Isoschaftoside	C26H28O	564.492	5.43	563.141	Flavonoids	14053.33 ± 1375.12	823833.33 ± 73570.66	2043666.67 ± 143903.	373433.33 ± 346562.9	412466.67 ± 30263.77	707566.67 ± 20542.69			LMPK12110243
Com_110_neg	Isorhamnetin	C16H12O	316.262	10.02	315.051	Flavonoids	8527.33 ± 1735.74	11149 ± 3547.09	3861.33 ± 863.82	16852 ± 11131.09	440533.33 ± 25930.46	79790 ± 92.01	C10084	HMDB0002655	LMPK12110002
Com_113_neg	Isomucronulatol-7-O-glucoside	C23H28O	464.462	6.52	463.161	Flavonoids	12424.33 ± 2776.5	56396.67 ± 3807.01	267133.33 ± 43764.7	26050000 ± 7802465.	3365000 ± 247488.72	5326000 ± 247747.18			
Com_114_neg	Adipic acid	C6H10O4	146.058	0.70	145.058	Organic acids	275166.67 ± 41279.56	188933.33 ± 19432.85	682500 ± 49945.84	1936833.33 ± 101886	306933.33 ± 42444.81	746033.33 ± 16448.17	C06104	HMDB0000448	LMFA01170048
Com_119_neg	alpha-Ketoglutaric acid	C5H6O5	146.098	0.67	145.000	Organic acids	8391 ± 574.35	32250 ± 2052.96	29103.33 ± 5001.35	183053.33 ± 66765.29	25090 ± 2155.8	58060 ± 16172.58	C00026	HMDB0000208	
Com_120_neg	Ribitol	C5H12O5	152.069	0.70	151.000	Saccharides	4987.33 ± 1322.39	134966.67 ± 43376.21	2580000 ± 445135.93	97370 ± 86997.31	30053.33 ± 3473.56	453833.33 ± 89632.89	C00474	HMDB0000508	
Com_121_neg	Allantoin	C4H6N4C	158.115	0.67	157.000	Others	15246.67 ± 3373.67	19520 ± 1924.39	241733.33 ± 7360.4	46013.33 ± 6530.09	19360 ± 1897.38	69506.67 ± 3848.47	C01551	HMDB0000462	
Com_124_neg	Ribose 5-phosphate	C5H11O8	230.110	0.62	229.000	Saccharides	8074.67 ± 3106.84	17925.33 ± 5819.38	5673.67 ± 1740.17	41256.67 ± 8370.33	18863.33 ± 6872.56	28310 ± 3571.68	C00117	HMDB0001548	
Com_125_neg	Lactic acid	C3H6O3	90.078	0.72	89.000	Organic acids	3950 ± 1465.68	3693 ± 2227.27	9253 ± 1217.68	9439.33 ± 8873.39	27843.33 ± 8779.77	5159.67 ± 1722.54	C01432	HMDB0000190	
Com_133_neg	9-HpOTrE	C18H30O	310.100	15.80	309.100	Lipids	8416.67 ± 425.01	8032 ± 272.42	3541.67 ± 1015.04	32453.33 ± 17033.81	4542.67 ± 209.19	12906.67 ± 1278.03			LMFA02000108
Com_14_neg	Kaji-Ichigoside F1	C36H58O	650.840	6.97	649.396	Others	8622.33 ± 2333.26	32362.33 ± 1440.01	11230.33 ± 5788.8	790566.67 ± 167936.5	24352.67 ± 440.55	86010 ± 7052.47			
Com_142_neg	D-Glutamic acid	C5H9NO4	147.129	0.73	148.067	Amino acid and its derivatives	16646.67 ± 2778.52	14996.67 ± 2114.98	37896.67 ± 5000.38	109506.67 ± 43618.76	49206.67 ± 8402.47	39700 ± 4466.86	C00217	HMDB0003339	
Com_146_neg	Myricitrin	C21H20O	464.376	6.86	463.088	Flavonoids	12424.33 ± 2776.5	38174 ± 3146.38	642133.33 ± 82559.24	50090000 ± 14635206	7490333.33 ± 419860.	12353333.33 ± 20417	C10108	HMDB0034360	LMPK12112436
Com_147_neg	3-O-Feruloylquinic acid	C17H20O	368.335	4.71	367.000	Phenols	18070 ± 2622.45	6573.33 ± 291.56	20923.33 ± 2285.06	25452.33 ± 21597.2	114700 ± 11392.1	33056.67 ± 4273.25	C02572	HMDB0030669	
Com_148_neg	Isotrifoliin	C21H20O	464.096	7.40	463.000	Flavonoids	12424.33 ± 2776.5	56396.67 ± 3807.01	767500 ± 32846.41	22920000 ± 5050036.	3355333.33 ± 412569.	5773333.33 ± 131936	C05623	HMDB0037362	
Com_15_neg	Kinsenoside	C10H16O	264.229	0.89	309.083	Others	292766.67 ± 43866.03	292766.67 ± 10908.81	273700 ± 20881.09	295733.33 ± 35680.28	816733.33 ± 73104.6	500900 ± 19380.57			
Com_150_neg	Poncirin	C28H34O	594.195	8.40	593.000	Flavonoids	12886.67 ± 1851.44	171482 ± 8133.15	9763 ± 815.36	1103700 ± 567389.85	2383000 ± 150202.09	696766.67 ± 36141.42	C09830	HMDB0037487	LMPK12140333
Com_153_neg	Saponarin	C27H30O	594.518	7.81	593.160	Flavonoids	6406 ± 1563.68	105943.33 ± 8651.73	1862666.67 ± 112772.	440356.67 ± 273175.6	281100 ± 9974.3	576466.67 ± 11621.91	C08064		LMPK12110292
Com_154_neg	D-Malic acid	C4H6O5	134.087	0.63	133.014	Organic acids	25250000 ± 1475149.	14613333.33 ± 20487	39076666.67 ± 29626	32966666.67 ± 14878	73993333.33 ± 51480	39876666.67 ± 56794	C00497	HMDB0031518	
Com_157_neg	Kaempferol 3-O-robinobioside (Biorobin)	C27H30O	594.518	8.46	593.159	Flavonoids	11871.33 ± 2218.62	121531.67 ± 9102.86	10577 ± 1882.98	1215800 ± 645854.29	2758000 ± 177484.27	778000 ± 50612.51			
Com_158_neg	D-Glucose 6-phosphate	C6H13O9	260.136	0.63	259.000	Saccharides	225666.67 ± 12737.43	394833.33 ± 27368.64	271933.33 ± 16221.45	1530733.33 ± 734385.	464966.67 ± 17924.72	499366.67 ± 30410.12	C00092	HMDB0001401	
Com_16_neg	Neoeriocitrin	C27H32O	596.534	8.31	595.167	Flavonoids	1796.33 ± 622.77	2793.67 ± 468.37	9532.67 ± 998.56	24540 ± 9314.19	52203.33 ± 2793.45	17360 ± 2644.78	C09805		LMPK12140359
Com_163_neg	Lonicerin	C27H30O	594.518	7.67	593.151	Flavonoids	30713.33 ± 3331.41	44038.33 ± 4223.55	15636.67 ± 3344.61	5964666.67 ± 121640	866866.67 ± 3300.84	1472000 ± 31790.98	C17557	HMDB0005799	
Com_169_neg	Tiliroside	C30H26O	594.520	7.69	593.130	Flavonoids	25010 ± 2751.74	44038.33 ± 4223.55	14556.67 ± 1038.25	6569000 ± 1033790.4	837733.33 ± 16232.14	1315000 ± 13366.63			LMPK12111791
Com_17_neg	Vanillic acid	C8H8O4	168.147	4.18	167.035	Phenols	17710 ± 1632.75	3087.33 ± 949.47	5785 ± 1781.62	310100 ± 247264.49	20781.33 ± 647.24	53733.33 ± 4630.7	C06672	HMDB0000484	
Com_172_neg	O-Phosphorylethanolamine	C2H8NO4	141.063	0.65	140.000	Amino acid and its derivatives	4923 ± 777.21	4671 ± 1645.42	20316.67 ± 6109.35	124543.33 ± 62509.26	52243.33 ± 14024.89	37760 ± 1194.68	C00346	HMDB0000224	
Com_18_neg	Apigenin-6,8-di-C-glycoside	C27H30O	594.518	4.83	593.151	Flavonoids	111486.67 ± 18573.72	14256666.67 ± 92924	86293333.33 ± 39838	8194000 ± 9375081.9	3593000 ± 195245.15	31163333.33 ± 64711	C10195		
Com_184_neg	Cyanidin 3-O-glucoside	C21H21O	448.300	8.33	447.300	Flavonoids	2443.67 ± 375.27	9189.67 ± 2411.27	88250 ± 16924.88	1330533.33 ± 816702.	84276.67 ± 3138.85	296300 ± 9267.51	C08604	HMDB0030684	LMPK12010110
Com_19_neg	Naringenin	C15H12O	272.253	8.24	271.000	Flavonoids	2992 ± 496.47	16000 ± 2345.44	15500 ± 1324.11	16563.33 ± 4865.62	41603.33 ± 3049.56	12936.67 ± 3393.47	C00509	HMDB0002670	LMPK12140001
Com_191_neg	Heptamethoxyflavone	C22H24O	432.420	5.00	431.100	Flavonoids	3751.33 ± 2291.07	24590 ± 2325.12	3110 ± 457.99	12100 ± 9444.54	5887.33 ± 1696.22	6464 ± 1559.71			
Com_197_neg	Rutin	C27H30O	610.518	6.66	609.100	Flavonoids	12424.33 ± 2776.5	38174 ± 3146.38	278033.33 ± 47049.64	87220000 ± 24405823	3150666.67 ± 76560.8	19640000 ± 377977.0	C05625	HMDB0003249	LMPK12112098
Com_2_neg	1,6-Anhydro-β-D-Glucopyranose	C6H10O5	162.141	0.73	161.046	Saccharides	213566.67 ± 27412.2	494233.33 ± 18222.39	324033.33 ± 32787.23	1077366.67 ± 477452.	538100 ± 3550.59	523100 ± 57302.88			
Com_200_neg	3-(2-Naphthyl)-D-alanine	C13H13N	215.100	5.90	214.100	Amino acid and its derivatives	3486.33 ± 1082.19	2246.33 ± 504.22	7488.33 ± 258.42	10718.33 ± 8959.06	28783.33 ± 6250.12	9153.33 ± 3993.15			

Com_202_neg	Dihydrodehydrodiconiferyl Alcohol	C20H24O	360.401	14.14	359.150	Phenols	37550 ± 6209.77	18720 ± 1151.81	2247.33 ± 367.31	9230 ± 7776.52	3995.67 ± 1258.56	12236.67 ± 1038.47			
Com_204_neg	Sinomenine	C19H23N	329.390	10.26	328.155	Alkaloids	38920 ± 6200.68	6750.67 ± 1199.62	11570 ± 1788.65	5878 ± 1262.74	5360 ± 431.74	11279.33 ± 2237.78	C09643		
Com_205_neg	Quercetin 3-β-D-glucoside	C21H20O	464.376	6.78	463.087	Saccharides	12424.33 ± 2776.5	40940.67 ± 2663.21	1080433.33 ± 156881.	99023333.33 ± 26244	14170000 ± 445944.6	21953333.33 ± 1376041.02			
Com_206_neg	Quercetin-3'-O-glucoside	C21H20O	464.376	6.51	463.088	Flavonoids	12424.33 ± 2776.5	40940.67 ± 2663.21	911000 ± 146810.44	83210000 ± 18146086	11515000 ± 1216285.	16746666.67 ± 3430076.12			
Com_209_neg	2-Hydroxybutanoic acid	C4H8O3	104.104	0.62	103.000	Organic acids	5453 ± 2002.6	5189.33 ± 2175.19	12308.33 ± 2300.24	11818.33 ± 2141.32	23216.67 ± 2822.11	11038.33 ± 1627.45	C05984	HMDB0000008	LMFA01050342
Com_212_neg	Kaempferide	C16H12O	300.263	12.80	299.000	Flavonoids	285066.67 ± 50157.77	219633.33 ± 15104.6	114700 ± 11706.41	40949 ± 44173.72	10956666.67 ± 18263	999633.33 ± 31141.65	C10098	HMDB0037441	LMPK12110563
Com_214_neg	Isovitexin	C21H20O	432.378	6.93	431.100	Flavonoids	20996.67 ± 3525.5	897666.67 ± 47908.27	62980000 ± 5676636.	25493666.67 ± 25194	2907333.33 ± 162366.	15290000 ± 207042.6	C01714		LMPK12110338
Com_215_neg	JA-Ile			11.79	322.100	Lipids	254700 ± 26987.78	13263.33 ± 819.16	622233.33 ± 52971.08	218566.67 ± 104822.2	156200 ± 4432.46	254500 ± 11990.83			
Com_216_neg	Sibirioside A	C21H28O	472.440	9.16	471.151	Others	3989.67 ± 983.35	51016.67 ± 5650.93	31283.33 ± 3997.47	442966.67 ± 194502.0	15543.33 ± 1782.44	113566.67 ± 6593.09			
Com_219_neg	Morin hydrate	C15H12O	320.251	7.25	301.035	Flavonoids	8539.33 ± 1785.13	30302.33 ± 1382.65	2908.67 ± 1123.12	211366.67 ± 65269.56	28083.33 ± 2896.45	37680 ± 2257.85		HMDB0030796	
Com_22_neg	Rhamnetin	C16H12O	316.262	1.60	315.000	Flavonoids	24456.67 ± 4600.59	29290 ± 1870.83	80763.33 ± 21199.92	2752000 ± 944294.8	881333.33 ± 55874.7	1153666.67 ± 47618.8	C10176		LMPK12112624
Com_220_neg	β-D-glucopyranosyl-caffeic acid			0.63	341.100	Phenols	69310 ± 9422.2	129200 ± 9863.06	56416.67 ± 7289.97	555366.67 ± 262019.3	113570 ± 14180.85	211000 ± 45243.56			
Com_221_neg	Apigenin 4-O-rhamnoside	C21H20O	416.378	9.91	415.100	Flavonoids	7876.33 ± 1525.21	11275.67 ± 2309.75	303600 ± 32282.5	12669.33 ± 7330.6	14850 ± 2930.27	64910 ± 11170.73			
Com_222_neg	Apigenin	C15H10O	270.237	9.82	269.000	Flavonoids	6453.67 ± 1583.36	3042 ± 603.38	63406.67 ± 3553.71	18887 ± 1794.27	16594.67 ± 1109.63	12576.67 ± 1578.7	C01477	HMDB0002124	LMPK12110005
Com_23_neg	Pinoresinol-glucoside	C26H32O	520.526	9.17	519.187	Phenols	7550.33 ± 545.53	2726.33 ± 646.53	2601.33 ± 844.99	11990.67 ± 4493.73	26260 ± 1543.78	11826.67 ± 1339.56			
Com_230_neg	D-Erythrose 4-phosphate	C4H9O7P	200.084	0.61	199.000	Saccharides	11924.67 ± 4385.37	22580 ± 3177.18	21880 ± 2940.08	42263.33 ± 16211.95	82736.67 ± 6483.92	49400 ± 8210.66	C00279	HMDB0001321	
Com_231_neg	deoxyribose-phosphate			0.65	213.000	Saccharides	6553 ± 415.18	5069 ± 1288.63	35220 ± 11892.03	6300.33 ± 2743.38	3874 ± 1863.97	16803.33 ± 2615.62			
Com_233_neg	8-Gingerol	C43H32O	868.702	0.84	321.207	Phenols	12542 ± 2363.48	16443.33 ± 3270.07	99650 ± 6427.42	278063.33 ± 127759.5	41523.33 ± 4180.03	143233.33 ± 7487.03			
Com_235_neg	Caffeic acid O-glucoside	C15H18O	342.000	1.40	341.000	Phenols	31576.67 ± 10927.46	28680 ± 2789.42	232433.33 ± 16663.2	11926333.33 ± 32863	1658333.33 ± 249124.	2724666.67 ± 145945.96			
Com_25_neg	Inositol	C6H12O6	180.156	0.72	179.157	Saccharides	1245333.33 ± 91914.2	1269333.33 ± 21060.7	657800 ± 26265.69	1479566.67 ± 777765.	4444333.33 ± 479245.	2262333.33 ± 20336.0	C00137	HMDB0000211	
Com_250_neg	Docetaxel	C43H53N	807.879	0.69	852.345	Terpenoids	4104.33 ± 360.55	14432.67 ± 4364.02	5541 ± 1969.65	5217 ± 1316.27	2241.67 ± 202.85	3634 ± 1109.68	C11231	HMDB0015378	
Com_251_neg	Rhoifolin	C27H30O	578.519	7.78	577.156	Flavonoids	56456.67 ± 2947.39	4324.67 ± 1996.46	31343.33 ± 2541.51	10055.33 ± 4699.15	189833.33 ± 5551.18	60243.33 ± 4294.84	C12627	HMDB0038848	
Com_255_neg	Trifolin	C21H20O	448.101	8.32	447.000	Flavonoids	4893.67 ± 1326.42	27276.67 ± 3296.1	208333.33 ± 11247.02	2695666.67 ± 123876	178833.33 ± 3092.28	713800 ± 32021.24	C12626	HMDB0030864	LMPK12111663
Com_257_neg	Pyruvic Acid	C3H4O3	88.062	0.59	87.009	Organic acids	503866.67 ± 159353.2	2526000 ± 20510.16	1073066.67 ± 146770.	2264333.33 ± 750924.	5108666.67 ± 377067.	2422000 ± 533432.28	C00022	HMDB0000243	LMFA01060077
Com_258_neg	Glucosylvitexin	C27H30O	594.518	4.84	593.151	Flavonoids	9077 ± 483.98	886833.33 ± 66870.14	7348333.33 ± 287893.	571160 ± 665524.8	219033.33 ± 9309.61	2090000 ± 88196.75			
Com_261_neg	Trifolirhizin +HCOOH	C22H22O	446.404	18.58	491.120	Flavonoids	21320 ± 9588.96	165666.67 ± 12719.1	77200 ± 9923.92	47233.33 ± 40586.58	46460 ± 8685.91	48213.33 ± 3165.51			
Com_264_neg	Hesperidin	C28H34O	610.190	7.50	609.190	Flavonoids	12424.33 ± 2776.5	38174 ± 3146.38	17053.33 ± 3811.13	2449333.33 ± 105132	136900 ± 14827	493600 ± 22926.11	C09755	HMDB0003265	LMPK12140451
Com_267_neg	Quercetin 4'-O-glucoside (Spiraeoside)	C21H20O	464.096	7.40	463.000	Flavonoids	12424.33 ± 2776.5	56396.67 ± 3807.01	701500 ± 37934.94	24170000 ± 7614319.	3421000 ± 129493.89	5706333.33 ± 415532.06			
Com_269_neg	2-Oxoadipic acid	C6H8O5	160.037	0.78	159.100	Organic acids	48013.33 ± 3815.14	48446.67 ± 3854.21	74773.33 ± 7320.33	100560 ± 14882.27	194966.67 ± 10984.03	96623.33 ± 4048.71	C00322	HMDB0000225	
Com_270_neg	Sinigrin	C10H16K	397.460	0.63	358.000	Others	18590 ± 6851.09	38620 ± 2162.22	32050 ± 1429.2	165266.67 ± 40834	533466.67 ± 31740.23	273533.33 ± 17915.05	C08427	HMDB0034070	
Com_272_neg	Syringic acid O-glucoside		360.100	1.10	359.100	Phenols	43306.67 ± 8985.11	109756.67 ± 40760.5	30460 ± 2657.45	466766.67 ± 215191.3	2871666.67 ± 451343.	689133.33 ± 59348.48			
Com_275_neg	Salicylic acid O-glucoside	C13H16O	300.100	2.66	299.100	Phenols	73650 ± 11502.1	54536.67 ± 1681.43	753366.67 ± 95359.4	2593333.33 ± 640425.	12090000 ± 759254.0	3406333.33 ± 101948.79			
Com_276_neg	Columbin	C20H22O	358.385	10.83	313.217	Terpenoids	1011.67 ± 159.31	563.67 ± 78.96	581.33 ± 247.94	4782.33 ± 3020.7	10799 ± 611.35	2101 ± 313.48	C09077	HMDB0036837	
Com_279_neg	Crocetin	C20H24O	328.402	10.65	327.160	Terpenoids	44910 ± 4576.67	90273.33 ± 6883	219466.67 ± 16994.18	51905.33 ± 59171.54	38716.67 ± 2498.66	87720 ± 2649.31	C08588		LMPR0104010020
Com_28_neg	D-Glucose	C6H12O6	180.156	0.79	179.056	Saccharides	537433.33 ± 92116.82	464266.67 ± 17434.51	248966.67 ± 24997.78	936000 ± 55851.23	1818000 ± 52561.08	948566.67 ± 14442.84	C00031	HMDB0000516	
Com_282_neg	Glycitin	C22H22O	446.404	8.24	445.100	Flavonoids	11534.67 ± 737.41	429000 ± 35004.67	10901 ± 6853.44	45381 ± 23485.38	32666.67 ± 718.16	103423.33 ± 3393.78	C16195	HMDB0002219	
Com_283_neg	Guanidinoethyl Sulfonate	C3H9N3C	167.187	0.72	166.030	Amino acid and its derivatives	5625.67 ± 1967.35	8818 ± 2458.98	172870 ± 64909.8	54835 ± 67009.17	13098.33 ± 4501.57	61946.67 ± 11398.42		HMDB0003584	
Com_285_neg	Sibiricose A6	C23H32O	548.491	6.29	547.167	Phenols	6171.33 ± 1132.86	6160.67 ± 2622.38	21957.33 ± 1544.66	58266.67 ± 14209.58	13290 ± 678.58	15013.33 ± 1553.76			
Com_287_neg	L-Tyrosine	C9H11NC	181.190	1.31	180.000	Amino acid and its derivatives	48553.33 ± 17940.02	22706.67 ± 3986.52	261700 ± 69298.82	1704333.33 ± 422904.	253466.67 ± 94779.62	304866.67 ± 48536.74	C00082	HMDB0000158	
Com_288_neg	Succinic anhydride	C4H4O3	100.073	0.63	99.016	Organic acids	139303.33 ± 63696.59	64203.33 ± 5132.81	37266.67 ± 9893.12	119606.67 ± 65986.78	368066.67 ± 70290.84	155300 ± 26254.65	C19524	HMDB0032523	
Com_292_neg	3-Aminosalicylic Acid	C7H7NO3	153.135	3.49	152.000	Phenols	19886.67 ± 2162.55	56210 ± 16649.43	251766.67 ± 23651.69	115668.33 ± 9268.43	142016.67 ± 13346.72	87710 ± 24123.48			
Com_296_neg	Eriodictyol	C15H12O	288.252	7.73	287.056	Flavonoids	13332.33 ± 3054.12	18560 ± 1123.3	35360 ± 8614.05	22209.67 ± 6434.96	85663.33 ± 5774.69	29020 ± 2736.47	C05631	HMDB0005810	LMPK12140002

Com_299_neg	N-Acetyl-D-Glucosamine	C8H15NC	221.208	0.78	220.083	Saccharides	10607 ± 1020.71	16303.33 ± 1894.3	30773.33 ± 1471.97	62748.67 ± 5511.55	6316.67 ± 3345.04	18833.33 ± 4645.4		HMDB0000215	
Com_302_neg	Homocysteine	C4H9NO2	134.185	0.70	133.900	Amino acid and its derivatives	4736.67 ± 1707.06	3437 ± 2058.39	20560 ± 316.33	52199.33 ± 34434.25	206833.33 ± 88031.9	20023.33 ± 6007.74	C05330	HMDB0000742	
Com_306_neg	12OHJA-Ile-1			8.79	338.000	Lipids	6824.33 ± 676.52	5477.33 ± 1324.07	80300 ± 7622.69	2210.67 ± 1282.34	22963.33 ± 744.51	23443.33 ± 3740.32			
Com_308_neg	1-O-b-D-glucopyranosyl sinapate	C17H22O	386.351	6.55	385.100	Phenols	22389.67 ± 7799.83	8885.67 ± 381.34	3559.67 ± 1511.93	9317 ± 8113.08	103863.33 ± 4009.24	19953.33 ± 1425.96			
Com_31_neg	Luteolin 7-O-glucoside	C21H20O	448.377	8.51	447.092	Flavonoids	8547.33 ± 1853.52	38720 ± 1032.89	8954.33 ± 3503.63	159800 ± 6672.83	20923.33 ± 3108.1	43870 ± 1768.86	C03951	HMDB0035588	
Com_313_neg	Astragalin	C21H20O	448.377	7.54	447.100	Flavonoids	6858.33 ± 3595.43	6143.67 ± 1020.72	570533.33 ± 71635.02	29832333.33 ± 220432	638733.33 ± 20941.24	6826333.33 ± 200792.	C12249	HMDB0037429	LMPK12111725
Com_317_neg	Tricin O-saccharic acid		522.100	8.77	521.100	Flavonoids	126950 ± 30370.57	12433.33 ± 2193.34	87803.33 ± 3770.56	249330 ± 211032.22	1271000 ± 10424.33	385566.67 ± 29460.29			
Com_326_pos	2,6-Diaminooimelic Acid	C7H14N2	190.197	0.70	191.100	Organic acids	774966.67 ± 46787.77	540733.33 ± 24672.03	2129666.67 ± 123097.	623733.33 ± 111246.5	160600 ± 1877.94	703233.33 ± 27066.63			
Com_33_neg	Kynurenic acid	C10H7NC	189.167	4.52	188.043	Amino acid and its derivatives	9327 ± 2203.2	6901.33 ± 930.4	132063.33 ± 67386.88	48061 ± 52552.13	10643.33 ± 1129.97	47763.33 ± 14589.48	C01717	HMDB0000715	
Com_335_pos	trans-3-Hydroxy-L-proline	C5H9NO3	131.130	0.81	132.062	Amino acid and its derivatives	78160 ± 18565.98	201766.67 ± 10207.95	374800 ± 35947.83	809900 ± 215833.28	70400 ± 14476.84	370733.33 ± 3013.67	C05147		
Com_337_pos	Tricetin O-hexoside			6.52	465.000	Flavonoids	63854.33 ± 7833	56650 ± 7818.88	78586.67 ± 2502.88	2411666.67 ± 1240372	305800 ± 42025.07	497600 ± 29086.42			
Com_344_pos	Parthenolide	C15H20O	248.317	6.81	249.149	Terpenoids	490557.67 ± 41903.1	430200 ± 27111.74	1954000 ± 283309.02	621000 ± 532821.87	310033.33 ± 29132.49	522233.33 ± 12877.46	C07609		LMPR0103090002
Com_346_pos	Panaxydol	C17H24O	260.371	17.44	261.185	Lipids	18956.67 ± 6574.78	36683.33 ± 959.87	69963.33 ± 15719.26	44676.67 ± 21857.71	138466.67 ± 10664.69	54870 ± 5638.94		HMDB0034000	LMFA05000028
Com_347_pos	Coniferyl aldehyde	C10H10O	178.185	14.95	179.100	Phenols	155733.33 ± 7926.89	146200 ± 2616.61	286533.33 ± 31394.3	69870 ± 51815.46	37393.33 ± 7910.32	106250 ± 6773.6	C02666		
Com_348_pos	COLUMBIANETIN ACETATE	C16H16O	288.295	12.56	289.107	Phenols	15370 ± 3220.53	19023.33 ± 1171.45	18920 ± 2534.4	31108.67 ± 4968.18	78200 ± 8158.64	21333.33 ± 2755.7			
Com_349_pos	Linthospermic Acid +Na	C27H22O	538.456	6.98	561.101	Phenols	20248.67 ± 958.73	19008.67 ± 1752.2	16703.33 ± 666.2	17976.33 ± 6566.71	60010 ± 7459.71	22956.67 ± 4504.5			
Com_351_pos	L-Leucine	C6H13NC	131.173	1.23	132.100	Amino acid and its derivatives	32943333.33 ± 234825	50336666.67 ± 108730	102523333.33 ± 74524	280166666.67 ± 86657	59263333.33 ± 297640	96386666.67 ± 329070	C00123	HMDB0000687	
Com_352_pos	L-Isoleucine	C6H13NC	131.173	1.23	132.000	Amino acid and its derivatives	23090000 ± 1731896.8	34993333.33 ± 684218	69316666.67 ± 622174	191666666.67 ± 56612	41360000 ± 2153740.9	68650000 ± 4360512.2	C00407	HMDB0000172	
Com_354_pos	Kaempferol7-O-β-D-glucopyranoside	C21H20O	448.377	5.49	449.108	Flavonoids	11300 ± 1536.51	16639.67 ± 1071.54	24036.67 ± 5874.73	1205766.67 ± 368747.	249733.33 ± 9398.35	261633.33 ± 19471.23			
Com_355_pos	Kaempferol 3-O-glucoside-2'' -O-rhamnoside			5.29	595.300	Flavonoids	6233.33 ± 2395.73	68770 ± 3467.49	20746.33 ± 3244.83	1066166.67 ± 402402.	139600 ± 20035.14	194066.67 ± 10725.15			
Com_358_pos	Denin	C33H56O	676.790	13.46	694.400	Terpenoids	4313.67 ± 2623.1	3304 ± 1120.18	203300 ± 15024.2	10378 ± 4971.27	65226.67 ± 4839.12	53506.67 ± 3609.2			
Com_362_pos	di-C,C-hexosyl-apigenin	C27H30O	594.518	4.28	595.170	Flavonoids	27923.33 ± 4177.02	3013000 ± 64915.33	21843333.33 ± 214912	2401000 ± 2812569.6	758566.67 ± 25040.81	5284666.67 ± 318790.77			
Com_365_pos	Phenylacetylglutamine	C13H16N	264.277	1.03	265.100	Amino acid and its derivatives	158766.67 ± 17859.51	103036.67 ± 8082.74	340833.33 ± 22512.27	1879000 ± 565905.17	82790 ± 11259.89	485900 ± 26406.56	C04148	HMDB0006344	
Com_367_pos	Caffeic Acid	C9H8O4	180.157	3.08	181.049	Phenols	42241.67 ± 4584.23	304256.67 ± 5915.72	102002.67 ± 6036.52	48503333.33 ± 259559	86680 ± 20773.04	12206666.67 ± 310948	C01481	HMDB0001964	
Com_369_pos	N1-Acetylspermine	C12H28N	244.377	1.13	245.200	Amino acid and its derivatives	25950 ± 3574.6	55836.67 ± 963.75	101886.67 ± 13171.35	42763.33 ± 13238.85	31886.67 ± 8514.31	59600 ± 7512.15	C02567	HMDB0001186	
Com_370_pos	N6-Acetyl-L-Lysine	C8H16N2	188.224	0.76	189.123	Amino acid and its derivatives	141333.33 ± 11387.81	641600 ± 70581.91	1162666.67 ± 68878.3	541166.67 ± 135331.7	127466.67 ± 7960.88	408633.33 ± 47688.74		HMDB0000206	
Com_371_pos	N2-Methylguanosine	C11H15N	297.267	1.07	298.200	Nucleotide and its derivates	41203.33 ± 8238.21	62110 ± 14368.31	47416.67 ± 10607.05	142966.67 ± 26329.49	40816.67 ± 1399.87	51716.67 ± 19561.84			
Com_384_pos	Prim-O-glucosylcimifugin	C22H28O	468.451	5.19	469.170	Flavonoids	31536.67 ± 8010.71	2536000 ± 65238.03	240366.67 ± 27001.4	183620 ± 172793.01	624166.67 ± 62392.81	1061833.33 ± 106936.38			
Com_385_pos	PolygalaxanthoneⅥ	C23H26O	494.440	6.45	495.150	Phenols	9741.67 ± 1284.36	10739.33 ± 7306.42	23666.67 ± 1713.13	72070 ± 19511.81	24970 ± 1819.95	18396.67 ± 1827.83			
Com_389_pos	1-Linoleoyl-Rac-Glycerol	C21H38O	354.524	16.67	355.283	Lipids	2737 ± 1024.84	2288.67 ± 504.51	1734.67 ± 742.25	12856.67 ± 2231.67	30743.33 ± 2976.4	5388.33 ± 331.7			

Com_40_neg	D-3-Phosphoglyceric acid	C3H5H2C	230.021	0.50	184.986	Lipids	72666.67 ± 2960.42	77173.33 ± 1363.73	497200 ± 59983.5	58880 ± 2548.82	91710 ± 6654.63	137766.67 ± 16738.45			
Com_400_pos	Spermidine	C7H19N3	145.246	0.50	146.200	Amino acid and its derivatives	122545 ± 10499.33	69086.67 ± 23075.17	304000 ± 19695.85	166066.67 ± 10325.48	725133.33 ± 67519.4	182333.33 ± 26850.74	C00315	HMDB0001257	
Com_401_pos	(+)-Tetrandrine	C38H42N	622.750	5.53	623.312	Alkaloids	101720 ± 28697.53	29376.67 ± 3925.45	59400 ± 3029.6	28875 ± 23829.28	260466.67 ± 16711.74	93363.33 ± 33541.24	C09654		
Com_402_pos	Stachydrine	C7H13NC	159.183	0.78	144.102	Alkaloids	640633.33 ± 110173.6	1391666.67 ± 3299.83	2557333.33 ± 244671.	1022500 ± 736998.32	1241000 ± 177318.92	1457000 ± 97703.63	C10172	HMDB0004827	
Com_403_pos	(-)-Norepinephrine N-hexoside	C14H21N	331.320	0.77	332.130	Phenols	1443666.67 ± 190510.	798466.67 ± 11858.71	1066000 ± 34477.05	1873333.33 ± 629115.	5505333.33 ± 255825.	2099000 ± 62886.14			
Com_415_pos	D-Glucosamine	C6H13NC	179.171	0.63	180.000	Saccharides	609733.33 ± 13121.57	2379666.67 ± 50307.9	899666.67 ± 46658.28	671333.33 ± 719582.3	313100 ± 48376.03	672466.67 ± 145682.2	C00329	HMDB0001514	
Com_419_pos	methylApigenin C-hexoside	C22H22O	446.404	6.52	447.130	Flavonoids	21923 ± 2262.5	5243 ± 813.73	129100 ± 2006.66	16983.67 ± 15896.76	49655.33 ± 2031.44	20876.67 ± 6726.54			
Com_421_pos	5-Methoxyindoleacetic acid	C11H11N	205.210	2.55	206.000	Organic acids	91896.67 ± 7472.72	93750 ± 6474.82	4426666.67 ± 453502.	1035866.67 ± 464634.	337900 ± 30245.99	910000 ± 35572.74	C05660	HMDB0004096	
Com_43_neg	2-Methylglutaric Acid	C6H10O4	146.141	0.64	145.100	Organic acids	276933.33 ± 20221.5	168633.33 ± 11095.44	574900 ± 27634.88	1571166.67 ± 783083.	289266.67 ± 40588.12	607233.33 ± 63369.88		HMDB0000422	
Com_432_pos	Oleic acid	C18H34O	282.461	18.28	283.265	Lipids	10535.33 ± 4262.34	21510 ± 1331.04	12882.67 ± 2712.38	20903.33 ± 6186.63	52346.67 ± 6674.85	19336.67 ± 1137.32	C00712	HMDB0000207	LMFA01030002
Com_434_pos	Nα-Acetyl-L-Arginine	C8H16N4	216.238	0.81	217.100	Amino acid and its derivatives	176233.33 ± 16566.3	7366666.67 ± 288820.	770866.67 ± 38443.58	149303.33 ± 108586.1	349700 ± 21882.57	1379000 ± 116801.83			
Com_438_pos	3-Chloro-L-Tyrosine	C9H10ClN	215.634	4.35	216.000	Amino acid and its derivatives	47090 ± 4855.05	122400 ± 1019.8	308866.67 ± 22205.76	121200 ± 99893.4	50990 ± 12009.72	105516.67 ± 17519.62		HMDB0001885	
Com_439_pos	3',4',5'-Tricetin 5-O-hexoside	C24H26O	506.460	6.51	507.150	Flavonoids	138414 ± 19571.79	44603.33 ± 4302.81	36396.67 ± 9629.15	53470 ± 51426.73	269033.33 ± 3922.87	71393.33 ± 9366.94			
Com_443_pos	Nicotinuric Acid	C8H8N2C	180.161	9.67	181.100	Terpenoids	929233.33 ± 126887.5	975200 ± 53317.41	2074000 ± 185743.55	314536.67 ± 400215.4	301266.67 ± 22263.62	743900 ± 50116.73	C05380	HMDB0003269	
Com_444_pos	Neocryptotanshinone	C19H22O	314.375	13.23	315.200	Phenols	35903.33 ± 6642.8	28596.67 ± 6949.7	32500 ± 1401.21	143673.33 ± 101560.7	35013.33 ± 6318.68	44920 ± 5412.49			
Com_447_pos	Apo-13-zeaxanthinone	C18H26O	274.400	14.54	275.200	Terpenoids	24580 ± 4529.25	10103 ± 2818.67	95700 ± 13714.38	26844.33 ± 17379.14	14129.33 ± 3187.28	23523.33 ± 3281.84			
Com_45_neg	2-Isopropylmalic acid	C7H12O5	176.167	0.66	157.052	Organic acids	365366.67 ± 41612.85	317866.67 ± 16442.29	1250333.33 ± 116110.	548866.67 ± 195471.4	182333.33 ± 8230.57	561166.67 ± 55163.35	C02504	HMDB0000402	
Com_454_pos	Nicotinate ribonucleoside	C11H13N	255.224	0.76	256.200	Terpenoids	181266.67 ± 23920.61	83760 ± 4847.27	77556.67 ± 1587.39	398233.33 ± 182859.0	192233.33 ± 19035.46	168000 ± 27286.26		HMDB0006809	
Com_459_pos	Urea	CH4N2O	60.055	0.63	61.000	Others	110823.33 ± 21299.09	67206.67 ± 16707.66	207066.67 ± 18822.74	40550 ± 14495.69	47710 ± 4407.59	72143.33 ± 1349.92	C00086	HMDB0000294	
Com_46_neg	Methylmalonic Acid	C4H6O4	118.088	0.56	117.000	Organic acids	55348 ± 56340.13	25146.67 ± 6250.6	37053.33 ± 8539.8	172026.67 ± 168795.9	437900 ± 79406.93	74986.67 ± 20583.58	C02170	HMDB0000202	LMFA01170118
Com_461_pos	Kaempferitrin	C27H30O	578.519	5.84	579.171	Flavonoids	158494.33 ± 19727.22	76613.33 ± 6798.56	293433.33 ± 36097.49	26885.67 ± 16303.17	42553.33 ± 6731.7	77236.67 ± 8799.41	C16981	HMDB0037438	LMPK12111865
Com_463_pos	Oroxin B	C27H30O	594.518	4.44	595.166	Flavonoids	48060 ± 8734.81	354300 ± 45297.76	2846000 ± 264258.71	306770 ± 336432.42	376833.33 ± 32906.16	690533.33 ± 42922.44			
Com_466_pos	γ-Glu-Leu	C11H20N	260.287	2.91	261.146	Amino acid and its derivatives	155000 ± 24984.13	47740 ± 3419.37	1304333.33 ± 150000.	238500 ± 99745.91	24136.67 ± 1948.1	283266.67 ± 12192.17			
Com_473_pos	LPC(1-acyl 16:0)	C24H50N	495.630	15.06	496.340	Lipids	169133.33 ± 71303.87	157766.67 ± 13658.78	8905.33 ± 550.57	397966.67 ± 189276.5	1281333.33 ± 67277.2	66770 ± 6435.46			
Com_474_pos	LPC(1-acyl 18:1)	C26H52N	521.670	15.29	522.360	Lipids	199733.33 ± 78547.03	1058666.67 ± 35036.4	37920 ± 20221.95	37310 ± 11988.87	88910 ± 25514.01	128466.67 ± 7895.29			
Com_478_pos	Syringetin 3-O-hexoside			3.30	509.100	Flavonoids	21317.33 ± 3204.48	7039.67 ± 1340.05	271000 ± 21761.89	54891.67 ± 23298.42	18756.67 ± 5226.4	47073.33 ± 9042.04			
Com_482_pos	N-Coumaroyltyramine	C17H17N	283.322	7.05	284.000	Phenols	9798000 ± 520151.9	2550000 ± 180149.94	2564666.67 ± 158409.	4128400 ± 4038223.1	27036666.67 ± 74010[illegible]	8951666.67 ± 220256.42			
Com_486_pos	Pteroside A	C21H30O	410.458	4.53	411.201	Terpenoids	79976.33 ± 10658.61	25733.33 ± 7627.4	18136.67 ± 3982.41	113244.67 ± 44893.21	418033.33 ± 16742.03	90823.33 ± 5783.57		HMDB0030758	
Com_493_pos	Polygalaxanthone VII	C27H32O	612.541	5.26	613.176	Phenols	61262.33 ± 9793.79	72352.33 ± 3556.64	21485.67 ± 13516.82	845066.67 ± 298771.9	25220 ± 9279.18	158466.67 ± 46638.78			
Com_495_pos	Biliverdin	C33H34N	582.646	11.00	583.254	Others	10065.33 ± 2795.68	2585.67 ± 1521.71	55860 ± 3272.75	22779.67 ± 16039.94	20616.67 ± 3711	15293.33 ± 3511.94	C00500	HMDB0001008	
Com_496_pos	Phellodenol H	C17H20O	368.335	4.13	369.120	Phenols	115820.67 ± 16666.11	35041.33 ± 1989.16	68430 ± 12639.85	67534.67 ± 40699.93	1248666.67 ± 34836.1	238933.33 ± 27380.08			
Com_497_pos	Phellodenol H O-hexoside			4.91	531.500	Phenols	48658.67 ± 7192.24	71385.33 ± 3928.75	43986.67 ± 3127.61	334800 ± 119481.46	25936.67 ± 3467.08	36603.33 ± 16030.79			
Com_498_pos	p-Coumaraldehyde	C9H8O2	148.159	7.90	149.100	Phenols	169166.67 ± 11182.82	691666.67 ± 17679.05	216633.33 ± 29348.52	247366.67 ± 114580.4	209200 ± 7002.86	246833.33 ± 30363.39	C05608	HMDB0040986	
Com_501_pos	Betaine	C5H11NC	117.146	0.82	118.100	Alkaloids	6606333.33 ± 296664.	15040000 ± 316333.1[illegible]	56416666.67 ± 46604[illegible]	17831000 ± 11377195	11273333.33 ± 27860[illegible]	22763333.33 ± 45966[illegible]	C00719	HMDB0000043	

Com_502_pos	1,3-Dimethyluric Acid	C7H8N4C	196.163	7.09	197.000	Nucleotide and its derivates	39476.67 ± 5372.86	389500 ± 53119.49	20090 ± 5193.46	138657.67 ± 55887.16	24700 ± 4807.75	64943.33 ± 8195.76		HMDB0001857	
Com_503_pos	10-Formyl-THF	C20H23N	473.439	2.02	474.200	Amino acid and its derivatives	113543.33 ± 12964.87	24110 ± 4015.4	24326.67 ± 1116.73	102923.33 ± 79818.35	217233.33 ± 7449.98	75793.33 ± 11360.93	C00234	HMDB0000972	
Com_504_pos	14,15-Dehydrocrepenynic acid	C18H28O	276.414	14.12	277.220	Lipids	948300 ± 73936.77	1034800 ± 44164.24	6234000 ± 697924.06	748466.67 ± 523619.3	1660000 ± 70204.46	1765333.33 ± 90097.48			LMFA01031062
Com_512_pos	Sodium taurodeoxycholate	C26H44N	521.685	10.98	522.286	Lipids	26686 ± 6439.32	30896.67 ± 1386.53	117700 ± 15709.23	2656 ± 1601.6	10262.67 ± 1770.73	18253.33 ± 3871.66		HMDB0000896	
Com_518_pos	Hesperetin 5-O-glucoside	C22H24O	464.419	6.52	465.100	Flavonoids	11424.33 ± 3210.99	70960 ± 7695.74	80680 ± 8689.87	2829000 ± 1474148.1	326966.67 ± 24273.49	536166.67 ± 29187.94		HMDB0037535	LMPK12140377
Com_52_neg	N-acetylglycine	C4H7NO3	117.103	0.70	116.000	Amino acid and its derivatives	2927 ± 1143.42	4265.67 ± 501.1	20723.33 ± 3184.02	63903.33 ± 17787.8	7180.67 ± 1294.15	17560 ± 4286.42		HMDB0000532	
Com_520_pos	GDP-L-fucose	C16H25N	589.342	0.80	590.089	Saccharides	3557.33 ± 127	16866.67 ± 1949.86	4400.67 ± 995.74	6277 ± 7461.48	8251.33 ± 2861.11	8877.33 ± 1956.78	C00325	HMDB0001095	
Com_527_pos	Chinese bittersweet alkaloid II	C18H33N	339.470	6.75	340.100	Alkaloids	53530 ± 6428.51	41543.33 ± 7968.95	611433.33 ± 92917.32	48260 ± 20915.24	164880.33 ± 4694.11	150566.67 ± 26176.62			
Com_530_pos	Methyl cinnamate	C10H10O	162.185	9.88	163.073	Phenols	84607.67 ± 13905.29	39923.33 ± 1948.42	61151.33 ± 2697.09	53490.33 ± 24389.74	271666.67 ± 10964.59	48570 ± 9821.88	C06358	HMDB0033833	
Com_532_pos	Methyl linoleate	C19H33O	294.472	15.28	295.263	Lipids	532000 ± 63587.63	459766.67 ± 32447.84	1101000 ± 92101.39	249966.67 ± 193501.7	184100 ± 9714.94	393400 ± 27253.01		HMDB0034381	
Com_535_pos	D-Methionine	C5H11NC	149.211	0.91	150.100	Amino acid and its derivatives	12867.67 ± 2285.74	541672 ± 12527.28	14483.33 ± 2530.88	3019266.67 ± 216946	234200 ± 22717.54	497733.33 ± 84650.55	C00855		
Com_536_pos	D-Norvaline	C5H11NC	117.146	0.90	118.000	Amino acid and its derivatives	12483333.33 ± 63289	17976666.67 ± 23012	36190000 ± 3319769.	81193333.33 ± 31363	12916666.67 ± 74028	31343333.33 ± 17667	C01799		
Com_537_pos	Diosgenin	C27H42O	414.621	13.04	414.900	Terpenoids	1679000 ± 43150.9	1643333.33 ± 15627.6	1673000 ± 24041.63	5557000 ± 2653065.1	1497333.33 ± 56535.1	2497000 ± 193886.56	C08898		LMST01080037
Com_538_pos	Diosmetin	C16H12O	300.263	8.55	301.071	Flavonoids	33660.33 ± 2807.55	19570 ± 2757.83	76803.33 ± 7265.5	51756.67 ± 30782.47	134800 ± 9749.87	39816.67 ± 2336.87	C10038	HMDB0029676	LMPK12110824
Com_54_neg	Plantagoside	C21H22O	466.392	4.55	465.104	Flavonoids	21923.33 ± 2586.2	4677.33 ± 562.51	25805 ± 1765.67	196400 ± 40752.01	8919 ± 2471.43	28736.67 ± 1829.43	C17531		LMPK12140454
Com_540_pos	Pseudoephedrine	C10H15N	165.232	2.87	166.123	Alkaloids	65918.33 ± 10101.71	72622 ± 4163.56	20273.33 ± 3797.74	609433.33 ± 531587.5	31400 ± 4626.68	145033.33 ± 4706.26	C02765		
Com_541_pos	DL-threo-beta-Methylaspartic acid	C5H9NO4	147.129	0.74	148.100	Amino acid and its derivatives	12186.67 ± 270.1	18110 ± 2319.83	41066.67 ± 4976.67	21143.33 ± 6003.01	8931.67 ± 2584.55	14163.33 ± 2332.7			
Com_545_pos	Xanthurenic acid O-hexoside	C16H17N	367.310	1.59	368.100	Nucleotide and its derivates	142900 ± 27203.8	421033.33 ± 8491.7	4968333.33 ± 314138.	806766.67 ± 181197.8	618366.67 ± 53279.16	1156333.33 ± 49888.77			
Com_550_pos	N-p-Coumaroylputrescine derivative			8.16	398.200	Phenols	28683.33 ± 4178.22	153733.33 ± 1966.95	71367 ± 6312.91	38387 ± 45219.53	15234 ± 5523.88	27936.67 ± 5771.44			
Com_551_pos	N-p-Coumaroylagmatine	C14H20N	276.334	15.30	277.170	Phenols	229166.67 ± 29920.04	187933.33 ± 4870.55	451966.67 ± 60044.67	121480 ± 77768.43	94843.33 ± 5711.83	162733.33 ± 24067.17			
Com_552_pos	N-p-Cinnamoylputrescine	C13H18N	218.290	7.14	219.000	Phenols	155233.33 ± 11933.8	239300 ± 33521.44	218800 ± 15897.17	44294.33 ± 25399.62	451200 ± 31069.71	194866.67 ± 3015.88			
Com_56_neg	Xanthurenic acid	C10H7NC	205.167	2.36	204.030	Amino acid and its derivatives	49676.67 ± 10163.71	45110 ± 7467.05	3007333.33 ± 388076.	34413.33 ± 19396.09	13963.33 ± 2423.07	559466.67 ± 130257.7	C02470	HMDB0000881	
Com_564_pos	Cinnamoyl tyramine			4.40	268.000	Phenols	29260.33 ± 345.73	127200 ± 9749.87	46145.33 ± 4482.28	8048.33 ± 450.32	21273.33 ± 2292.14	30706.67 ± 5795.72			

Com_575_pos	(2S,3R)-2-{[{9-[(2R,3R,4S,5R)-3,4-Dihydroxy-5-(hydroxymethyl)tetrahydro-2-furanyl]-9H-purin-6-yl}(methyl)carbamoyl]amino}-3-hydroxybutanoic acid (non-preferred	C16H22N	426.380	4.06	427.160	Amino acid and its derivatives	27553.33 ± 3813.88	22290 ± 2537.18	27183.33 ± 6725.74	84760 ± 20798.15	23213.33 ± 702.44	27396.67 ± 5292.84			
Com_578_pos	Syringin	C17H24O	372.367	3.79	390.176	Phenols	5603.67 ± 837.13	11438.67 ± 1714.69	28634.33 ± 1261.76	20122.33 ± 5840.7	54696.67 ± 5412	8011 ± 1984.99	C01533		
Com_581_pos	Quercitrin	C21H20O	448.377	6.14	449.000	Flavonoids	61999 ± 11271.92	81870 ± 5126.43	69429.67 ± 7876.96	255766.67 ± 153789.5	15040 ± 3444.25	54383.33 ± 9647.54	C01750	HMDB0033751	LMPK12112171
Com_583_pos	Timosaponin Al	C33H54O	578.777	7.77	579.389	Flavonoids	24398.33 ± 1642.36	17193.33 ± 5788.01	509900 ± 16420.31	90773.33 ± 74651.53	101320 ± 8099.84	98433.33 ± 7227.95			
Com_59_neg	Physcion 8-O-Beta-D-Monoglucoside	C22H22O	446.404	5.96	445.114	Phenols	9551 ± 1623.02	152366.67 ± 1673.98	14473.33 ± 2041.44	78691.33 ± 13042.85	14456.67 ± 1844.64	37883.33 ± 2722.89			
Com_590_pos	Indole-3-carboxylic	C9H7NO2	161.157	5.87	162.150	Organic acids	15266.67 ± 3758.91	57510 ± 6864.19	125800 ± 8396.82	23696.67 ± 5484.4	12896.67 ± 866.77	45546.67 ± 7519.01	C19837	HMDB0003320	
Com_591_pos	Schaftoside	C26H28O	564.490	4.74	565.155	Flavonoids	12970 ± 764.07	555766.67 ± 33360.69	1167000 ± 158021.1	242630 ± 197189.81	364266.67 ± 28733.76	349633.33 ± 27261.25	C10181		LMPK12110207
Com_594_pos	Naringerin	C15H12O	272.253	7.80	273.078	Flavonoids	103223.33 ± 4845.04	100983.33 ± 4509.68	13436.67 ± 1185.93	89670 ± 70190.92	2140666.67 ± 75021.4	509400 ± 10133.44			LMPK12140241
Com_599_pos	N-Acetyl-L-Tyrosine	C11H13N	223.225	2.56	224.090	Amino acid and its derivatives	6342.67 ± 3225.92	72150 ± 2193.87	23650 ± 2870.2	16902.67 ± 13008.23	12136.67 ± 1087.64	20120 ± 1360.61		HMDB0000866	
Com_601_pos	C-hexosyl-apigenin O-hexosyl-O-hexoside	C33H40O	756.660	3.73	757.220	Flavonoids	27316.67 ± 2560.15	34263.33 ± 417.82	115033.33 ± 16110.73	39535.67 ± 20765.62	26220 ± 3467.11	27833.33 ± 695.62			
Com_61_neg	D-Xylonic acid	C5H10O6	166.048	0.69	165.050	Saccharides	248300 ± 78284.48	2598333.33 ± 572296.	1718333.33 ± 265977.	2608333.33 ± 125105	7653000 ± 461532.95	3804000 ± 189140.16			
Com_610_pos	N', N''-DiSinapoylspermidine	C29H39N	557.630	4.72	558.280	Phenols	25746.67 ± 2868.52	13807.67 ± 1480.36	7909.67 ± 4767.31	9481 ± 3684.76	61300 ± 8331.3	19723.33 ± 1304.36			
Com_613_pos	4-Pyridoxate	C8H9NO4	183.161	1.06	184.100	Organic acids	43200 ± 8053.21	70976.67 ± 1240.76	356300 ± 10800.93	68413.33 ± 54111.01	31643.33 ± 9250.62	86806.67 ± 4759.81	C00847	HMDB0000017	
Com_614_pos	Phloretin	C15H14O	274.269	7.75	275.092	Phenols	28160 ± 6475.19	46836.67 ± 6754.52	55206.67 ± 9796.77	44528.33 ± 17234.54	576633.33 ± 45026.31	114410 ± 27040.21	C00774	HMDB0003306	LMPK12120525
Com_618_pos	Daidzein	C15H10O	254.238	3.85	417.117	Flavonoids	18883.33 ± 4438.93	35386.67 ± 5010.34	674100 ± 113264.76	16653.33 ± 2123.56	92750 ± 1618.89	147200 ± 25510.39	C10208	HMDB0003312	LMPK12050038
Com_620_pos	D-(+)-Octopine	C9H18N4	246.264	0.70	247.139	Alkaloids	32840 ± 5667.56	262733.33 ± 21392.42	102200 ± 26036.24	70573.33 ± 30809.71	71646.67 ± 6225.29	87413.33 ± 5609.38	C04137		
Com_623_pos	N2, N2-Dimethylguanosine	C12H17N	311.294	1.59	312.130	Nucleotide and its derivates	209433.33 ± 26429.07	503166.67 ± 30525.87	600333.33 ± 6251.04	1560666.67 ± 537327.	482100 ± 53336.73	552966.67 ± 19310.16			
Com_628_pos	2-Hydroxycinnamate	C9H8O3	164.158	5.46	165.055	Phenols	283710.67 ± 43219.62	2098666.67 ± 27644.5	470766.67 ± 20493.96	927379.33 ± 78963.18	77180 ± 1350.88	441266.67 ± 36998.23	C01772	HMDB0134028	
Com_63_neg	L-Tartaric acid	C4H6O6	150.016	0.64	149.000	Organic acids	60743.33 ± 13640.11	68060 ± 9556.63	127400 ± 14921.8	106010 ± 31469.23	478000 ± 18364.28	200100 ± 4868.95	C00898		
Com_634_pos	Levodopa	C9H11NC	197.190	0.78	198.100	Amino acid and its derivatives	209800 ± 31766.75	425800 ± 55512.22	348100 ± 25604.3	5728900 ± 3788633.7	386766.67 ± 35581.11	1311000 ± 132410.98	C00355	HMDB0000181	
Com_64_neg	L-Arabinose	C5H10O5	150.053	0.71	149.000	Saccharides	249400 ± 22024.08	371300 ± 31730.43	453366.67 ± 9176.18	550033.33 ± 97595.81	2622000 ± 172860.25	1137666.67 ± 41668	C11476	HMDB0000646	
Com_644_pos	N', N'',N'''-Tri-p-cinnamoylagmatine			4.45	522.000	Phenols	15966.67 ± 4122.62	17800 ± 980.67	7653.33 ± 2044.66	22311 ± 26530.88	55546.67 ± 11366.59	18110 ± 1538.98			
Com_645_pos	Vitexin	C21H20O	432.378	4.80	433.112	Flavonoids	26405 ± 2767.14	35906.67 ± 6306.7	834633.33 ± 17157.76	190946.67 ± 120894.9	103130 ± 11071.79	182200 ± 15010.88	C01460		LMPK12110194
Com_649_pos	4,6-Dihydroxyquinoline O-hexoside			12.61	324.200	Amino acid and its derivatives	11474.33 ± 5237.62	7931 ± 2428.59	8267.33 ± 647.7	10049.33 ± 2149.02	23410 ± 5435.85	7723.67 ± 1502.68			
Com_652_pos	Sugiol	C20H28O	300.435	14.58	301.200	Terpenoids	9813.67 ± 1605.14	51055 ± 1760.99	6513.33 ± 791.08	16816.33 ± 7941.05	190933.33 ± 11015.24	35353.33 ± 5969.35		HMDB0036564	
Com_653_pos	Coumarin	C9H6O2	146.143	6.51	147.100	Phenols	928133.33 ± 139309.7	495233.33 ± 20666.29	292633.33 ± 25516.84	125738.67 ± 136249.9	25153.33 ± 2526.94	287866.67 ± 6899.44	C05851	HMDB0001218	

Com_656_pos	C-pentosyl-C-hexosyl-apigenin	C26H28O	564.490	4.70	565.160	Flavonoids	11515 ± 1984.52	166700 ± 10246.3	784733.33 ± 87430.59	190196.67 ± 199710.7	4841000 ± 154278.97	993800 ± 43818.72			
Com_657_pos	methylQuercetin O-hexoside			6.08	479.000	Flavonoids	16740 ± 1767.62	316265.67 ± 11319.76	152800 ± 11761.24	15164333.33 ± 435914	1015633.33 ± 45715.5	2827666.67 ± 304083.69			
Com_66_neg	Chlorogenic acid methyl ester	C17H20O	368.111	5.20	367.000	Phenols	23993.33 ± 825.97	9899 ± 120.53	24926.67 ± 2453.57	36710 ± 36035.05	132466.67 ± 5698.15	41840 ± 1297.25			
Com_669_pos	D-Glutamine	C5H10N2	146.145	0.65	147.100	Amino acid and its derivatives	18903333.33 ± 825604	12363333.33 ± 56922	58826666.67 ± 40328	171376666.67 ± 98993	19540000 ± 247116.7	71773333.33 ± 17920	C00819	HMDB0000641	
Com_677_pos	Quercetin O-malonylhexoside			5.46	551.000	Flavonoids	63910 ± 10282.04	74641.67 ± 3448.82	31893.33 ± 5099.18	809700 ± 119246.22	87393.33 ± 25454.64	174166.67 ± 34389.57			
Com_680_pos	Quercetin-3,4'-O-di-beta-glucopyranoside	C27H30O	626.520	4.56	627.500	Flavonoids	71221 ± 9621.08	17250 ± 4957.83	201700 ± 5040.5	3546000 ± 2231728.9	1001733.33 ± 99135.2	740400 ± 87203.94			
Com_681_pos	Quercetin	C15H10O	302.236	6.78	303.046	Flavonoids	12180.33 ± 2163.34	10966.67 ± 3626.02	22204.33 ± 2845.31	609766.67 ± 105839.3	83193.33 ± 853.87	112366.67 ± 7647.37	C00389	HMDB0005794	LMPK12110004
Com_682_pos	Pyrrole-2-Carboxylic Acid	C5H5NO2	111.099	0.73	112.100	Organic acids	336766.67 ± 21860.52	357800 ± 30875.66	1486333.33 ± 72246.4	557600 ± 437356.88	343100 ± 18719.51	520933.33 ± 34976.69		HMDB0004230	
Com_684_pos	Quercetin 5-O-hexoside			6.52	465.100	Flavonoids	42090.33 ± 13333.89	63800 ± 2388.15	94176.67 ± 10193.38	2802333.33 ± 141878	370366.67 ± 15591.52	507433.33 ± 94770.68			
Com_686_pos	N6-Succinyl Adenosine	C14H17N	383.313	1.27	384.117	Nucleotide and its derivates	811400 ± 42668.57	1455666.67 ± 36463.0	8242666.67 ± 563178.	2120333.33 ± 107492	2196666.67 ± 181698.	2464666.67 ± 118761.2			
Com_688_pos	N-Acetyl-D-tryptophan	C13H14N	246.262	5.53	247.105	Amino acid and its derivatives	231337.67 ± 33127.73	107566.67 ± 1641.81	465200 ± 55464.76	67733.33 ± 5830.55	183366.67 ± 22685.14	146566.67 ± 14634.74	C03137	HMDB0013713	
Com_69_neg	Isoacteoside	C29H36O	624.587	8.66	623.198	Phenols	5861 ± 1383.79	3132 ± 1410.35	13580 ± 179.07	114793.33 ± 53250.23	35426.67 ± 3413.84	26540 ± 5736.03		HMDB0041025	
Com_692_pos	p-Aminobenzoate	C7H7NO2	137.136	5.47	137.300	Phenols	271400 ± 54796.78	45136.67 ± 4169.53	70486.67 ± 11801.76	133867.67 ± 58569.92	150693 ± 4010.23	78900 ± 9566.01	C00568	HMDB0004992	
Com_693_pos	6-Gingerol	C17H26O	294.386	10.52	317.172	Phenols	169860 ± 51922.96	288866.67 ± 36601.03	234366.67 ± 48175.6	228700 ± 40056.54	1249000 ± 26318.56	665233.33 ± 249708.0	C10462	HMDB0005783	
Com_694_pos	p-Coumaric acid	C9H8O3	164.158	3.64	165.200	Phenols	42241.67 ± 4584.23	304256.67 ± 5915.72	102002.67 ± 6036.52	21853333.33 ± 64272	111666.67 ± 9151.44	5313000 ± 218212.43	C00811	HMDB0128076	
Com_700_pos	6-Aminohexanoic acid	C6H13NC	131.173	1.16	132.000	Amino acid and its derivatives	40440 ± 4928.67	66346.67 ± 6207.91	124933.33 ± 17446.74	316733.33 ± 93152.43	67453.33 ± 3682.76	110436.67 ± 10594.94	C02378	HMDB0001901	LMFA01100035
Com_709_pos	Tanshinone II B	C19H18O	310.347	11.16	311.000	Phenols	610533.33 ± 60168.45	327655.67 ± 16455.42	51963.33 ± 10222.75	281733.33 ± 71521.2	124833.33 ± 758.65	176200 ± 3930.22			
Com_71_neg	Fumaric Acid	C4H4O4	116.072	0.56	115.000	Organic acids	431133.33 ± 59654.64	224600 ± 5957.07	929266.67 ± 62462.86	574133.33 ± 264003.0	1762000 ± 121633.33	868566.67 ± 150952.8	C00122	HMDB0000134	
Com_711_pos	Cyanidin O-rutinoside	C27H31O	595.520	5.25	595.170	Flavonoids	22400 ± 3210.18	261721 ± 15170.13	19706.67 ± 2909.95	4443666.67 ± 173047	680200 ± 44132.6	804666.67 ± 111080.19			
Com_713_pos	L-Valine	C5H11NC	117.146	0.79	118.086	Amino acid and its derivatives	466833.33 ± 34555.3	673966.67 ± 22819.34	1424333.33 ± 133439.	3463333.33 ± 143499	515300 ± 29436.03	1264333.33 ± 70476.1	C00183	HMDB0000883	
Com_719_pos	Scopoletin	C10H8O4	192.168	4.48	193.100	Phenols	17640 ± 3982.69	14840.33 ± 1723.35	57217.67 ± 954.97	44286 ± 18210.94	110926.67 ± 11828.44	28866.67 ± 4290.71	C01752	HMDB0034344	
Com_72_neg	Betulinic acid	C30H48O	456.700	18.17	455.352	Terpenoids	18080 ± 5176.95	65270 ± 11084.21	172333.33 ± 24950.93	77440.67 ± 8171.6	44576.67 ± 26960.49	53293.33 ± 19580.48	C08619	HMDB0030094	LMPR0106140004
Com_720_pos	Scopolamine	C17H21N	303.353	5.27	304.154	Alkaloids	66543.67 ± 7651.84	317107.67 ± 11635.11	36320 ± 3947.82	2552333.33 ± 735043.	210766.67 ± 11474.71	623166.67 ± 36965.24	C01851	HMDB0003573	
Com_727_pos	Matairesinol	C20H22O	358.385	6.92	359.149	Phenols	64210 ± 10997.09	11415.33 ± 1390.04	16316.67 ± 1673.8	11660.67 ± 11255.75	28712.33 ± 2191.4	20076.67 ± 2966.9	C10682	HMDB0035698	
Com_729_pos	N-hexosyl-p-coumaroyl putrescine			7.75	397.100	Phenols	342606 ± 40243.01	1186000 ± 29131.88	113674.67 ± 7676.29	72895.33 ± 41263.69	29870.67 ± 18737.58	190933.33 ± 17178.35			
Com_730_pos	Epigoitrin	C5H7NOS	129.180	0.77	130.032	Alkaloids	1534000 ± 133673.73	9398000 ± 132592.11	2870333.33 ± 209676.	2773333.33 ± 443884.	2226000 ± 110284.48	3275666.67 ± 419537.31			
Com_731_pos	lactucopicrin	C23H22O	410.417	12.20	411.137	Terpenoids	4911 ± 2195.3	6986.67 ± 1349.41	83030 ± 4948.15	9581 ± 3562.06	5127.67 ± 1201.93	16740 ± 3927.99		HMDB0035828	
Com_732_pos	N-Feruloyltyramine	C18H19N	313.348	7.37	314.140	Phenols	25986666.67 ± 21480	11976666.67 ± 51493	6967333.33 ± 778110.	9398333.33 ± 947357	90276666.67 ± 34510	29740000 ± 1270511.	C02717	HMDB0029365	
Com_734_pos	N-Feruloylserotonin	C20H20N	352.384	14.53	353.150	Phenols	2126.33 ± 434.06	8623.67 ± 2509.48	149933.33 ± 10632.13	17162.33 ± 6728.47	47133.33 ± 2169.09	39126.67 ± 2363.42		HMDB0032759	
Com_735_pos	Feruloylputrescine	C14H20N	264.320	3.39	265.160	Phenols	833600 ± 130679.84	218306.67 ± 9201.36	355800 ± 12493.46	35666.67 ± 9085.83	84890 ± 7777.52	120976.67 ± 19777.27	C10497	HMDB0033463	
Com_739_pos	Rhodomyrtone	C26H34O	442.540	13.03	460.400	Phenols	715766.67 ± 18261.13	689033.33 ± 17303.63	685833.33 ± 16685.79	1771500 ± 794818.32	661066.67 ± 29634.48	936900 ± 66768.41			
Com_742_pos	delta-Aminolevulinic acid hydrochloride	C5H10ClN	167.590	1.17	132.100	Amino acid and its derivatives	214800 ± 21107.5	283400 ± 19991.16	553866.67 ± 53321.31	1576100 ± 554273.12	305900 ± 19948.1	534433.33 ± 21479.34			

Com_75_neg	Cyanidin O-diacetyl-hexoside-O-glyceric acid		620.100	8.46	619.100	Flavonoids	34492 ± 3677.16	35030.33 ± 3367.67	59649 ± 5822.44	103166.67 ± 37522.07	251200 ± 13205.3	53680 ± 7596.37			
Com_76_neg	Cyanidin O-acetylhexoside		490.100	8.17	489.100	Flavonoids	3181 ± 844.87	42854.67 ± 4439	45070 ± 4336.17	1850666.67 ± 192132.	27080 ± 1198.36	333200 ± 23895.19			
Com_77_neg	Uridine 5'-diphospho-D-glucose	C15H24N	566.100	0.68	565.100	Nucleotide and its derivates	9597 ± 1777.67	26847.67 ± 1039.76	3997.33 ± 2063.07	234566.67 ± 75926.29	66216.67 ± 16467.82	46740 ± 10822.1			
Com_78_neg	PinoresinolDiglucoside	C32H42O	682.675	7.91	179.056	Phenols	8784.67 ± 625.61	48828 ± 4052.32	70234 ± 5959.36	9821.33 ± 617.98	275000 ± 57292.99	52546.67 ± 7782.18			
Com_85_neg	N7-Methylguanosine	C11H17N	298.275	3.90	296.100	Nucleotide and its derivates	45893.33 ± 3305.61	63876.67 ± 5335.08	89366.67 ± 5959.55	319433.33 ± 50734.89	84526.67 ± 1812.55	129633.33 ± 5701.66	C20674		
Com_86_neg	9-Hotre	C18H30O	294.429	14.33	293.000	Lipids	10909 ± 908	11530 ± 331.76	206133.33 ± 42546.63	8679 ± 8360.69	34130 ± 3244.7	49510 ± 3115.52			LMFA02000024
Com_88_neg	L-Arabitol	C5H12O5	152.146	0.71	151.062	Saccharides	9528.67 ± 2031.87	43646.67 ± 8374.3	1123100 ± 206003.51	111720 ± 112707.54	89310 ± 8818.17	309766.67 ± 59580.83	C00532	HMDB0001851	
Com_89_neg	Malic acid	C4H6O5	134.087	0.56	133.000	Organic acids	8222333.33 ± 217763	2499000 ± 19304.58	21240000 ± 2485008.	13654666.67 ± 10879	48296666.67 ± 38986	19596666.67 ± 40547	C00711	HMDB0000156	
Com_92_neg	D-Tagatose	C6H12O6	180.156	0.62	179.058	Saccharides	616166.67 ± 87433.57	520166.67 ± 45080.69	238533.33 ± 14631.55	705466.67 ± 163720.2	2235000 ± 44452.22	989500 ± 72843.44	C00795	HMDB0003418	
Com_93_neg	Dulcitol	C6H14O6	182.172	0.69	181.072	Saccharides	107196.67 ± 12266.32	621733.33 ± 42516.69	5012000 ± 705820.09	427020 ± 378140.68	193066.67 ± 39438.17	1470666.67 ± 545588.	C01697	HMDB0000107	
Com_95_neg	Wogonin	C16H12O	284.263	9.89	283.061	Flavonoids	26500 ± 3343.92	5952 ± 294.88	10504.33 ± 2595.8	16690.67 ± 10191.8	167766.67 ± 10659.69	44846.67 ± 5840.56	C10197		LMPK12111330
Com_96_neg	Xanthosine	C10H12N	284.225	1.16	283.070	Nucleotide and its derivates	92296.67 ± 31648.2	67800 ± 18943.62	60796.67 ± 30664.76	195733.33 ± 56399.19	40253.33 ± 14401.6	71863.33 ± 17919.01	C01762	HMDB0000299	
Com_97_neg	D-Xylose	C5H10O5	150.130	0.72	149.046	Saccharides	67943.33 ± 13181.58	110000 ± 4808.33	128833.33 ± 21023.37	199300 ± 19536.12	806766.67 ± 47851.46	383733.33 ± 14183.64	C00181	HMDB0000098	
Com_98_neg	D-Xylulose	C5H10O5	150.130	0.72	149.033	Saccharides	105333.33 ± 34153.58	112566.67 ± 7922.26	892100 ± 72400.55	149033.33 ± 24771.26	507033.33 ± 11943.29	419200 ± 25358.36	C00310	HMDB0001644	
Com_99_neg	Vitexin-2-O-rhamnoside	C27H30O	578.519	7.79	577.156	Flavonoids	23736.67 ± 2229.52	7184333.33 ± 481702.	38776666.67 ± 27428	2821400 ± 2982100.3	7739000 ± 160451.45	13250000 ± 85244.75			
