# Supplementary material for: Metabolic and Transcriptomic Profile Revealing the Differential Accumulating Mechanism in Different Parts of Dendrobium nobile
Source: Int J Mol Sci. 2024 May 14;25(10):5356. doi: 10.3390/ijms25105356 (PMC11121218; doi:10.3390/ijms25105356)
Supplement: Supplementary file 1 [file ijms-25-05356-s001.zip › Table S2.pdf]

Table S2 Detailed information of differentially expressed genes in different parts of *D. nobile*

Gene ID	Relative expression levels (FPKM) Roots	Stems	Leaves	Flowers	Fruits	TF annotation	Kegg annotation	NCBI annotation	Gene name
110094239	209.86 ± 51.18	5.85 ± 0.31	330.11 ± 267.44	55.1 ± 42.7	1029.58 ± 601.08		K00020//3-hydroxyisobutyrate dehydrogenase	protein EXORDIUM-like	*DnEXORDIUML*
110112496	33.58 ± 0.42	52.52 ± 0.13	725.58 ± 26.14	28.54 ± 3.4	43.77 ± 11.7		K00026//malate dehydrogenase	malate dehydrogenase, glyoxysomal	*DnMDHG*
110099942	58.46 ± 3.5	108.15 ± 0.77	603.14 ± 28.89	61.45 ± 30.67	97.91 ± 13.91		K00051//malate dehydrogenase (NADP+)	malate dehydrogenase [NADP], chloroplastic	*DnMDHC*
110116647	645.6 ± 51.3	99.51 ± 0.09	13.66 ± 2.16	61.3 ± 23.96	19.75 ± 7.34		K00083//cinnamyl-alcohol dehydrogenase	cinnamyl alcohol dehydrogenase 2	*DnCAD2*
110098859	99.3 ± 8.53	0.09 ± 0.02	0.3 ± 0.07	0.92 ± 0.75	0.73 ± 0.53		K00083//cinnamyl-alcohol dehydrogenase	probable cinnamyl alcohol dehydrogenase 6	*DnCAD6*
110100405	1.76 ± 0.62	613.42 ± 2.12	83.01 ± 12.53	12.05 ± 8.05	10.39 ± 6.08		K00108//choline dehydrogenase	protein HOTHEAD-like	*DnHOTHEADL*
110102758	2551.72 ± 104.85	6119.15 ± 32.92	6336.95 ± 681.11	2602 ± 1.23	5508.56 ± 283.15		K00128//aldehyde dehydrogenase (NAD+)	aldehyde dehydrogenase (NAD+) like	*DnTCTPH*
110104263	27.64 ± 2	10.41 ± 0.02	2.16 ± 0.33	397.72 ± 33.06	23.08 ± 3.49		K00128//aldehyde dehydrogenase (NAD+)	aldehyde dehydrogenase family 3 member H1-like	*DnADF3H1*
110103463	4.21 ± 0.85	0.14 ± 0.11	1.74 ± 0.26	63.21 ± 6.1	0.19 ± 0.15		K00133//aspartate-semialdehyde dehydrogenase	random slug protein 5-like	*DnRSP5L*
110109552	1849.6 ± 102.54	2061.09 ± 55.59	1861.7 ± 265.52	1493.17 ± 219.69	2273.91 ± 118.45		K00134//glyceraldehyde 3-phosphate dehydrogenase	glyceraldehyde-3-phosphate dehydrogenase 2, cytosolic	*DnGPDH2C*
114579445	6.14 ± 0.22	0.04 ± 0	0.06 ± 0.04	0 ± 0	0.4 ± 0.23		K00134//glyceraldehyde 3-phosphate dehydrogenase	uncharacterized protein LOC114579445	*DnUNP5*
110106158	2.56 ± 0.15	0.25 ± 0.02	0.08 ± 0.04	0.29 ± 0.07	0.16 ± 0.04		K00279//cytokinin dehydrogenase	cytokinin dehydrogenase 3-like	*DnCKD3L*
110105324	3.78 ± 0.15	0.05 ± 0	0 ± 0	0.19 ± 0.16	0 ± 0		K00279//cytokinin dehydrogenase	cytokinin dehydrogenase 6-like	*DnCKD6L*
110110265	275.46 ± 20.99	160.89 ± 4.95	1909.03 ± 156.15	138.08 ± 64.48	374.69 ± 115.36		K00281//glycine dehydrogenase	glycine dehydrogenase (decarboxylating), mitochondrial	*DnGDHM*
110104660	51.45 ± 3.66	26.21 ± 0.48	258.83 ± 9.92	49.69 ± 21.31	38.41 ± 4.01		K00284//glutamate synthase (ferredoxin)	ferredoxin dependent glutamate synthase, chloroplastic	*DnFDGSC*
110106432	1.69 ± 0.1	0.04 ± 0.03	0 ± 0	0 ± 0	0.2 ± 0.05		K00384//thioredoxin reductase (NADPH)	putative germin-like protein 2-1	*DnGLP2-1L1*
110106435	2.68 ± 0.1	0 ± 0	0 ± 0	0 ± 0	0.05 ± 0.04		K00384//thioredoxin reductase (NADPH)	putative germin-like protein 2-1	*DnGLP2-1L2*
110103175	5.16 ± 0.22	0 ± 0	0 ± 0	0 ± 0	0.05 ± 0.04		K00422//polyphenol oxidase	polyphenol oxidase A1, chloroplastic like	*DnPPOA1C*
110099290	20.9 ± 1.32	19.64 ± 0.67	55.19 ± 9.95	1166.65 ± 720.68	11.21 ± 7.87		K00422//polyphenol oxidase	polyphenol oxidase, chloroplastic-like	*DnPPOCL*
110110340	4.97 ± 0.23	0.03 ± 0.02	0.02 ± 0.01	0 ± 0	0.02 ± 0.01		K00423//L-ascorbate oxidase	L ascorbate oxidase like	*DnAAOL*
110113505	13.03 ± 0.95	0.06 ± 0	0.42 ± 0.18	0.11 ± 0.09	0.19 ± 0.01		K00430//peroxidase	cationic peroxidase 1 like	*DnCPOD1L*
110116118	505.8 ± 41.11	39.01 ± 7.96	0.84 ± 0.18	0.5 ± 0.41	4.44 ± 0.26		K00430//peroxidase	early nodulin-75-like	*DnEN75L*
110101959	82.52 ± 7.13	1.88 ± 0.07	0.02 ± 0.01	0 ± 0	0.09 ± 0.04		K00430//peroxidase	peroxidase 11-like	*DnPOD11L*
110091820	18.77 ± 0.95	0.96 ± 0.11	0.47 ± 0.03	1.13 ± 0.29	3.06 ± 1.24		K00430//peroxidase	peroxidase 26	*DnPOD26*
110114611	8.43 ± 0.54	0.11 ± 0	0.03 ± 0.02	0 ± 0	0.73 ± 0.29		K00430//peroxidase	peroxidase 40	*DnPOD40*
110099301	1381.79 ± 186.5	1798.52 ± 110.27	2.72 ± 0.53	155.19 ± 114.94	4.79 ± 0.07		K00430//peroxidase	peroxidase 42-like	*DnPOD42L*
110098653	8.45 ± 0.29	1.72 ± 0.17	0.1 ± 0.04	0.16 ± 0.13	1.16 ± 0.65		K00430//peroxidase	peroxidase 4-like	*DnPOD4L*
110110655	36.73 ± 1.25	0.4 ± 0.09	0.58 ± 0.09	2.33 ± 1.5	1.03 ± 0.21		K00430//peroxidase	peroxidase 51-like	*DnPOD51L*
110109322	3.51 ± 0.04	0.03 ± 0	0 ± 0	0.04 ± 0.03	0.28 ± 0.07		K00430//peroxidase	peroxidase 5-like	*DnPOD5L*
110100325	0.45 ± 0	0.03 ± 0.02	0 ± 0	0 ± 0	0 ± 0		K00430//peroxidase	peroxidase P7-like	*DnPODP7L1*
110106139	25.08 ± 2.47	0.54 ± 0.1	0 ± 0	0 ± 0	0.03 ± 0.02		K00430//peroxidase	peroxidase P7-like	*DnPODP7L2*
110106141	6.15 ± 0.53	0.2 ± 0.08	0 ± 0	0 ± 0	0 ± 0		K00430//peroxidase	peroxidase P7-like	*DnPODP7L3*
110110786	44.02 ± 2.54	0.96 ± 0.2	0 ± 0	0 ± 0	0.03 ± 0.02		K00430//peroxidase	peroxidase P7-like	*DnPODP7L4*
110093715	100.8 ± 10.88	0.2 ± 0.05	0.03 ± 0.02	1.06 ± 0.75	0.42 ± 0.16		K00430//peroxidase	peroxidase-like	*DnPODL*
110094007	242.59 ± 15.25	2.18 ± 0.01	13.56 ± 2.49	0.7 ± 0.38	0.77 ± 0.28		K00430//peroxidase	protein PELPK1-like	*DnPELPK1L*
110112818	22.75 ± 0.6	0.37 ± 0.02	4.41 ± 1.71	0.4 ± 0.02	0.2 ± 0		K00430//peroxidase	transmembrane protein 45B-like	*DnTMP45BL*
110106362	17.71 ± 0.66	1.43 ± 0.04	0.15 ± 0.04	77.06 ± 6.14	0.26 ± 0.01		K00454//lipoxygenase	lipoxygenase 8, chloroplastic	*DnLPG8C*
110106800	19.69 ± 0.2	0.06 ± 0	0.24 ± 0.2	0 ± 0	0.23 ± 0.06		K00475//naringenin 3-dioxygenase	naringenin,2-oxoglutarate 3-dioxygenase-like	*DnNODL*
110095634	18.42 ± 2.1	0.04 ± 0.01	0.1 ± 0.01	0 ± 0	0.17 ± 0.09		K00512//steroid 17alpha-monooxygenase / 17alpha-hydroxyprogesterone deacetylase	cytochrome P450 76C2-like	*DnCYP76C2L*
110106863	7.73 ± 0.05	41.15 ± 0.98	0.2 ± 0.02	1.58 ± 1.25	2.98 ± 0.31		K00512//steroid 17alpha-monooxygenase / 17alpha-hydroxyprogesterone deacetylase	cytochrome P450 CYP71D312-like	*DnCYP71D312L*
110115676	0.57 ± 0.14	1.54 ± 0.13	90.08 ± 0.52	0 ± 0	17.63 ± 3.67		K00512//steroid 17alpha-monooxygenase / 17alpha-hydroxyprogesterone deacetylase	isoflavone 2'-hydroxylase-like	*DnIFHL*
110106530	1.52 ± 0.04	0 ± 0	0 ± 0	0 ± 0	0.14 ± 0.11		K00512//steroid 17alpha-monooxygenase / 17alpha-hydroxyprogesterone deacetylase	premnaspirodiene oxygenase-like	*DnPSOL*
110098637	176.88 ± 10.07	74.04 ± 3.96	411.29 ± 47.77	80.93 ± 49.44	360.27 ± 56.11	NAC	K00521//ferric-chelate reductase	NAC domain-containing protein 2	*DnNAC2L3*

110104614	0.53 ± 0.14	0.55 ± 0.08	5.54 ± 0.38	0.48 ± 0.21	0.42 ± 0.18	NAC	K00521//ferric-chelate reductase	NAC domain-containing protein 35-like	*DnNAC35L*
110098556	3.51 ± 0.09	0 ± 0	0.79 ± 0.13	0 ± 0	0 ± 0	NAC	K00521//ferric-chelate reductase	NAC domain-containing protein 67-like	*DnNAC67L*
110098761	295.34 ± 4.04	20.49 ± 0.34	187.05 ± 83.19	35.34 ± 17.6	351.94 ± 31.55	NAC	K00521//ferric-chelate reductase	NAC domain-containing protein 68-like	*DnNAC68L*
110111459	404.75 ± 21.56	56.08 ± 1.62	175.46 ± 123.5	206.44 ± 104.98	60.69 ± 12.08	NAC	K00521//ferric-chelate reductase	NAC domain-containing protein 68-like	*DnNAC68L2*
110092932	323.72 ± 7.34	14.23 ± 0.12	222.62 ± 121.38	58.01 ± 30.98	933.53 ± 51.98	NAC	K00521//ferric-chelate reductase	NAC domain-containing protein 68-like	*DnNAC68L1*
110100119	138.86 ± 5.53	66.3 ± 0.04	314.15 ± 28.6	374.93 ± 252.51	129.76 ± 9.46	NAC	K00521//ferric-chelate reductase	NAC domain-containing protein 83-like	*DnNAC83L*
110105798	11.26 ± 4.16	8.25 ± 0.56	48.04 ± 7.15	2.92 ± 0.27	341.68 ± 77.02	NAC	K00521//ferric-chelate reductase	NAC transcription factor 29-like	*DnNAC29L*
110104101	13.89 ± 2.79	23.91 ± 0.73	762.22 ± 154.44	2.15 ± 0.31	104.25 ± 21.12	NAC	K00521//ferric-chelate reductase	NAC transcription factor 32-like	*DnNAC32L*
110095790	46.68 ± 0.32	9.34 ± 0.29	21.5 ± 1.38	53.09 ± 30.06	237.7 ± 57.18	NAC	K00521//ferric-chelate reductase	NAC transcription factor ONAC010-like	*DnNAC10L*
110105195	2.77 ± 0.26	0 ± 0	0 ± 0	0 ± 0	0.06 ± 0.04	NAC	K00521//ferric-chelate reductase	protein BEARSKIN2-like	*DnNAC-BEARSKIN2*
110106875	40.05 ± 1.64	0.36 ± 0.12	0.04 ± 0.03	0.05 ± 0.04	0.89 ± 0.47	NAC	K00521//ferric-chelate reductase	protein CUP-SHAPED COTYLEDON 1-like	*DnNAC-CSC1L*
110101259	0 ± 0	3.05 ± 0.06	0 ± 0	0 ± 0	0.03 ± 0.02	NAC	K00521//ferric-chelate reductase	protein CUP-SHAPED COTYLEDON 3-like	*DnNAC-CSC3L*
110108485	16.95 ± 0.89	0 ± 0	0 ± 0	0 ± 0	0.04 ± 0.03	NAC	K00521//ferric-chelate reductase	protein SOMBRERO-like	*DnNAC-SOMBRERO*
110107876	85.8 ± 1.7	86.96 ± 1.11	723.2 ± 151.9	5.2 ± 0.4	103.22 ± 2.27	NAC	K00521//ferric-chelate reductase	NAC domain containing protein 2	*DnNAC2L1*
114578614	33.7 ± 1.6	55.46 ± 0.36	567.23 ± 119.69	0 ± 0	40.54 ± 10.19	NAC	K00521//ferric-chelate reductase	NAC domain containing protein 2 like, partial	*DnNAC2L2*
110093667	1679.56 ± 148.6[illegible]	645.59 ± 4.98	103.07 ± 0.49	168.82 ± 17.21	320.2 ± 62.14		K00549//5-methyltetrahydropteroyltriglutamate--homocysteine methyltransferase	5 methyltetrahydropteroyltriglutamate homocysteine methyltransferase 2	*DnMHM2*
110113335	1395.14 ± 101.7[illegible]	150.4 ± 7.38	83.87 ± 3.75	240.04 ± 120.09	18.81 ± 1.19		K00588//caffeoyl-CoA O-methyltransferase	caffeoyl-CoA O-methyltransferase	*DnCCOMT*
110092466	1627.51 ± 14.66	550.29 ± 22.56	280.78 ± 23.77	34.09 ± 13.66	437.27 ± 145.66		K00588//caffeoyl-CoA O-methyltransferase	putative caffeoyl-CoA O-methyltransferase At1g67980	*DnCCOMTL1*
110114871	458.39 ± 14.44	0.91 ± 0.02	0 ± 0	0.12 ± 0.01	0.59 ± 0.27		K00588//caffeoyl-CoA O-methyltransferase	putative caffeoyl-CoA O-methyltransferase At1g67980	*DnCCOMTL*
110114476	280.29 ± 17.93	340.17 ± 1.72	1939.3 ± 150.47	130.44 ± 8.06	195.21 ± 4.96		K00600//glycine hydroxymethyltransferase	serine hydroxymethyltransferase, mitochondrial	*DnSHMTM*
110106260	0.47 ± 0.03	3.87 ± 0.12	0.18 ± 0.01	0.25 ± 0.1	0.15 ± 0.06		K00679//phospholipid:diacylglycerol acyltransferase	GDSL esterase/lipase 7	*DnGDSL7*
110105207	0.7 ± 0.08	289.92 ± 9.25	9.27 ± 0.89	1792.49 ± 954.1	7.72 ± 0.49		K00679//phospholipid:diacylglycerol acyltransferase	GDSL esterase/lipase APG	*DnAPGL*
110092180	0.13 ± 0.07	10.72 ± 0.12	0 ± 0	0.18 ± 0.11	0.14 ± 0		K00679//phospholipid:diacylglycerol acyltransferase	GDSL esterase/lipase At1g29670-like	*DnGDSLL1*
110115108	0 ± 0	5.76 ± 0.09	1.1 ± 0.28	0.36 ± 0.25	0.1 ± 0.04		K00679//phospholipid:diacylglycerol acyltransferase	GDSL esterase/lipase At1g33811	*DnGDSLL2*
110098250	51.79 ± 2.5	3.73 ± 0.08	8.32 ± 0.08	0.96 ± 0.61	1 ± 0.23		K00679//phospholipid:diacylglycerol acyltransferase	GDSL esterase/lipase At2g23540	*DnGDSLL4*
110103964	7.48 ± 0.66	0.92 ± 0.04	1.17 ± 0.03	0.12 ± 0.02	0.42 ± 0.3		K00679//phospholipid:diacylglycerol acyltransferase	GDSL esterase/lipase At2g23540-like	*DnGDSLL5*
110104384	2.74 ± 0.22	1153.62 ± 39.57	0.42 ± 0.07	3.39 ± 2.6	0.27 ± 0.17		K00679//phospholipid:diacylglycerol acyltransferase	GDSL esterase/lipase At5g33370	*DnGDSLL9*
110113970	0 ± 0	19.58 ± 0.98	0 ± 0	0.22 ± 0.06	0 ± 0		K00679//phospholipid:diacylglycerol acyltransferase	GDSL esterase/lipase At5g33370	*DnGDSLL10*
110109094	8.27 ± 1.49	58.56 ± 1.37	0 ± 0	0.26 ± 0.2	0 ± 0		K00679//phospholipid:diacylglycerol acyltransferase	GDSL esterase/lipase At5g45960	*DnGDSLL8*
110112875	7.85 ± 0.71	0.52 ± 0.02	0.26 ± 0.04	1.25 ± 0.19	0.7 ± 0.24		K00679//phospholipid:diacylglycerol acyltransferase	GDSL esterase/lipase EXL3-like	*DnGDSL-EXL3L*
110111780	0.46 ± 0.12	2.06 ± 0.16	0.29 ± 0	0.12 ± 0.04	0 ± 0		K00784//ribonuclease Z	Domain of unknown function (DUF4220)	*DnDUF4220*
110099186	2502.23 ± 49.29	935.71 ± 15.29	268.86 ± 10.68	923.38 ± 350.55	387.13 ± 32.92		K00789//S-adenosylmethionine synthetase	S-adenosylmethionine synthase	*DnSAMS*
110115448	1570.3 ± 111.75	311.16 ± 8.21	137.83 ± 37.68	225.85 ± 34.61	482.36 ± 111.87		K00789//S-adenosylmethionine synthetase	S-adenosylmethionine synthase 5	*DnSAMS5*
110099092	12.31 ± 0.07	0.56 ± 0.03	0.12 ± 0.1	1.05 ± 0.5	0.72 ± 0.53		K00799//glutathione S-transferase	glutathione S-transferase GSTU6	*DnGST6L1*
110106556	3.12 ± 0.04	0.25 ± 0.13	0.08 ± 0.07	0.27 ± 0.16	0.28 ± 0.07		K00799//glutathione S-transferase	glutathione S-transferase GSTU6	*DnGST6L2*
110103397	15.21 ± 1.66	0.7 ± 0.02	0.24 ± 0.05	0.31 ± 0.06	0.09 ± 0.02		K00799//glutathione S-transferase	glutathione S-transferase parC	*DnGST-PARC*
110106491	26.04 ± 0.08	0.59 ± 0.12	0.43 ± 0.01	2.85 ± 1.47	0.71 ± 0.47		K00799//glutathione S-transferase	glutathione S-transferase U17-like	*DnGST17L1*
110115479	4.98 ± 0.29	0.3 ± 0.02	0.55 ± 0.27	0.09 ± 0.02	0.76 ± 0.16		K00799//glutathione S-transferase	glutathione S-transferase U17-like	*DnGST17L2*
110101865	15.32 ± 0.93	0 ± 0	0.18 ± 0.14	0.04 ± 0.03	0.14 ± 0.05		K00799//glutathione S-transferase	glutathione S-transferase U18-like	*DnGST18L*
110103395	477.88 ± 23.64	5.25 ± 0.76	62.01 ± 4.4	20.68 ± 11.06	33.99 ± 5.18		K00799//glutathione S-transferase	glutathione S-transferase U24-like	*DnGST24L*

110114969	19.59 ± 2.12	0.97 ± 0.09	0 ± 0	0.13 ± 0.01	0.1 ± 0.08	K00799//glutathione S-transferase	glutathione transferase GST 23-like	*DnGST23L*
110116428	4.77 ± 0.35	47.13 ± 1.45	2.36 ± 0.09	1100.21 ± 526.2	12.22 ± 5.08	K00799//glutathione S-transferase	probable glutathione S-transferase GSTF1	*DnGSTF1L*
110113711	36.39 ± 0.27	43.56 ± 0.81	44.36 ± 4.58	1336.63 ± 1026.	34.11 ± 3.04	K00801//farnesyl-diphosphate farnesyltransferase	squalene synthase	*DnSQSL*
110092540	48.63 ± 2	33.22 ± 0.37	1201.91 ± 111.2	18.87 ± 2.99	177.91 ± 103.5	K00830//alanine-glyoxylate transaminase / serine-glyoxylate transaminase / serine-pyruvate transaminase	serine--glyoxylate aminotransferase	*DnSGAT*
110093520	3.09 ± 0.22	0 ± 0	0 ± 0	0 ± 0	0 ± 0	K00850//6-phosphofructokinase 1	purine permease 3-like	*DnPPE3L3*
110114945	2.54 ± 0.24	6.42 ± 0.49	2.12 ± 0.33	2.36 ± 0.07	81.14 ± 1.33	K00851//gluconokinase	Nucleoside/nucleotide kinase	*DnNNKL*
110100865	36.21 ± 3.83	0.19 ± 0.03	0.3 ± 0.02	0.07 ± 0.02	0.34 ± 0.13	K00856//adenosine kinase	germin-like protein 9-3	*DnGLP9-3.1*
110100867	58.46 ± 4.06	0.11 ± 0.03	0.27 ± 0.03	3.61 ± 1.69	0.17 ± 0.02	K00856//adenosine kinase	germin-like protein 9-3	*DnGLP9-3.2*
110109891	126.72 ± 6.39	0.77 ± 0	0.93 ± 0.53	2.59 ± 1.94	1.74 ± 1.42	K00856//adenosine kinase	putative germin like protein 9 2	*DnGLP9-2*
110114184	235.91 ± 13.85	1409.06 ± 9.64	339.93 ± 14.86	1202.87 ± 314.1	102.37 ± 2.23	K00966//mannose-1-phosphate guanylyltransferase	probable mannose-1-phosphate guanylyltransferase 1	*DnMPGT1*
110093850	1.85 ± 0.56	15.8 ± 0.29	2103.83 ± 411.1	212 ± 173	1000.89 ± 673.94	K01051//pectinesterase	21 kDa protein-like	*Dn21KPL*
110093878	3.69 ± 0.02	0.55 ± 0.05	0.19 ± 0.04	0.08 ± 0.03	0.04 ± 0.03	K01051//pectinesterase	pectinesterase 2-like	*DnPEC2L*
110098512	3.88 ± 0.34	15.77 ± 0.15	2.19 ± 1.33	1.7 ± 1.32	0.16 ± 0.09	K01051//pectinesterase	pectinesterase 53	*DnPEC53*
110113172	2.41 ± 0.23	19.29 ± 0.33	0.02 ± 0.02	0.21 ± 0.11	0.3 ± 0.18	K01051//pectinesterase	pectinesterase 68	*DnPEC68*
110108958	37.42 ± 0.48	170.53 ± 6.65	1579.97 ± 290.8	482.42 ± 322.39	270.46 ± 128.9	K01051//pectinesterase	pectinesterase inhibitor 9-like	*DnPI9L*
110108976	25.14 ± 0.92	0.42 ± 0.03	0.53 ± 0.12	0.42 ± 0.3	0.3 ± 0.1	K01051//pectinesterase	pectinesterase inhibitor 9-like	*DnPEI9L*
110101606	8.69 ± 0.21	0.12 ± 0.05	0.14 ± 0.08	0.14 ± 0.04	0.05 ± 0	K01051//pectinesterase	pectinesterase/pectinesterase inhibitor 12	*DnPEI12*
110103997	11.26 ± 0.98	123.47 ± 6.58	0.1 ± 0.02	6.66 ± 5.21	0.16 ± 0.03	K01051//pectinesterase	pectinesterase-like	*DnPECL1*
110109782	1.51 ± 0.08	0.02 ± 0.01	0 ± 0	0 ± 0	0 ± 0	K01051//pectinesterase	pectinesterase-like	*DnPECL2*
110093232	17.01 ± 1.62	1.49 ± 0.02	1.37 ± 0.37	0.11 ± 0.06	0.39 ± 0.07	K01051//pectinesterase	protein MIZU-KUSSEI 1-like	*DnMK1L*
110102935	21.89 ± 4.17	33.92 ± 0.52	214.88 ± 2.18	8.43 ± 4.96	49.52 ± 13.46	K01092//myo-inositol-1(or 4)-monophosphatase	protein CURVATURE THYLAKOID 1B, chloroplastic	*DnCT1BC*
110108454	46.52 ± 1.04	127.63 ± 1.29	576.88 ± 36.21	0.99 ± 0	55.36 ± 26.52	K01100//sedoheptulose-bisphosphatase	sedoheptulose-1,7-bisphosphatase, chloroplastic	*DnSBPC*
110116198	1.01 ± 0.13	1.28 ± 0.05	23.5 ± 2.31	1.38 ± 0.28	1.92 ± 0.44	K01103//6-phosphofructo-2-kinase / fructose-2,6-biphosphatase 3	6 phosphofructo 2 kinase/fructose 2,6 bisphosphatase like	*DnPFK/FBPL2*
110115663	3.64 ± 0.19	4.9 ± 0.61	45.98 ± 0.33	4.85 ± 1.95	4.17 ± 0.9	K01103//6-phosphofructo-2-kinase / fructose-2,6-biphosphatase 3	6-phosphofructo-2-kinase/fructose-2,6-bisphosphatase-like	*DnPFK/FBPL1*
110092219	29.08 ± 0.82	0.67 ± 0.08	0.15 ± 0.06	0.09 ± 0.02	0.55 ± 0.28	K01179//endoglucanase	endoglucanase 22-like	*DnEG22L*
110108689	2.26 ± 0.24	1.88 ± 0.11	1.73 ± 0.13	9.72 ± 0.19	1.66 ± 0.36	K01181//endo-1,4-beta-xylanase	Carbohydrate binding domain	*DnCBM4-9*
110115418	47.21 ± 2.31	2.43 ± 0.18	0.38 ± 0.01	0.23 ± 0.05	3.54 ± 1.57	K01181//endo-1,4-beta-xylanase	endo 1,4 beta xylanase 1	*DnEBX1*
110106661	4.39 ± 0.33	0 ± 0	0 ± 0	0 ± 0	0 ± 0	K01183//chitinase	chitinase 4-like	*DnCHI4L1*
110106969	7.64 ± 0.16	0 ± 0	0 ± 0	0 ± 0	0.09 ± 0.07	K01183//chitinase	chitinase 4-like	*DnCHI4L2*
114579173	0 ± 0	0.24 ± 0.01	0.06 ± 0.01	0 ± 0	0.02 ± 0.02	K01183//chitinase	Reverse transcriptases	*DnRT-LTR*
110103774	1.76 ± 0.16	0 ± 0	0 ± 0	0 ± 0	0 ± 0	K01184//polygalacturonase	acanthoscurrin-2-like	*DnACA2L*
110115317	14.54 ± 0.89	0.13 ± 0.03	0.02 ± 0.01	0 ± 0	0.02 ± 0.01	K01184//polygalacturonase	polygalacturonase QRT3-like	*DnQRT3L*
110095574	24.86 ± 0.09	0 ± 0	0 ± 0	0.03 ± 0.02	0.05 ± 0.04	K01184//polygalacturonase	polygalacturonase-like	*DnPGTL1*
110110600	1.03 ± 0.06	0.16 ± 0.02	0.41 ± 0.16	15.66 ± 0.66	0.3 ± 0.04	K01191//alpha-mannosidase	Protein of unknown function (DUF1218	*DnDUF1218*
110092026	0.3 ± 0.02	0.02 ± 0.01	0 ± 0	0 ± 0	0.01 ± 0.01	K01191//alpha-mannosidase	Protein of unknown function (DUF674)	*DnDUF674*
110097456	293.63 ± 23.29	17.78 ± 1.38	16.24 ± 0.88	5.55 ± 3.49	7.4 ± 1.89	K01206//alpha-L-fucosidase	GDSL esterase/lipase At2g27360-like	*DnGDSLL6*
110116532	0 ± 0	18.21 ± 1.04	0 ± 0	0 ± 0	0.1 ± 0.04	K01206//alpha-L-fucosidase	GDSL esterase/lipase At5g45910	*DnGDSLL11*
110116501	1.04 ± 0.13	76.43 ± 1.84	2.62 ± 0.33	5.39 ± 4.31	0.29 ± 0.13	K01206//alpha-L-fucosidase	GDSL esterase/lipase At5g45910-like	*DnGDSLL7*
110116052	18.63 ± 1.37	4.41 ± 0.11	2.45 ± 0.2	3.95 ± 0.37	2.56 ± 0.07	K01213//galacturan 1,4-alpha-galacturonidase	polygalacturonase At1g80170	*DnPGTL2*
110100614	1773.43 ± 217.0	1006.84 ± 25.53	150.47 ± 3.27	457.08 ± 127.06	1045.99 ± 98.97	K01251//adenosylhomocysteinase	adenosylhomocysteinase	*DnAHC*
110105585	0.02 ± 0.02	0.81 ± 0.01	0 ± 0	0 ± 0	0 ± 0	K01255//leucyl aminopeptidase	uncharacterized protein LOC110105585	*DnUNP2*
110103805	0.73 ± 0.14	0.64 ± 0.07	0.46 ± 0.04	0.45 ± 0.29	3.07 ± 0.2	K01322//prolyl oligopeptidase	F box/FBD/LRR repeat protein At1g13570 like	*DnFBD/LRRL*
110098906	15.99 ± 0.4	0 ± 0	0 ± 0	0 ± 0	0 ± 0	K01426//amidase	amidase At4g34880	*DnAMIL*
110102011	1.87 ± 0.23	3.06 ± 0.08	14.43 ± 0.97	1.74 ± 0.98	1.5 ± 0.66	K01433//formyltetrahydrofolate deformylase	formyltetrahydrofolate deformylase 2, mitochondrial-like	*DnFTHD2M*
110095411	1.62 ± 0.04	0.26 ± 0	0.06 ± 0.04	0 ± 0	0 ± 0	K01510//apyrase	Protein of unknown function (DUF679)	*DnDUF679*
114578342	5.34 ± 1.21	22.71 ± 0.28	2.65 ± 0.09	0 ± 0	1.23 ± 0.52	K01520//dUTP pyrophosphatase	deoxyuridine 5'-triphosphate nucleotidohydrolase	*DnDTNH*

110101076	1.48 ± 0.14	0 ± 0	0 ± 0	0.02 ± 0.02	0 ± 0		K01528//dynamin GTPase	Reverse transcriptase (RNA-dependent DNA polymerase)	*DnRVT2L1*
110102141	62.07 ± 0.81	105.94 ± 1.47	110.82 ± 11.58	261.03 ± 39.42	67.61 ± 14.43		K01533//Cu2+-exporting ATPase	copper-transporting ATPase PAA1, chloroplastic-like	*DnPAA1CL*
110111407	116.28 ± 5.51	72.93 ± 4.21	135.48 ± 7.61	211.75 ± 44.98	119.18 ± 8.51		K01535//H+-transporting ATPase	plasma membrane ATPase	*DnATP-3AH*
110094010	48.62 ± 0.7	29.16 ± 2.47	113.98 ± 82.75	6.93 ± 1.13	200.77 ± 15.22		K01537//Ca2+-transporting ATPase	GTPase activating protein 1-like	*DnGAP1L*
110104892	132.54 ± 0.5	1.06 ± 0.31	5.75 ± 0.54	0.77 ± 0.59	0 ± 0		K01568//pyruvate decarboxylase	RNA-binding protein 12-like	*DnRBP12L*
110115752	1.54 ± 0.14	16.27 ± 0.86	0.67 ± 0.5	0.57 ± 0.44	0.67 ± 0.46		K01593//aromatic-L-amino-acid/L-tryptophan decarboxylase	tyrosine/DOPA decarboxylase 2	*DnTDD2*
110093044	88.3 ± 3.22	748.91 ± 27.13	4.34 ± 1.33	28.01 ± 22.69	32.12 ± 12.32		K01602//ribulose-bisphosphate carboxylase small chain	ribulose bisphosphate carboxylase small chain clone 512-like	*DnRBC512L*
110109052	426.8 ± 21.48	386.12 ± 5.19	14307.25 ± 289.	1357.47 ± 788.8	822.89 ± 593.01		K01602//ribulose-bisphosphate carboxylase small chain	ribulose bisphosphate carboxylase small chain, chloroplastic-like	*DnRBCCL1*
110109053	78.88 ± 3.34	466.99 ± 8.86	52.74 ± 5.21	58.92 ± 24.94	34.05 ± 14.55		K01602//ribulose-bisphosphate carboxylase small chain	ribulose bisphosphate carboxylase small chain, chloroplastic-like	*DnRBCCL2*
110104806	130.35 ± 1.29	950.6 ± 9.64	3242.52 ± 135.1	27.38 ± 19.19	326.75 ± 149.72		K01623//fructose-bisphosphate aldolase, class I	fructose-bisphosphate aldolase 1, chloroplastic	*DnFBA1C*
110114646	637.74 ± 18.26	1093.28 ± 19.84	1500.57 ± 1.02	284.44 ± 6.65	1041.06 ± 289.58		K01623//fructose-bisphosphate aldolase, class I	fructose-bisphosphate aldolase 1, cytoplasmic-like	*DnFBA1CL*
110107472	98.77 ± 6.61	444.84 ± 8.37	2596.52 ± 247.6	5.57 ± 2.83	95.35 ± 49.9		K01623//fructose-bisphosphate aldolase, class I	fructose-bisphosphate aldolase, chloroplastic-like	*DnFBAC*
110092446	9.1 ± 0.85	0 ± 0	0 ± 0	0 ± 0	0 ± 0		K01649//2-isopropylmalate synthase	isopropylmalate synthase 2, chloroplastic like	*DnIPS2C*
110113985	0 ± 0	0 ± 0	0 ± 0	0 ± 0	7.58 ± 0.62	ABI3VP1	K01653//acetolactate synthase I/III small subunit	regulatory protein viviparous-1-like	*DnABI3VP1L*
110104411	0.53 ± 0.07	13.61 ± 1.19	0.88 ± 0.08	0.56 ± 0.16	1.46 ± 0.38		K01657//anthranilate synthase component I	anthranilate synthase alpha subunit 2, chloroplastic like	*DnANSA2C*
114580706	23.22 ± 1.36	9.03 ± 0.43	2970.22 ± 273.7	0 ± 0	44.23 ± 8.54		K01673//carbonic anhydrase	Carbonic anhydrases	*DnCAL*
110108695	285.94 ± 5.49	1264.15 ± 94.29	53.88 ± 4.38	1142.24 ± 509.2	113.01 ± 55.98		K01723//hydroperoxide dehydratase	allene oxide synthase 2-like	*DnAOS2L1*
110106376	116.46 ± 4.47	0.23 ± 0.02	0.03 ± 0.02	1.64 ± 1.28	0.5 ± 0.27		K01723//hydroperoxide dehydratase	allene oxide synthase 2-like	*DnAOS2L*
110113714	4.82 ± 0.39	0 ± 0	0 ± 0	0 ± 0	0 ± 0		K01728//pectate lyase	protein GRIM REAPER-like	*DnGRL*
110098644	12.58 ± 1.41	0 ± 0	0 ± 0	0 ± 0	0 ± 0		K01728//pectate lyase	stigma-specific STIG1-like protein 1	*DnSTIG1L1*
110101471	48.94 ± 1.32	60.97 ± 0.11	288.52 ± 12.29	47.82 ± 2.86	58.47 ± 10.39		K01738//cysteine synthase	cysteine synthase	*DnCYS*
110106891	17.34 ± 0.45	0.52 ± 0.09	1.95 ± 1.24	0.22 ± 0.18	2.24 ± 0.39		K01762//1-aminocyclopropane-1-carboxylate synthase	1-aminocyclopropane-1-carboxylate synthase 7-like	*DnACS7L*
110100173	0.13 ± 0.11	0.57 ± 0.18	0.58 ± 0.29	9.79 ± 0.03	0.63 ± 0.28		K01770//2-C-methyl-D-erythritol 2,4-cyclodiphosphate synthase	2-C-methyl-D-erythritol 2,4-cyclodiphosphate synthase, chloroplastic-like	*DnMECPS*
110108613	32.04 ± 0.75	55.51 ± 0.84	226.5 ± 14.79	37.5 ± 1.48	36.89 ± 5.17		K01783//ribulose-phosphate 3-epimerase	ribulose-phosphate 3-epimerase, chloroplastic	*DnRPEC*
110115347	102.07 ± 8.64	18.17 ± 0.35	7.16 ± 0.08	13.78 ± 5.61	17.84 ± 1.51		K01859//chalcone isomerase	vacuolar-sorting receptor 6-like	*DnVSR6L*
110108977	5.16 ± 0.27	1.15 ± 0.06	0.07 ± 0.02	0.1 ± 0.06	0.15 ± 0.12	OFP	K01870//isoleucyl-tRNA synthetase	transcription repressor OFP8-like	*DnOFP8L*
110099881	1.26 ± 0.09	0 ± 0	0 ± 0	0 ± 0	0 ± 0		K01870//isoleucyl-tRNA synthetase	transcriptional regulator TAC1-like	*DnTAC1L*
110109932	907.58 ± 68.78	60.41 ± 1.92	28.8 ± 0.14	24.47 ± 1.92	7.19 ± 4.03		K01904//4-coumarate--CoA ligase	4 coumarate CoA ligase	*Dn4CLL*
110116024	44.96 ± 0.68	0 ± 0	0.35 ± 0.28	0.02 ± 0.02	1.07 ± 0.55		K01904//4-coumarate--CoA ligase	4 coumarate CoA ligase 1 like	*Dn4CL1L*
110116261	20.49 ± 0.58	3.28 ± 0.09	0 ± 0	1.4 ± 1.14	0.02 ± 0.01		K01904//4-coumarate--CoA ligase	4-coumarate--CoA ligase 2	*Dn4CL2L*
110104840	0 ± 0	0 ± 0	0 ± 0	0 ± 0	0.13 ± 0		K01904//4-coumarate--CoA ligase	4-coumarate--CoA ligase 3-like	*Dn4CL3L1*
110112707	0.32 ± 0.03	0.02 ± 0.01	0 ± 0	0.01 ± 0.01	0.04 ± 0		K01904//4-coumarate--CoA ligase	4-coumarate--CoA ligase 3-like	*Dn4CL3L2*
110092480	357.53 ± 12.24	412.67 ± 4.79	3520.58 ± 316.81	509.23 ± 193.18	501.38 ± 76.69		K01915//glutamine synthetase	glutamine synthetase nodule isozyme	*DnGSNI*
110094013	74.54 ± 1.39	138.98 ± 10.28	928.81 ± 39.35	71.87 ± 1.78	80.87 ± 26.73		K02109//F-type H+-transporting ATPase subunit b	ATP synthase subunit b', chloroplastic	*DnASSBC*
114578354	11.89 ± 2.11	7.73 ± 1.45	90.19 ± 0.01	0 ± 0	14.67 ± 1.51		K02113//F-type H+-transporting ATPase subunit delta	uncharacterized protein LOC114578354	*DnUNP4*
114579004	0.04 ± 0.03	0 ± 0	0.34 ± 0	0 ± 0	0.07 ± 0		K02126//F-type H+-transporting ATPase subunit a	ATP synthase subunit 6 subunit A	*DnASS6A*
110094450	0.08 ± 0.06	0 ± 0	0 ± 0	21.75 ± 2	0 ± 0		K02260//cytochrome c oxidase assembly protein subunit 17	Cytochrome C oxidase copper chaperone (COX17)	*DnCOX17*
110111885	32.72 ± 0.17	43.91 ± 0.18	236.97 ± 18.09	8.39 ± 3.92	45.94 ± 20.37		K02291//15-cis-phytoene synthase	phytoene synthase, chloroplastic-like	*DnPHYSC*
110092990	15.73 ± 1.61	5 ± 0.02	101.83 ± 4.84	0.09 ± 0	10.51 ± 8.52		K02437//glycine cleavage system H protein	glycine cleavage system H protein, mitochondrial-like	*DnGCSHM*

110112671	1.46 ± 0.18	1.6 ± 0.03	1.02 ± 0	21.64 ± 1.08	1.24 ± 0.31		K02469//DNA gyrase subunit A	uncharacterized protein LOC110112671	*DnUNP3*
110104311	92.72 ± 4.88	120.9 ± 1.16	976.24 ± 17.57	43.39 ± 8.01	72.98 ± 36.85		K02641//ferredoxin--NADP+ reductase	ferredoxin--NADP reductase, leaf isozyme, chloroplastic-like	*DnFDRLIC*
110093665	338.38 ± 7.3	675.21 ± 29.5	1549.1 ± 91.32	30.5 ± 6.57	219.88 ± 45.72		K02693//photosystem I subunit IV	photosystem I reaction center subunit IV A, chloroplastic-like	*DnPIRCS4AC*
110109061	491.83 ± 57.67	2226.7 ± 167.04	3681.06 ± 588.3	372.24 ± 294.62	131.64 ± 43.06		K02698//photosystem I subunit X	photosystem I reaction center subunit psaK, chloroplastic	*DnPSAKC*
110103433	475.26 ± 33.93	911.76 ± 67.26	2656.04 ± 172.2	18.59 ± 9.87	203.93 ± 68.71		K02699//photosystem I subunit XI	photosystem I reaction center subunit XI, chloroplastic	*DnPIRCS6C*
110113815	390.35 ± 10.51	1261.15 ± 37.2	2424.95 ± 111.0	110.34 ± 36.35	392.3 ± 155.98		K02716//photosystem II oxygen-evolving enhancer protein 1	oxygen-evolving enhancer protein 1, chloroplastic-like	*DnOEEP1C*
110099725	458.2 ± 11.12	1151.28 ± 50.1	2660.87 ± 15.86	191.85 ± 36.48	414.13 ± 109.77		K02717//photosystem II oxygen-evolving enhancer protein 2	oxygen-evolving enhancer protein 2, chloroplastic-like	*DnOEEP2C*
110099403	436.37 ± 33.1	949.34 ± 35.84	2276.78 ± 43.87	118.82 ± 39.38	259.47 ± 11.61		K02721//photosystem II PsbW protein	photosystem II reaction center W protein, chloroplastic	*DnPIIRCWC*
110105728	2.04 ± 0.22	4.04 ± 0.74	20.42 ± 0.33	2.08 ± 0.96	1.24 ± 0.54		K02836//peptide chain release factor 2	peptide chain release factor PrfB3, chloroplastic	*DnPRFB3C*
110092451	186.2 ± 0.06	236.86 ± 4.35	66.35 ± 0.2	116.69 ± 9.11	194.52 ± 7.67		K02875//large subunit ribosomal protein L14e	stem-specific protein TSJT1-like	*DnTSJT1L*
110097503	1.9 ± 0.17	0.08 ± 0.01	0.04 ± 0.03	0.13 ± 0.04	0.11 ± 0.01		K02941//large subunit ribosomal protein LP0	O fucosyltransferase 19	*DnOFT19*
110099788	4.82 ± 0.01	0.55 ± 0.02	0.28 ± 0.15	0.06 ± 0.05	0 ± 0		K03006//DNA-directed RNA polymerase II subunit RPB1	sugar carrier protein C-like	*DnSCPCL*
110106620	678.82 ± 39.73	28.01 ± 1.87	267.64 ± 149.26	68.75 ± 23.32	1198.22 ± 73.95		K03006//DNA-directed RNA polymerase II subunit RPB1	protein SRC2 like	*DnSRC2L*
110094623	319.15 ± 59.67	382.98 ± 9.58	91.69 ± 50.58	409.67 ± 119.11	250.89 ± 39.45		K03036//26S proteasome regulatory subunit N6	pollen-specific protein C13-like	*DnPSPC13L*
110092561	13.91 ± 0.05	24.12 ± 0.8	166.9 ± 26.04	18.39 ± 12.59	25.08 ± 0.66	Sigma70-like	K03093//RNA polymerase sigma factor	RNA polymerase sigma factor sigE, chloroplastic/mitochondrial	*DnSigE*
110102036	64.04 ± 3.89	114.83 ± 5.45	141.74 ± 9.2	176.86 ± 46.83	230.24 ± 32.5		K03301//ATP:ADP antiporter, AAA family	plastidic ATP/ADP-transporter-like	*DnPAATL*
110096905	6.22 ± 0.03	1.34 ± 0	112.38 ± 2.2	5.4 ± 4.29	9.46 ± 5.1		K03320//ammonium transporter, Amt family	ammonium transporter 1 member 2-like	*DnAMMT1.2L*
110100877	19.79 ± 0.77	204.16 ± 4.32	39.36 ± 1.54	14.07 ± 4.23	17.88 ± 3.11		K03377//N-acetylneuraminate 9-O-acetyltransferase	protein REDUCED WALL ACETYLATION 4-like	*DnRWA4L*
110104983	1005.45 ± 88.13	1937.47 ± 82.82	11359.26 ± 670.79	515.34 ± 69.53	1451.65 ± 292.33		K03541//photosystem II 10kDa protein	photosystem II 10 kDa polypeptide, chloroplastic	*DnPTS2-10C*
110097644	163.81 ± 5.08	309.08 ± 4.74	1955.69 ± 68.22	161.33 ± 89.05	266.46 ± 154.34		K03542//photosystem II 22kDa protein	photosystem II 22 kDa protein, chloroplastic	*DnPTS2-22C*
110096977	0.12 ± 0.01	0 ± 0	0 ± 0	0 ± 0	0 ± 0		K03549//KUP system potassium uptake protein	potassium transporter 26 like, partial	*DnPOTT26L*
110095230	14.81 ± 0.71	1.91 ± 0.27	4.4 ± 1.03	173.6 ± 140.99	7.41 ± 1.16		K03549//KUP system potassium uptake protein	potassium transporter 5	*DnPOT5L*
110095143	2.49 ± 0.13	0.09 ± 0.02	0.02 ± 0.02	0.3 ± 0.17	0 ± 0		K03695//ATP-dependent Clp protease ATP-binding subunit ClpB	aluminum-activated malate transporter 10-like	*DnAAMT10L*
110095858	74.86 ± 9.4	16.06 ± 0.22	830.35 ± 71.91	93 ± 43.08	135.1 ± 14.6		K03781//catalase	catalase isozyme 1	*DnCATI1*
110114360	1093.35 ± 211.0	1179.39 ± 19.3	2388.23 ± 162.4	259.44 ± 154.04	3706.73 ± 764.2		K03781//catalase	catalase isozyme A	*DnCATIA*
110107179	37.12 ± 8.07	494.07 ± 7.88	0 ± 0	0.09 ± 0.07	0.58 ± 0.47		K03860//phosphatidylinositol glycan, class Q	gibberellin-regulated protein 12-like	*DnGRP12L*
110112142	18.3 ± 2.49	110.68 ± 0.92	1.24 ± 0.76	7.14 ± 2.16	10.11 ± 4.83		K03860//phosphatidylinositol glycan, class Q	peamaclein-like	*DnPEAL*
110115456	565.23 ± 35.95	96.14 ± 5.36	7.11 ± 0.22	22.62 ± 14.43	9.53 ± 5.73		K04077//chaperonin GroEL+ko03018//RNA degradation	NDR1/HIN1-like protein 13	*DnNDR1/HIN1-L13*
110095910	12.7 ± 3.02	91.58 ± 4.72	0 ± 0	0.5 ± 0.29	21.5 ± 6.02		K04120//ent-copalyl diphosphate synthase	bifunctional levopimaradiene synthase, chloroplastic-like	*DnBLSC*
110111963	18.61 ± 1.44	0.02 ± 0.02	0 ± 0	0.03 ± 0.02	0.57 ± 0.16		K04124//gibberellin 3beta-dioxygenase	gibberellin 3-beta-dioxygenase 1-like	*DnGBD1L*
110110825	11.83 ± 6.01	0.03 ± 0.02	0.03 ± 0.02	0.74 ± 0.56	147.3 ± 15.66		K04125//gibberellin 2beta-dioxygenase	2-oxoglutarate-dependent dioxygenase AOP1	*DnAOP1*
110113126	22.69 ± 0.15	0.09 ± 0.02	0.15 ± 0.12	0.07 ± 0.02	0 ± 0		K04125//gibberellin 2beta-dioxygenase	gibberellin 2-beta-dioxygenase 2-like	*DnGBD2L*
110111510	907.25 ± 274.89	6.12 ± 0.08	3217.71 ± 444.2	19.09 ± 15.07	429.5 ± 61.98		K04125//gibberellin 2beta-dioxygenase	probable 2-oxoglutarate-dependent dioxygenase AOP1.2	*DnAOP1.2L*
110095100	167.34 ± 15.53	95.05 ± 0.25	78.37 ± 8.62	225.51 ± 80.16	155.71 ± 32.87	bZIP	K04450//cyclic AMP-dependent transcription factor ATF-2	basic leucine zipper 9	*DnbZIP9*
110105179	78.79 ± 6.38	14.71 ± 1.33	1.93 ± 0.33	4.38 ± 2.73	7.56 ± 0.42		K04508//transducin (beta)-like 1	FK506-binding protein 4-like	*DnFBP4L*

110101955	44.54 ± 4.78	292.81 ± 19.59	1.75 ± 0.38	26.24 ± 12.46	1.14 ± 0.31		K04733//interleukin-1 receptor-associated kinase 4	heavy metal-associated isoprenylated plant protein 7-like	*DnHMAIP7L*
110105180	7.65 ± 0.09	21.55 ± 1.14	17.94 ± 1.99	0.02 ± 0.01	241.5 ± 44.07		K04733//interleukin-1 receptor-associated kinase 4	heavy metal-associated isoprenylated plant protein 7-like	*DnHMIPP7L*
110098068	2.85 ± 0.32	0 ± 0	0 ± 0	0 ± 0	0 ± 0	PLATZ	K04733//interleukin-1 receptor-associated kinase 4	PLATZ transcription factor	*DnPLATZL1*
110107111	26.6 ± 1.56	1.41 ± 0.03	0.04 ± 0.03	0.19 ± 0.09	0.1 ± 0.08	PLATZ	K04733//interleukin-1 receptor-associated kinase 4	PLATZ transcription factor	*DnPLATZL2*
110111863	0 ± 0	1 ± 0.08	0 ± 0	0 ± 0	0 ± 0	bHLH	K04733//interleukin-1 receptor-associated kinase 4	transcription factor bHLH137	*DnbHLH137*
110095482	23.99 ± 2.4	85.27 ± 0.24	29.1 ± 4.83	175.53 ± 27.53	11.67 ± 0.46	bHLH	K04733//interleukin-1 receptor-associated kinase 4	transcription factor bHLH79-like	*DnbHLH79L*
110093171	118.21 ± 12.17	101.15 ± 2.11	307.97 ± 28.1	32.47 ± 6.32	117.48 ± 11.55	PLATZ	K04733//interleukin-1 receptor-associated kinase 4	uncharacterized protein LOC110093171	*DnPLATZL3*
110107433	0.37 ± 0.04	0.56 ± 0.19	1.1 ± 0.24	0.19 ± 0.01	70.58 ± 7.46	bHLH	K04733//interleukin-1 receptor-associated kinase 4	uncharacterized protein LOC110107433	*DnbHLH-L1*
110116483	95.78 ± 41.75	38.99 ± 0.2	77.25 ± 14.68	233.08 ± 136.37	71.29 ± 14.19	PLATZ	K04733//interleukin-1 receptor-associated kinase 4	uncharacterized protein LOC110116483	*DnPLATZL4*
110099214	0 ± 0	2.77 ± 0.02	0.09 ± 0.02	0 ± 0	0.04 ± 0.03	bHLH	K04733//interleukin-1 receptor-associated kinase 4	transcription factor BEE 1 like	*DnbHLH-BEE1L*
110112486	3.04 ± 0.03	0.02 ± 0.02	0 ± 0	0 ± 0	0 ± 0		K05282//gibberellin-44 dioxygenase	gibberellin 20 oxidase 1 D like	*DnG20O1DL1*
110107704	20.32 ± 1.78	1.89 ± 0.18	1.48 ± 0.5	0.22 ± 0.04	2.13 ± 0.09		K05282//gibberellin-44 dioxygenase	gibberellin 20 oxidase 1-D-like	*DnG20O1DL2*
110098715	1.01 ± 0.04	0 ± 0	0 ± 0	0 ± 0	0 ± 0		K05282//gibberellin-44 dioxygenase	gibberellin 20 oxidase 2-like	*DnG20O2L*
114578346	4.23 ± 0.11	0.27 ± 0.07	0.02 ± 0.01	0 ± 0	0.02 ± 0.01		K05349//beta-glucosidase	beta-D-glucoside glucohydrolase Provisional	*DnPRK15098*
110095762	58.6 ± 5.2	0.5 ± 0.05	2.15 ± 1.35	0.42 ± 0.04	4.52 ± 3.23		K05349//beta-glucosidase	Periplasmic beta-glucosidase and related glycosidases	*DnBGLX1*
110107248	30.3 ± 7.61	1.09 ± 0.09	0.01 ± 0.01	0.39 ± 0.26	175 ± 2.97		K05349//beta-glucosidase	Periplasmic beta-glucosidase and related glycosidases	*DnBGLX2*
110092217	63.72 ± 12.12	482.6 ± 13.02	734.43 ± 130.44	36.87 ± 9.44	1391.58 ± 427.06		K05350//beta-glucosidase	beta glucosidase 1 like	*DnBGS1L*
110095269	111.33 ± 2.6	5.38 ± 0.5	11.79 ± 2.49	0.62 ± 0	0.41 ± 0.07		K05350//beta-glucosidase	beta glucosidase 18	*DnBGS18*
110098119	1.13 ± 0.04	6.34 ± 0.27	0.08 ± 0.02	0.2 ± 0.16	0.08 ± 0.04		K05391//cyclic nucleotide gated channel, plant	cyclic nucleotide-gated ion channel 2	*DnCNGIC2.1*
110107311	64.58 ± 7.68	230.89 ± 6.52	208.34 ± 24.94	71.66 ± 18.06	44.94 ± 8.66		K05391//cyclic nucleotide gated channel, plant	cyclic nucleotide-gated ion channel 4-like	*DnCNGIC4L*
110101066	16.96 ± 2.38	138.43 ± 2.47	179.33 ± 32.33	156.42 ± 109.18	157.18 ± 29.85		K05391//cyclic nucleotide gated channel, plant	cyclic nucleotide gated ion channel 2	*DnCNGIC2.2*
110113121	40.02 ± 2.69	19.63 ± 0.79	401.19 ± 12.81	6.36 ± 2.54	23.13 ± 13.07		K05605//3-hydroxyisobutyryl-CoA hydrolase	chloroplast stem-loop binding protein of 41 kDa b, chloroplastic	*DnSLBP41C*
110104737	8.56 ± 0.91	20.03 ± 0.87	34.24 ± 5.29	150.61 ± 101.65	44.21 ± 11.12		K05658//ATP-binding cassette, subfamily B (MDR/TAP), member 1	ABC transporter B family member 2-like	*DnABCB2L*
110093959	5.83 ± 0.35	40.91 ± 1.35	2.99 ± 0.09	1.75 ± 1.43	0.32 ± 0.12		K05681//ATP-binding cassette, subfamily G (WHITE), member 2	ABC transporter G family member 5	*DnABCG5*
110092299	73.03 ± 1.4	1.62 ± 0.23	4.99 ± 0.57	7.16 ± 5.84	0.27 ± 0		K05681//ATP-binding cassette, subfamily G (WHITE), member 2	ABC transporter G family member 6-like	*DnABCG6L*
110095160	1.24 ± 0.14	6.37 ± 0.72	3.55 ± 0.16	383.25 ± 216.87	3.39 ± 0.24		K05681//ATP-binding cassette, subfamily G (WHITE), member 2	ABC transporter G family member 36	*DnABCG36*
110094128	8.7 ± 0.56	0.86 ± 0.05	0.59 ± 0.11	1.28 ± 0.05	0.37 ± 0.06		K05681//ATP-binding cassette, subfamily G (WHITE), member 2	ABC transporter G family member 39	*DnABCG39*
110096814	0.22 ± 0.02	60.09 ± 0.57	219.53 ± 38.5	407.63 ± 71.53	28.02 ± 13.52		K05681//ATP-binding cassette, subfamily G (WHITE), member 2	ABC transporter G family member 11-like	*DnABCG11L1*
110114867	32.86 ± 7.13	19.56 ± 0.04	328.75 ± 105.58	0.18 ± 0.13	45.83 ± 26.67		K05681//ATP-binding cassette, subfamily G (WHITE), member 2	ABC transporter G family member 11-like	*DnABCG11L2*
110111202	102.84 ± 7.23	3.72 ± 0.1	4.62 ± 0.3	159.82 ± 77.84	0.25 ± 0.05		K05681//ATP-binding cassette, subfamily G (WHITE), member 2	ABC transporter G family member 20-like	*DnABCG20L*
110102296	2.54 ± 0.17	12.14 ± 0.98	0.02 ± 0.01	0.45 ± 0.15	0.06 ± 0.02		K05681//ATP-binding cassette, subfamily G (WHITE), member 2	ABC transporter G family member 8-like	*DnABCG8L*

110096322	0.13 ± 0.02	0.01 ± 0	0.03 ± 0.01	231.73 ± 182.15	0.1 ± 0	K05681//ATP-binding cassette, subfamily G (WHITE), member 2	ABC transporter G family member 35	*DnABCG35*
110104216	19.61 ± 1.68	0.61 ± 0.05	0.02 ± 0.02	0.01 ± 0	1.11 ± 0.27	K05681//ATP-binding cassette, subfamily G (WHITE), member 2	ABC transporter G family member 44	*DnABCG44*
110108201	2.19 ± 0.34	2.77 ± 0.14	35.19 ± 0.63	0.87 ± 0	1.08 ± 0.04	K05681//ATP-binding cassette, subfamily G (WHITE), member 2	ABC transporter G family member 22	*DnABCG22*
110097332	9.89 ± 0.25	0.03 ± 0.01	0.02 ± 0.01	0.16 ± 0.02	0.63 ± 0.32	K05681//ATP-binding cassette, subfamily G (WHITE), member 2	ABC transporter G family member 44 like	*DnABCG44L*
110094207	341 ± 19.59	7.2 ± 0.29	0 ± 0	0.53 ± 0.33	0.29 ± 0.15	K05747//Wiskott-Aldrich syndrome protein	auxin-binding protein ABP19a-like	*DnABP19a*
110100273	0.02 ± 0.01	0 ± 0	125.69 ± 5.27	0 ± 0	1.15 ± 0.85	K05747//Wiskott-Aldrich syndrome protein+ko04144//Endocytosis	phosphoglucosamine mutase family protein	*DnPGMFL*
110110991	515.86 ± 17.87	599.72 ± 8.32	342.14 ± 20.93	745.56 ± 151.43	573.74 ± 21.38	K05863//solute carrier family 25 (mitochondrial adenine nucleotide translocator), member 4/5/6/31	ADP,ATP carrier protein 1, mitochondrial-like	*DnAACP1ML*
110096378	333.62 ± 4.15	60.98 ± 2.02	70.74 ± 0.18	75.24 ± 20.4	50.38 ± 3.6	K05894//12-oxophytodienoic acid reductase	putative 12 oxophytodienoate reductase 4	*DnODR4*
110103452	8.71 ± 0.46	0.14 ± 0.04	0.04 ± 0.02	0.09 ± 0.06	0.31 ± 0.09	K05909//laccase	laccase	*DnLAC*
110114163	587.35 ± 51.26	40.21 ± 0.54	0.79 ± 0.4	14.4 ± 11.56	0.63 ± 0.33	K05909//laccase	laccase-22-like	*DnLAC22L*
110101212	57.41 ± 5.23	3.33 ± 0.12	1.08 ± 0.12	1.09 ± 0.29	1.27 ± 0.08	K05909//laccase	laccase-3-like	*DnLAC3L2*
110107385	91.45 ± 5.2	10.47 ± 0.42	0.75 ± 0.22	1.36 ± 0.25	0.82 ± 0.12	K05909//laccase	laccase-3-like	*DnLAC3L1*
110103466	1.62 ± 0.07	0.03 ± 0	0 ± 0	0.03 ± 0.02	0 ± 0	K05909//laccase	Multicopper oxidase with three cupredoxin domains	*DnSUFI*
110105667	494.08 ± 125.12	33.71 ± 1.56	2123.32 ± 31.27	202.69 ± 61.26	64 ± 13.56	K06124//phosphoethanolamine/phosphocholine phosphatase	thiamine phosphate phosphatase like protein	*DnTPPLP*
110114188	0.09 ± 0.01	0.06 ± 0	0.06 ± 0.01	1.18 ± 0.03	0.06 ± 0.02	K06180//23S rRNA pseudouridine1911/1915/1917 synthase	magnesium transporter	*DnMAGTL*
110096339	254.91 ± 6.04	671.8 ± 15.4	696.71 ± 55.54	578.61 ± 1.54	494.54 ± 69.63	K06185//ATP-binding cassette, subfamily F, member 2	ABC transporter F family member 1	*DnABCF1*
110111327	32.74 ± 2.5	79.88 ± 3.03	254.76 ± 13.44	38.78 ± 1.5	61.64 ± 8.01	K06185//ATP-binding cassette, subfamily F, member 2	ABC transporter F family member 5	*DnABCF5*
110098495	118.78 ± 4.84	20.42 ± 1.24	21.28 ± 1.22	11.59 ± 1.12	14.45 ± 2.09	K06573//solute carrier family 4 (anion exchanger), member 1	boron transporter 1-like	*DnBORT1L*
110114363	18.51 ± 1.02	2.28 ± 0	2.24 ± 0.38	0.28 ± 0.13	0.39 ± 0.01	K06901//putative MFS transporter, AGZA family, xanthine/uracil permease	adenine/guanine permease AZG2	*DnAZG2*
110116543	97.58 ± 26.41	118.98 ± 3.46	190.67 ± 136.01	167.58 ± 49.06	49.06 ± 4.61	K07213//copper chaperone	copper transport protein ATX1	*DnATX1L2*
110108538	4.33 ± 3.53	0 ± 0	0 ± 0	540.29 ± 122.42	1.03 ± 0.84	K07213//copper chaperone	copper transport protein ATX1-like	*DnATX1L1*
110097786	279.13 ± 22.14	395.68 ± 2.01	164.36 ± 30.17	258.78 ± 140.83	14.62 ± 2.37	K07300//Ca2+:H+ antiporter	vacuolar cation/proton exchanger 1a	*DnVCPE1A*
110108018	0.04 ± 0.03	0.43 ± 0	0.1 ± 0.02	0.01 ± 0.01	0.01 ± 0.01	K07385//1,8-cineole synthase	terpene synthase 10-like	*DnTPS10L*
110108280	49.09 ± 2.38	0.13 ± 0.02	0.31 ± 0.25	0.42 ± 0.34	0.33 ± 0.27	K07407//alpha-galactosidase	alpha galactosidase like	*DnAGTSL*
110113345	3.46 ± 0.71	2.34 ± 0.19	236.11 ± 15.58	8.97 ± 5.53	8.59 ± 1.19	K07409//cytochrome P450 family 1 subfamily A polypeptide 2	cytochrome P450 89A2-like	*DnCYP89A2L*
110093950	0 ± 0	0 ± 0	0 ± 0	0.07 ± 0	0 ± 0	K07478//putative ATPase	Integrase core domain	*DnRVE*
110092666	0.81 ± 0	0 ± 0	0 ± 0	0 ± 0	0.03 ± 0.02	K07893//Ras-related protein Rab-6A	purine permease 1-like	*DnPPE1L*
110099931	5.33 ± 0.53	0 ± 0	0 ± 0	0 ± 0	0 ± 0	K07893//Ras-related protein Rab-6A	purine permease 3 like	*DnPPE3L1*
110099929	2.93 ± 0.31	0 ± 0	0 ± 0	0 ± 0	0 ± 0	K07893//Ras-related protein Rab-6A	purine permease 3-like	*DnPPE3L2*
110097241	88.79 ± 1.91	14.25 ± 0.08	0.44 ± 0.1	13.2 ± 0.15	1.67 ± 0.34	K07964//heparanase	heparanase-like protein 3	*DnHLP3*
110109620	107.08 ± 8.65	84.08 ± 1.64	58.8 ± 2.98	152.81 ± 13.37	59.76 ± 7.25	K08065//nuclear transcription Y subunit beta	nuclear transcription factor Y subunit B-1-like	*DnNFYB1L2*
110101477	1.08 ± 0.07	0.27 ± 0.07	0.09 ± 0.07	0.23 ± 0.08	0.2 ± 0.08	K08065//nuclear transcription Y subunit beta	nuclear transcription factor Y subunit B-1-like	*DnNFYB1L1*
110105256	12.99 ± 1.18	1.07 ± 0.08	73.57 ± 3.72	0.09 ± 0.07	1.14 ± 0.11	K08066//nuclear transcription factor Y, gamma	nuclear transcription factor Y subunit C-4-like	*DnNFYC4L*
110105441	6.36 ± 0.34	0.61 ± 0.25	0 ± 0	0 ± 0	0.3 ± 0.05	K08081//tropinone reductase I	transcriptional regulator RABBIT EARS	*DnC2H2-RE*
110111059	13.25 ± 1.12	0 ± 0	0.04 ± 0.03	0 ± 0	1.6 ± 0	K08081//tropinone reductase I	transcriptional regulator SUPERMAN-like	*DnC2H2-SUPERMAN*
110112133	3.44 ± 0.37	15.06 ± 0.04	1.27 ± 0.09	0.66 ± 0.27	0.14 ± 0.02	K08081//tropinone reductase I	zinc finger protein 7-like	*DnZFP7L*

110113794	110.47 ± 0.42	22.44 ± 0.8	20.34 ± 4.73	25.42 ± 8.42	24.7 ± 4.05		K08145//MFS transporter, SP family, solute carrier family 2 (facilitated glucose transporter), member 8	sugar transporter ERD6 like 16	*DnERD6L16*
110107595	46.13 ± 2.05	58.79 ± 2.02	182.83 ± 31.78	28.56 ± 10.46	96.81 ± 13.13		K08150//MFS transporter, SP family, solute carrier family 2 (myo-inositol transporter), member 13	polyol transporter 5-like	*DnPOOT5L*
110106488	158.02 ± 2.27	10.23 ± 0.19	45.63 ± 4.8	33.27 ± 25.34	76.29 ± 2.79		K08150//MFS transporter, SP family, solute carrier family 2 (myo-inositol transporter), member 13	sugar transport protein MST3	*DnMST3L*
110098884	129.69 ± 18.11	4.42 ± 0.3	178.31 ± 83.35	5.76 ± 0.48	404.76 ± 67.24		K08176//MFS transporter, PHS family, inorganic phosphate transporter	inorganic phosphate transporter 1 4	*DnIPT1.4L2*
110099893	86.59 ± 11.46	8.6 ± 0.25	371.22 ± 12.26	11.81 ± 1.41	558.44 ± 27.51		K08176//MFS transporter, PHS family, inorganic phosphate transporter	probable inorganic phosphate transporter 1 4	*DnIPT1.4L1*
110112226	1.21 ± 0.17	1.51 ± 0	1.97 ± 0.29	9.64 ± 0.22	0.6 ± 0.04		K08200//MFS transporter, OCT family, solute carrier family 22 (organic cation transporter), member 3	organic cation/carnitine transporter 3 like	*DnOCCT3L*
110107033	16.08 ± 1.48	2.39 ± 0.17	0.15 ± 0.1	0.13 ± 0.07	0.2 ± 0.08		K08202//MFS transporter, OCT family, solute carrier family 22 (organic cation transporter), member 4/5	organic cation/carnitine transporter 4-like	*DnOCCT4L*
110109539	12.11 ± 0.29	64.18 ± 1.12	1.55 ± 1.22	3.37 ± 0	1.9 ± 0.36		K08235//xyloglucan:xyloglucosyl transferase	xyloglucan endotransglucosylase/hydrolase protein 8	*DnXTH8*
110094498	153.36 ± 10.68	30.53 ± 0.78	19.01 ± 5.42	28.97 ± 5.13	15.46 ± 2.71		K08237//hydroquinone glucosyltransferase	hydroquinone glucosyltransferase-like	*DnHGTL*
110094607	2.18 ± 0.1	0.09 ± 0.07	0.03 ± 0.02	0 ± 0	0 ± 0		K08237//hydroquinone glucosyltransferase	UDP-glycosyltransferase 72B3-like	*DnUGT72B3L*
110102853	69.01 ± 2.32	87.04 ± 1.18	184.13 ± 4.02	56.32 ± 1.94	98.16 ± 13.71	MYB	K08869//aarF domain-containing kinase	transcription factor MYBS3-like	*DnMYBS3L*
110109513	455.94 ± 20.2	1187.33 ± 72.24	2256.38 ± 112.1[illegible]	30.8 ± 9.38	183.8 ± 43.07		K08901//photosystem II oxygen-evolving enhancer protein 3	oxygen-evolving enhancer protein 3, chloroplastic-like	*DnOEEP3C*
110111495	288.56 ± 15.3	1125.83 ± 54.77	1586.24 ± 221.5[illegible]	73.94 ± 40.55	357.13 ± 125.97		K08905//photosystem I subunit V	photosystem I reaction center subunit V, chloroplastic	*DnPIRCS5C*
110099194	378.15 ± 39.51	858.45 ± 41.91	1568.64 ± 242.0[illegible]	0.11 ± 0.04	18.91 ± 7.47		K08912//light-harvesting complex II chlorophyll a/b binding protein 1	chlorophyll a-b binding protein of LHCII type 1-like	*DnLHCII1L2*
110098704	2635.67 ± 198.2[illegible]	4092.79 ± 170.7[illegible]	5637.89 ± 415.6[illegible]	240.76 ± 174.4	622.56 ± 220.91		K08912//light-harvesting complex II chlorophyll a/b binding protein 1	chlorophyll a-b binding protein of LHCII type 1-like	*DnLHCII1L*
110114429	736.43 ± 87.96	2630.33 ± 162.7[illegible]	773.11 ± 182.2	1.07 ± 0.42	49.05 ± 34.71		K08912//light-harvesting complex II chlorophyll a/b binding protein 1	chlorophyll a-b binding protein of LHCII type 1-like	*DnLHCII1L1*
110104670	1478.62 ± 124.4[illegible]	4809.27 ± 297.3[illegible]	4774.05 ± 369.9[illegible]	148.67 ± 112.76	296.06 ± 119.11		K08913//light-harvesting complex II chlorophyll a/b binding protein 2	chlorophyll a-b binding protein 5, chloroplastic	*DnCAB5C*
110113255	369.41 ± 29.6	829.29 ± 60.82	1593.91 ± 107.2[illegible]	66.89 ± 46.31	70.95 ± 37.38		K08916//light-harvesting complex II chlorophyll a/b binding protein 5	chlorophyll a-b binding protein CP26, chloroplastic	*DnCP26C*
110113543	45.64 ± 6.03	63.1 ± 0.19	144.25 ± 17.43	46.91 ± 11.46	374.03 ± 155.65		K09060//plant G-box-binding factor	bZIP transcription factor 11-like	*DnbZIP11L*
110104334	294.32 ± 24.19	497.33 ± 26.2	212.75 ± 4.48	161.86 ± 5.25	210.99 ± 34.19	CSD	K09250//cellular nucleic acid-binding protein	glycine rich protein 2	*DnCSD-GRP2*
110093052	120.68 ± 2.04	103.91 ± 0.53	213.77 ± 17.73	15.61 ± 5.82	24.31 ± 13.86	MADS	K09260//MADS-box transcription enhancer factor 2A	MADS box transcription factor 50 like	*DnMADS50L*
110108289	94.55 ± 0.84	151.05 ± 3.73	180.34 ± 31.19	7.81 ± 6.25	0.12 ± 0.05	MADS	K09260//MADS-box transcription enhancer factor 2A	MADS-box protein SVP-like	*DnMADS-SVPL*
110114689	0.03 ± 0.02	0.43 ± 0.06	1.92 ± 0.31	144.34 ± 27.28	560.79 ± 82.22	MADS	K09264//MADS-box transcription factor, plant	agamous-like MADS-box protein AGL9 homolog	*DnMADS-AGL9H*
110116738	0.12 ± 0	0.51 ± 0.09	0.02 ± 0.02	95.64 ± 5.46	299.65 ± 18.37	MADS	K09264//MADS-box transcription factor, plant	agamous-like MADS-box protein AGL9 homolog	*DnMADS-AGL9*
110098137	0.17 ± 0.03	0.08 ± 0.02	0.08 ± 0.02	0.2 ± 0	36.08 ± 3.07	MADS	K09264//MADS-box transcription factor, plant	floral homeotic protein AGAMOUS-like	*DnMADS-AGAMOUS*
110105915	9.62 ± 1.03	13.39 ± 0.64	49.22 ± 4.61	0.22 ± 0.16	357.86 ± 201.21	MADS	K09264//MADS-box transcription factor, plant	floral homeotic protein AGAMOUS-like	*DnMADS-AGAMOUSL*
110097381	0.11 ± 0	0.32 ± 0.09	0.12 ± 0.07	189.8 ± 8.05	89.67 ± 10.83	MADS	K09264//MADS-box transcription factor, plant	MADS-box protein CMB1-like	*DnMADS-CMB1L*

110096227	0.08 ± 0.02	0.18 ± 0.09	0 ± 0	292.65 ± 55.96	0.13 ± 0.03	MADS	K09264//MADS-box transcription factor, plant	MADS-box transcription factor 16-like	*DnMADS16L3*
110103364	12.91 ± 2.51	3.43 ± 0.06	2.31 ± 0.3	325.47 ± 148.42	9 ± 2.67	MADS	K09264//MADS-box transcription factor, plant	MADS-box transcription factor 16-like	*DnMADS16L1*
110093040	0 ± 0	5.2 ± 0.18	6.37 ± 0.6	314.35 ± 83.61	0.05 ± 0	MADS	K09264//MADS-box transcription factor, plant	MADS-box transcription factor 16-like	*DnMADS16L2*
110097524	14.75 ± 1.39	48.82 ± 0.33	17.98 ± 0.05	813.94 ± 34.99	66.67 ± 4.21	MADS	K09264//MADS-box transcription factor, plant	MADS-box transcription factor 2	*DnMADS2L*
110112358	14.51 ± 0.53	0.01 ± 0	0.02 ± 0	1.01 ± 0.1	0.01 ± 0.01	MADS	K09264//MADS-box transcription factor, plant	MADS-box transcription factor 23-like	*DnMADS23L*
110111596	0.11 ± 0.03	0.08 ± 0.07	0.03 ± 0.02	44.52 ± 1.13	393.04 ± 40.95	MADS	K09264//MADS-box transcription factor, plant	MADS-box transcription factor 6	*DnMADS6*
110095336	0 ± 0	0.04 ± 0.03	0 ± 0	194.18 ± 112.64	19.69 ± 16.01	MADS	K09264//MADS-box transcription factor, plant	MADS-box transcription factor 6-like	*DnMADS6L*
110096755	0.14 ± 0.02	12.81 ± 0.5	1.43 ± 0.06	1.4 ± 0.43	0.54 ± 0.44	MADS	K09264//MADS-box transcription factor, plant	truncated transcription factor CAULIFLOWER A-like	*DnMADS-CAL*
110094210	0.16 ± 0.01	1.36 ± 0.28	0.47 ± 0.04	0.19 ± 0.15	20.72 ± 0.44	AP2-EREBP	K09285//AP2-like factor, ANT lineage	AP2-like ethylene-responsive transcription factor AIL1	*DnAP2-EREBPL-AIL1*
110107204	0.33 ± 0.04	3.59 ± 0.11	0.01 ± 0.01	0.08 ± 0.06	0.69 ± 0.27	AP2-EREBP	K09285//AP2-like factor, ANT lineage	AP2-like ethylene-responsive transcription factor ANT	*DnAP2-EREBP-ANT*
110092113	6.73 ± 0.75	0.06 ± 0.02	0 ± 0	0 ± 0	0 ± 0	AP2-EREBP	K09285//AP2-like factor, ANT lineage	AP2-like ethylene-responsive transcription factor At1g79700	*DnAP2-EREBPL1*
110093714	0.54 ± 0.03	0.12 ± 0.03	0.04 ± 0.03	0 ± 0	0.05 ± 0.04	AP2-EREBP	K09285//AP2-like factor, ANT lineage	AP2-like ethylene-responsive transcription factor At1g79700	*DnAP2-EREBPL2*
110111961	7.79 ± 0.99	45.25 ± 0.16	5.5 ± 0.1	7.05 ± 2.17	6.59 ± 1.13	AP2-EREBP	K09285//AP2-like factor, ANT lineage	AP2-like ethylene-responsive transcription factor At2g41710	*DnAP2-EREBPL3*
110099389	0 ± 0	1.31 ± 0.02	0 ± 0	0.05 ± 0.04	0 ± 0	AP2-EREBP	K09286//EREBP-like factor	dehydration-responsive element-binding protein 2E-like	*DnAP2-EREBP-DRE2E*
110108187	183.95 ± 16.12	164.01 ± 0.05	58.49 ± 0.02	83.51 ± 10.35	253.57 ± 47.58	AP2-EREBP	K09286//EREBP-like factor	ethylene-responsive transcription factor 1-like	*DnAP2-EREBP-ERF1L2*
110092963	250.16 ± 3.56	152.09 ± 0.9	348.14 ± 29.55	156.21 ± 17.46	806.7 ± 135.24	AP2-EREBP	K09286//EREBP-like factor	ethylene-responsive transcription factor 1-like	*DnAP2-EREBP-ERF1L1*
110108252	165.37 ± 14.99	37.14 ± 1.29	58.34 ± 27	57.38 ± 12.81	220.17 ± 65.46	AP2-EREBP	K09286//EREBP-like factor	ethylene-responsive transcription factor 3-like	*DnAP2-EREBP-ERF3L*
110096335	780.3 ± 14.35	40.42 ± 1.33	143.74 ± 27.77	25.34 ± 6.83	598.84 ± 36.22	AP2-EREBP	K09286//EREBP-like factor	ethylene-responsive transcription factor 4-like	*DnAP2-EREBP-ERF4L*
110109181	585.82 ± 31.69	16.14 ± 0.64	41.76 ± 22.5	48.21 ± 27.14	275.53 ± 23.4	AP2-EREBP	K09286//EREBP-like factor	ethylene-responsive transcription factor 9-like	*DnAP2-EREBP-ERF9L*
110109222	14.85 ± 1.33	0.05 ± 0.04	0.81 ± 0.14	0.1 ± 0.08	0.19 ± 0.03	AP2-EREBP	K09286//EREBP-like factor	ethylene-responsive transcription factor ERF013-like	*DnAP2-EREBP-ERF013*
110097214	23.54 ± 0.85	0 ± 0	2.02 ± 1.58	0.33 ± 0.04	5.47 ± 0.43	AP2-EREBP	K09286//EREBP-like factor	ethylene-responsive transcription factor ERF018-like	*DnAP2-EREBP-ERF018*
110100414	7.91 ± 0.38	0.32 ± 0.09	0.11 ± 0	0.53 ± 0.43	0.12 ± 0.09	AP2-EREBP	K09286//EREBP-like factor	ethylene-responsive transcription factor ERF026-like	*DnAP2-EREBP-ERF026*
110114791	0.04 ± 0.03	0 ± 0	0 ± 0	0.02 ± 0.01	0.93 ± 0.04	AP2-EREBP	K09286//EREBP-like factor	ethylene-responsive transcription factor ESR2-like	*DnAP2-EREBP-ESR2L*
110103007	157.1 ± 4.65	34.97 ± 0.63	79.64 ± 39.76	35.94 ± 8.61	542.84 ± 29.19	AP2-EREBP	K09286//EREBP-like factor	ethylene-responsive transcription factor RAP2-13-like	*DnAP2-EREBP-RAP2.1*[illegible]
110110379	246.14 ± 18.3	17.62 ± 1.17	158.37 ± 47.24	21.81 ± 13.11	119.42 ± 13.44	AP2-EREBP	K09286//EREBP-like factor	ethylene-responsive transcription factor RAP2-4-like	*DnAP2-EREBP-RAP2.4*
110112594	685.68 ± 21.59	90.02 ± 3.72	245.23 ± 31.66	38.77 ± 17.47	1204.21 ± 137.04	AP2-EREBP	K09286//EREBP-like factor	ethylene-responsive transcription factor RAP2-4-like	*DnAP2-EREBP-RAP2.4*[illegible]
110103508	43.6 ± 12.4	0.38 ± 0.05	64.01 ± 51.01	0.45 ± 0.27	235.79 ± 7.69	AP2-EREBP	K09286//EREBP-like factor	dehydration responsive element binding protein 1G	*DnAP2-EREBP-DRE1G*
110112064	181.95 ± 30.03	2.9 ± 0.05	89.32 ± 60.81	2.21 ± 1.22	482.85 ± 7.23	AP2-EREBP	K09286//EREBP-like factor	dehydration responsive element binding protein 1C like	*DnAP2-EREBP-DRE1C*[illegible]
110102213	137.3 ± 4.78	55.08 ± 2.61	67.43 ± 42.16	2.18 ± 1.21	360.36 ± 46.45	AP2-EREBP	K09287//RAV-like factor	AP2/ERF and B3 domain-containing protein Os05g0549800-like	*DnAP2-EREBPL*
110111670	131.59 ± 11.35	205.49 ± 0.47	11.4 ± 2.49	234.41 ± 43.43	18.16 ± 4.97	E2F-DP	K09391//transcription factor E2F7/8	E2F transcription factor-like E2FE	*DnE2FEL*
110091897	0.04 ± 0.03	7.04 ± 0.45	0 ± 0	0.06 ± 0.02	0.53 ± 0.43	HSF	K09419//heat shock transcription factor, other eukaryote	heat stress transcription factor B-4b-like	*DnHSFB-4bL*
110093640	27.11 ± 1.47	89.98 ± 4.96	286.04 ± 0.21	1.59 ± 0.65	9.38 ± 3.41	MYB	K09422//transcription factor MYB, plant	MYB-like transcription factor ETC1	*DnMYB-ETC1*
110105078	0.06 ± 0.05	0 ± 0	0 ± 0	946.38 ± 84.37	0.05 ± 0.04	MYB	K09422//transcription factor MYB, plant	myb-related protein 305-like	*DnMYB305L2*
110092042	77.01 ± 4.96	2.79 ± 0.13	0.12 ± 0.03	1.02 ± 0.77	3.84 ± 0.55	MYB	K09422//transcription factor MYB, plant	myb-related protein 305-like	*DnMYB305L1*
110098979	105.57 ± 2.88	0.4 ± 0.22	4.5 ± 0.27	0.19 ± 0.02	3.88 ± 0.64	MYB	K09422//transcription factor MYB, plant	myb-related protein 308-like	*DnMYB308L1*
110099624	25.98 ± 1.71	100.98 ± 0.62	249.15 ± 40.56	117.36 ± 65.52	19.39 ± 2.75	MYB	K09422//transcription factor MYB, plant	myb-related protein 308-like	*DnMYB308L2*
110107258	0.05 ± 0.04	0.74 ± 0.03	0 ± 0	0 ± 0	0.04 ± 0.03	MYB	K09422//transcription factor MYB, plant	myb-related protein MYBAS1-like	*DnMYBAS1L*
110099916	3.06 ± 1.07	25.08 ± 0.78	2.2 ± 0.44	1.81 ± 0.75	1.42 ± 0.13	MYB	K09422//transcription factor MYB, plant	myb-related protein MYBAS2-like	*DnMYBAS2L*
110105411	27.22 ± 0.1	0.16 ± 0	0 ± 0	0.03 ± 0	0.31 ± 0	MYB	K09422//transcription factor MYB, plant	myb-related protein Zm1-like	*DnMYB-ZM1L2*
110092846	13.17 ± 1.2	2.31 ± 0.14	0.34 ± 0.16	0.41 ± 0.31	0 ± 0	MYB	K09422//transcription factor MYB, plant	myb-related protein Zm1-like	*DnMYB-ZM1L1*
110114678	37.4 ± 4.56	30.7 ± 1.85	43.29 ± 4.14	310.91 ± 205.34	19.36 ± 0.37	MYB	K09422//transcription factor MYB, plant	myb-related protein Zm38-like	*DnMYB-ZM38L*
110104863	82.83 ± 3.49	15.44 ± 0.04	1.77 ± 0.53	2.63 ± 2.07	0.29 ± 0.11	MYB	K09422//transcription factor MYB, plant	protein ODORANT1-like	*DnMYB-ODORANT1*

110104286	38.12 ± 1.25	0.12 ± 0.1	0 ± 0	0 ± 0	0 ± 0	MYB	K09422//transcription factor MYB, plant	transcription factor LAF1-like	*DnMYB-LAF1L*
110114027	9.53 ± 0.36	0 ± 0	0.03 ± 0.02	0 ± 0	0 ± 0	MYB	K09422//transcription factor MYB, plant	transcription factor MYB36	*DnMYB36L3*
110098041	21.52 ± 1.48	3.85 ± 0.06	0.02 ± 0.02	3.35 ± 1.82	0.05 ± 0	MYB	K09422//transcription factor MYB, plant	transcription factor MYB36-like	*DnMYB36L1*
110110329	22.31 ± 1.25	0 ± 0	0.03 ± 0.02	0 ± 0	0.9 ± 0.24	MYB	K09422//transcription factor MYB, plant	transcription factor MYB36-like	*DnMYB36L2*
110096046	29.17 ± 2.27	0.02 ± 0.02	0.04 ± 0.03	0.16 ± 0.11	0 ± 0	MYB	K09422//transcription factor MYB, plant	transcription factor MYB39-like	*DnMYB39L*
110111576	19.71 ± 1.38	2.74 ± 0.04	1.51 ± 0.33	1.12 ± 0.71	0.12 ± 0.07	MYB	K09422//transcription factor MYB, plant	transcription factor MYB52-like	*DnMYB52L2*
110107293	23.9 ± 0.14	4.33 ± 0.47	1.04 ± 0.16	0.29 ± 0.13	0.46 ± 0.11	MYB	K09422//transcription factor MYB, plant	transcription factor MYB52-like	*DnMYB52L1*
110112067	8.36 ± 0.71	0.25 ± 0.05	0 ± 0	0 ± 0	0.29 ± 0	MYB	K09422//transcription factor MYB, plant	transcription factor MYB56-like	*DnMYB56L*
110106196	18.4 ± 0.82	1.81 ± 0.24	1.02 ± 0.12	0.37 ± 0.21	3.22 ± 0.9	MYB	K09422//transcription factor MYB, plant	transcription factor RAX2-like	*DnMYB-RAX2L3*
110092191	16.8 ± 0.93	1.37 ± 0.04	1.22 ± 0.84	0.13 ± 0.03	2.03 ± 0.68	MYB	K09422//transcription factor MYB, plant	transcription factor RAX2-like	*DnMYB-RAX2L1*
110096293	31.38 ± 0.36	0.99 ± 0	0.38 ± 0.07	0.05 ± 0.04	1.48 ± 0.31	MYB	K09422//transcription factor MYB, plant	transcription factor RAX2-like	*DnMYB-RAX2L2*
110107270	13.56 ± 0.58	2.36 ± 0.18	0 ± 0	0 ± 0	0.2 ± 0.07	MYB	K09422//transcription factor MYB, plant	transcription factor RAX3-like	*DnMYB-RAX3L*
110104906	0 ± 0	0.05 ± 0.04	0.09 ± 0	523.91 ± 360.19	0.15 ± 0.12	MYB	K09422//transcription factor MYB, plant	myb related protein 305 like	*DnMYB305L3*
110104416	5.44 ± 0.4	0.03 ± 0.02	0 ± 0	0 ± 0	0 ± 0	MYB	K09422//transcription factor MYB, plant	transcription factor MYB111 like	*DnMYB111L*
110100217	3.82 ± 0.62	11.38 ± 0.1	64.24 ± 1.11	0.34 ± 0.13	8.98 ± 0.91		K09753//cinnamoyl-CoA reductase	cinnamoyl CoA reductase 1 like	*DnCCR1L*
110101632	417.83 ± 30.32	42.48 ± 0.62	7.95 ± 0.14	42.95 ± 10.87	15.81 ± 5.6		K09754//5-O-(4-coumaroyl)-D-quinate 3'-monooxygenase	cytochrome P450 98A2	*DnCYP98A2*
110097029	13.9 ± 0.47	25.1 ± 0.6	157.55 ± 14.1	16.02 ± 7.38	47.28 ± 16.98	FHA	K09838//zeaxanthin epoxidase	zeaxanthin epoxidase, chloroplastic	*DnFHA-ZE*
110097017	3.39 ± 0.04	0.06 ± 0.01	0.16 ± 0.01	0.04 ± 0.03	0.52 ± 0.15		K09843//(+)-abscisic acid 8'-hydroxylase	abscisic acid 8' hydroxylase 3	*DnABAH3*
110112069	2.23 ± 0.35	15.1 ± 0.44	0.3 ± 0.05	1.03 ± 0.45	98.66 ± 8.59		K09843//(+)-abscisic acid 8'-hydroxylase	abscisic acid 8'-hydroxylase 3-like	*DnABAH3L*
110105075	1160.15 ± 336.89	473.46 ± 17.26	852.9 ± 99.45	668.88 ± 370.57	1260 ± 36.83		K09872//aquaporin PIP	aquaporin PIP2-7-like	*DnPIP2-7L*
110101105	234.92 ± 32.16	131.3 ± 9.5	12.63 ± 3.98	47.51 ± 21.11	10.66 ± 4.94		K09873//aquaporin TIP	probable aquaporin TIP2-1	*DnTIP2-1L*
110115126	71.71 ± 4.53	0.19 ± 0.11	0.6 ± 0.07	0.02 ± 0.01	0 ± 0		K09874//aquaporin NIP	aquaporin NIP1-1-like	*DnNIP1-1L*
110094629	3.09 ± 0.09	0.07 ± 0.05	0.27 ± 0.08	0.06 ± 0.05	0.02 ± 0.02		K10206//LL-diaminopimelate aminotransferase	aminotransferase ALD1 homolog	*DnALD1H*
110093398	0.34 ± 0.1	2.14 ± 0.16	0.33 ± 0.13	17.5 ± 1.54	0.47 ± 0.21		K10251//17beta-estradiol 17-dehydrogenase / very-long-chain 3-oxoacyl-CoA reductase	very-long-chain 3-oxoacyl-CoA reductase 1-like	*DnVOCR1L*
110115852	102.46 ± 7.02	0.94 ± 0.06	3.03 ± 0.43	0.88 ± 0.39	0.18 ± 0.02		K10258//very-long-chain enoyl-CoA reductase	very-long-chain enoyl-CoA reductase-like	*DnVECRL*
110113181	76.04 ± 11.6	346.99 ± 16.84	208.95 ± 30.41	5.95 ± 4.79	1678.48 ± 677.4.	TAZ	K10523//speckle-type POZ protein	BTB/POZ and TAZ domain-containing protein 1-like	*DnTAZ1L*
110109032	19.05 ± 1.45	29.38 ± 1.6	0.16 ± 0	154.79 ± 8.23	0.18 ± 0.04		K10526//OPC-8:0 CoA ligase 1	4-coumarate--CoA ligase-like 7	*Dn4CL7*
110098475	0.82 ± 0.01	7.58 ± 0.14	0 ± 0	0.04 ± 0.03	0.23 ± 0.06		K10526//OPC-8:0 CoA ligase 1	putative 4 coumarate CoA ligase like 8	*Dn4CL8L*
110107863	42.78 ± 3.97	3.26 ± 0.09	1 ± 0.12	2.05 ± 0.85	0.86 ± 0.24		K10691//E3 ubiquitin-protein ligase UBR4	GDSL esterase/lipase At1g74460-like	*DnGDSLL3*
110098873	83.83 ± 3.74	79.46 ± 0.71	341.93 ± 19.84	9.84 ± 1.83	584.98 ± 185.93		K10752//histone-binding protein RBBP4	heavy metal-associated isoprenylated plant protein 23-like	*DnHMAIP23L*
110116586	1.5 ± 0.04	0.05 ± 0.01	0.02 ± 0.01	0.01 ± 0.01	0 ± 0		K10760//adenylate dimethylallyltransferase (cytokinin synthase)	adenylate isopentenyltransferase-like	*DnAIPTL*
110111300	135.05 ± 18.58	2591.63 ± 74.5	8.38 ± 1.11	808.56 ± 658.87	2.87 ± 0.13		K10781//fatty acyl-ACP thioesterase B	MLP-like protein 423	*DnMLP423*
110102359	9.2 ± 0.56	0.92 ± 0.05	0 ± 0	0.01 ± 0.01	0 ± 0		K10781//fatty acyl-ACP thioesterase B	palmitoyl-acyl carrier protein thioesterase, chloroplastic-like	*DnPCPTC*
110113891	0.36 ± 0.09	2.72 ± 0.16	0.14 ± 0.07	0.09 ± 0.02	0.08 ± 0.06	bHLH	K10863//aprataxin	transcription factor bHLH87-like	*DnbHLH87L*
110110399	3.13 ± 0.13	0.43 ± 0.02	0.1 ± 0.04	0.02 ± 0.01	0.12 ± 0	bHLH	K10863//aprataxin	putative transcription factor bHLH086	*DnbHLH086L*
110096275	299.2 ± 10.19	45.27 ± 0.89	0.02 ± 0.02	14.74 ± 11.07	0.3 ± 0.02		K10999//cellulose synthase A	cellulose synthase A catalytic subunit 7 [UDP-forming]	*DnCLSA*
114580705	0 ± 0	2.71 ± 0.03	0.03 ± 0.02	0 ± 0	0 ± 0		K11153//retinol dehydrogenase 12	short chain dehydrogenase TIC 32, chloroplastic like	*DnTIC32CL1*
110107237	2.13 ± 0.28	99.16 ± 1.78	23.01 ± 0.02	7.12 ± 1.94	3.96 ± 0.58		K11153//retinol dehydrogenase 12	short-chain dehydrogenase TIC 32, chloroplastic-like	*DnTIC32CL2*
114580165	2.51 ± 0.16	0 ± 0	0 ± 0	0 ± 0	0 ± 0		K11294//nucleolin	cysteine rich repeat secretory protein 55 like	*DnCRRSP55*
110097286	14.17 ± 0.21	87.1 ± 0.22	692.62 ± 34.06	6.15 ± 4.41	57.21 ± 16.31		K11517//(S)-2-hydroxy-acid oxidase	peroxisomal (S)-2-hydroxy-acid oxidase GLO1	*DnGLO1*
110114880	204.46 ± 5.85	188.88 ± 4.52	225.47 ± 29.26	47.76 ± 6.96	457.37 ± 95.88		K11721//bromodomain-containing protein 3	transcription factor GTE2-like	*DnGTE2L*
110108319	0.08 ± 0	0.59 ± 0.05	0.06 ± 0.05	0.02 ± 0.01	3.18 ± 0.28		K12126//phytochrome-interacting factor 3	transcription factor bHLH85 like	*DnbHLH85L*
110093354	11.84 ± 0.09	6.1 ± 0.09	8.83 ± 1.4	273.3 ± 52.86	4.16 ± 0.33	MYB	K12133//MYB-related transcription factor LHY	protein REVEILLE 8-like	*DnMYB-REVEILLE8L*
110110597	8.05 ± 0.74	2.6 ± 0.04	11.74 ± 3	186.19 ± 120.92	8.74 ± 5.38	MYB	K12133//MYB-related transcription factor LHY	protein LHY-like	*DnMYB-LHYL2*

110100892	3.82 ± 0.03	7.21 ± 0.47	8.36 ± 0.29	324.17 ± 260.12	1.17 ± 0.04	MYB	K12133//MYB-related transcription factor LHY	protein LHY-like	*DnMYB-LHYL1*
110097602	25.78 ± 1.25	231.8 ± 10.35	596.02 ± 101.2	217.51 ± 173.61	242.95 ± 106.79	MYB	K12133//MYB-related transcription factor LHY	protein CCA1	*DnMYB-CCA1*
110113387	4.58 ± 0.2	52.25 ± 0.91	312.07 ± 67.8	29.54 ± 22.31	48.16 ± 27.94	C2C2-CO-like	K12135//zinc finger protein CONSTANS	zinc finger protein CONSTANS-LIKE 2-like	*DnC2C2-CONSTANS2L*
110114028	77.67 ± 6.19	93.69 ± 3.93	467.9 ± 76.66	88.97 ± 11.28	347.31 ± 88.43	C2C2-CO-like	K12135//zinc finger protein CONSTANS	zinc finger protein CONSTANS-LIKE 3-like	*DnC2C2-CONSTANS3L*
110095225	83.18 ± 1.45	110.08 ± 5.7	259.34 ± 46.27	41.75 ± 8.58	77.01 ± 8.81	C2C2-CO-like	K12135//zinc finger protein CONSTANS	zinc finger protein CONSTANS-LIKE 5-like	*DnC2C2-CL5*
110112517	5.56 ± 0.02	0.06 ± 0.03	0.03 ± 0	0.05 ± 0.02	0 ± 0		K12309//beta-galactosidase	beta-galactosidase 13	*DnBGLS13*
110092037	4.27 ± 0.37	0.31 ± 0.01	0.09 ± 0.01	0.06 ± 0.04	0.3 ± 0.04		K12309//beta-galactosidase	beta-galactosidase 7-like	*DnBGLS7L*
110098051	6.28 ± 0.59	5.75 ± 0.13	37.56 ± 3.25	4.76 ± 3.03	3.76 ± 2.11		K12589//exosome complex component RRP42	Leucine carboxyl methyltransferase	*DnLCM*
110092298	25.37 ± 0.33	0.87 ± 0.1	5.5 ± 1.09	0.78 ± 0.31	5.85 ± 1.62		K12614//ATP-dependent RNA helicase DDX6/DHH1	glycine-rich protein A3-like	*DnGRP-A3*
110108667	1.93 ± 0.21	51.9 ± 1.88	185.56 ± 10.27	49.09 ± 37.99	642.47 ± 482.29		K12741//heterogeneous nuclear ribonucleoprotein A1/A3	heterogeneous nuclear ribonucleoprotein A3-like	*DnHNRP-A3L*
110099511	0.07 ± 0.03	47.04 ± 4.4	0 ± 0	0.03 ± 0.02	0.66 ± 0.54		K12742//isoprene synthase	alpha-terpineol synthase, chloroplastic-like	*DnATSCL*
110115059	7.77 ± 0.66	0.24 ± 0.19	0 ± 0	0 ± 0	0 ± 0	WRKY	K12835//ATP-dependent RNA helicase DDX42	probable WRKY transcription factor 28	*DnWRKY28*
110095975	26.97 ± 2.6	0.71 ± 0.04	0.66 ± 0.45	0.25 ± 0.13	0.27 ± 0.05	WRKY	K12835//ATP-dependent RNA helicase DDX42	probable WRKY transcription factor 43	*DnWRKY43L*
110100473	82.67 ± 2.68	141.18 ± 1.85	124.94 ± 13.94	115.28 ± 0.37	74.58 ± 11.92	C3H	K12836//splicing factor U2AF 35 kDa subunit	splicing factor U2af small subunit B-like	*DnC3H-U2AFB*
110098587	15.12 ± 1.36	0.31 ± 0.25	0.27 ± 0.04	1.11 ± 0.9	0 ± 0		K13065//shikimate O-hydroxycinnamoyltransferase	hydroxycinnamoyltransferase 1-like	*DnHCT1L1*
110113109	105.29 ± 6.12	7.57 ± 0.33	1.05 ± 0.34	8.96 ± 6.25	6.43 ± 0.87		K13065//shikimate O-hydroxycinnamoyltransferase	hydroxycinnamoyltransferase 1-like	*DnHCT1L2*
110113317	3.6 ± 0.67	60.62 ± 4.16	3.66 ± 0.31	1688.18 ± 1371.[illegible]	3.36 ± 1.2		K13070//momilactone-A synthase	short-chain dehydrogenase reductase 3b-like	*DnSCDR3BL*
110101283	34.18 ± 3.93	0.35 ± 0.04	0 ± 0	0.03 ± 0.02	0.17 ± 0.09		K13070//momilactone-A synthase	short-chain dehydrogenase reductase 3b-like	*DnSCDR3B*
110113312	10.39 ± 0.43	241.88 ± 14.57	30.2 ± 0.95	3.24 ± 2.54	52.59 ± 14.34		K13070//momilactone-A synthase	short-chain dehydrogenase reductase 3b-like	*DnSDR3BL*
110103653	42.17 ± 2.28	168.77 ± 1.37	0.58 ± 0.06	72.53 ± 33.48	12.89 ± 2.57		K13098//RNA-binding protein FUS	heavy metal-associated isoprenylated plant protein 32	*DnHMIPP32*
110112271	0.1 ± 0.05	5.38 ± 0.13	0 ± 0	0.02 ± 0.02	0.26 ± 0.18		K13260//isoflavone/4'-methoxyisoflavone 2'-hydroxylase	cytochrome P450 81D1-like	*DnCYP81D1L*
110112054	0.59 ± 0.18	84.59 ± 6.31	1.01 ± 0.31	1190.54 ± 315.9[illegible]	4.25 ± 1.33		K13356//alcohol-forming fatty acyl-CoA reductase	alcohol-forming fatty acyl-CoA reductase-like	*DnAFFACRL*
110092925	20.47 ± 0.3	1.58 ± 0.17	2.03 ± 0.06	0.61 ± 0.01	1.77 ± 0.1		K13356//alcohol-forming fatty acyl-CoA reductase	fatty acyl-CoA reductase 4	*DnFAR4*
110098417	71.97 ± 4.46	395.58 ± 6.85	27.02 ± 15.91	5.81 ± 4.2	0.34 ± 0.02		K13366//polyamine oxidase	polyamine oxidase 1	*DnPAOD1*
110109586	1.02 ± 0.17	18.8 ± 1.74	0.32 ± 0.13	0.79 ± 0.43	0.21 ± 0.08	SBP	K13415//protein brassinosteroid insensitive 1	squamosa promoter-binding-like protein 14	*DnSBP14L*
110094435	298.1 ± 12.22	4.67 ± 0.17	36.45 ± 27.79	7.17 ± 2.16	243.32 ± 74.04	bHLH	K13422//transcription factor MYC2	transcription factor MYC2-like	*DnbHLH-MYC2*
110099934	106.98 ± 1.41	1.64 ± 0.11	36.72 ± 22.37	4.51 ± 0.16	400.88 ± 70.35	WRKY	K13424//WRKY transcription factor 33	probable WRKY transcription factor 31	*DnWRKY31*
110095613	127.73 ± 3.54	8.71 ± 0.15	28.53 ± 13.08	2.46 ± 0.76	372.18 ± 16.19	WRKY	K13424//WRKY transcription factor 33	WRKY transcription factor WRKY24-like	*DnWRKY24L*
114578713	39.68 ± 2.8	2.3 ± 0.32	7.04 ± 1.73	0 ± 0	8.66 ± 2.18	WRKY	K13425//WRKY transcription factor 22	probable WRKY transcription factor 65, partial	*DnWRKY65L*
110111979	225.85 ± 15.13	5.03 ± 0.04	104.48 ± 70.94	1.09 ± 0.37	299.38 ± 21.31	WRKY	K13459//disease resistance protein RPS2	probable WRKY transcription factor 40	*DnWRKY40L*
110108135	351.3 ± 2.37	162.25 ± 12.18	167.8 ± 77.48	85.53 ± 30.21	199.16 ± 24.51	Tify	K13464//jasmonate ZIM domain-containing protein	protein TIFY 10a-like	*DnTIFY10AL1*
110113635	120.23 ± 10.07	43.04 ± 3.63	111.42 ± 46.56	198.76 ± 103.52	46.37 ± 12.4	Tify	K13464//jasmonate ZIM domain-containing protein	protein TIFY 10a-like	*DnTify10AL2*
110097296	338.18 ± 10.59	0.14 ± 0.11	80.58 ± 65.79	2.49 ± 1.51	11.63 ± 3.04	Tify	K13464//jasmonate ZIM domain-containing protein	protein TIFY 5A-like	*DnTify5AL*
110099691	2.1 ± 0.14	0.01 ± 0.01	0.06 ± 0.01	0.12 ± 0.08	0.26 ± 0.07		K13496//UDP-glucosyl transferase 73C	anthocyanin 3'-O-beta-glucosyltransferase-like	*DnA3GTL2*
110099685	10.55 ± 0.59	0.24 ± 0.01	0.34 ± 0.27	0.45 ± 0.36	2.63 ± 0.02		K13496//UDP-glucosyl transferase 73C	UDP glycosyltransferase 73B5 like	*DnUGT73B5L*
110100348	36.77 ± 3.44	3.68 ± 0.3	1.65 ± 0.33	1.28 ± 0.22	2.27 ± 0.61		K13508//glycerol-3-phosphate acyltransferase	glycerol 3 phosphate acyltransferase 5	*DnGPAT5*

110111754	10.57 ± 0.94	0.2 ± 0.01	0.03 ± 0.02	0.1 ± 0.02	0.08 ± 0.01		K13508//glycerol-3-phosphate acyltransferase	glycerol-3-phosphate acyltransferase 3	*DnGPAT3*
110112921	42.36 ± 0.56	49.48 ± 2.31	35.76 ± 2.8	226.89 ± 48.81	23.8 ± 1.63		K13576//solute carrier family 38 (sodium-coupled neutral amino acid transporter), member 3	probable sodium-coupled neutral amino acid transporter 6	*DnANAAT6L*
110112100	6.95 ± 0.73	0.11 ± 0.02	0.02 ± 0.01	0.03 ± 0	0.02 ± 0.01		K13680//beta-mannan synthase	glucomannan 4 beta mannosyltransferase 1	*DnG4MT1*
110100481	0.4 ± 0.03	3.01 ± 0.3	0.38 ± 0.01	0.24 ± 0.11	0.26 ± 0.11		K13680//beta-mannan synthase	glucomannan 4-beta-mannosyltransferase 9-like	*DnGBMT9L*
110107853	6.18 ± 1.13	53.25 ± 2.65	1.33 ± 0.83	4.7 ± 3.8	1.55 ± 1.25		K13680//beta-mannan synthase	mannan synthase 11	*DnMNS11L1*
110108629	0.03 ± 0	134.3 ± 12.01	0.43 ± 0.16	0.33 ± 0.26	0.02 ± 0.01		K13680//beta-mannan synthase	mannan synthase 11	*DnMNS11L2*
110108624	6.24 ± 0.59	10.94 ± 0.16	3.66 ± 1.04	52.92 ± 1.89	5.45 ± 0.87		K13680//beta-mannan synthase	mannan synthase 9	*DnMNS9*
110106094	8.1 ± 1.42	45.66 ± 1.41	0.06 ± 0.03	0.03 ± 0.02	0.08 ± 0.06		K13699//abhydrolase domain-containing protein 5	lysophospholipase BODYGUARD 3	*DnBODYGUARD3*
110113243	6.22 ± 0.23	2.57 ± 0.17	1.99 ± 0.52	27.95 ± 0.03	6.74 ± 0.31		K13703//abhydrolase domain-containing protein 11	protein ABHD11	*DnABHD11*
110113169	129.92 ± 9.59	12.58 ± 0.53	205.82 ± 15.91	58.77 ± 1.11	176.2 ± 2.02		K13783//MFS transporter, OPA family, solute carrier family 37 (glycerol-3-phosphate transporter), member 1/2	putative glycerol-3-phosphate transporter 1	*DnG3PT1L*
110112787	545.25 ± 98.7	731.2 ± 1.51	811.27 ± 52.54	477.93 ± 6.05	2456.96 ± 232.43		K13783//MFS transporter, OPA family, solute carrier family 37 (glycerol-3-phosphate transporter), member 1/2	stem-specific protein TSJT1	*DnTSJT1*
110112538	0.21 ± 0.1	3.92 ± 0.22	0 ± 0	0.03 ± 0.02	0.14 ± 0.11	LOB	K13944//LOB domain-containing protein 16	LOB domain-containing protein 1	*DnLOB1L*
110099961	9.41 ± 0.63	0.92 ± 0.2	0 ± 0	0.02 ± 0.02	0 ± 0	LOB	K13945//LOB domain-containing protein 29	LOB domain-containing protein 29-like	*DnLOB29L*
110107606	0.08 ± 0	0 ± 0	0 ± 0	0 ± 0	0 ± 0	LOB	K13945//LOB domain-containing protein 29	LOB domain-containing protein 7-like	*DnLOB7L*
110100014	0.09 ± 0	0.03 ± 0	0.04 ± 0.02	0.46 ± 0.04	0.06 ± 0.02	FAR1	K13947//auxin efflux carrier family	auxin efflux carrier component 4 like	*DnFAR1-AECC4L*
110110074	312.54 ± 25.24	797.89 ± 9.33	1226.59 ± 205.8[illegible]	2370.28 ± 1637.[illegible]	1048.05 ± 227.19		K14190//GDP-L-galactose phosphorylase	GDP-L-galactose phosphorylase 1-like	*DnGGP1L*
110100274	8.92 ± 0.53	48.03 ± 0.56	11.65 ± 0.64	5.38 ± 3.98	4.52 ± 0.67		K14190//GDP-L-galactose phosphorylase	GDP-L-galactose phosphorylase 2-like	*DnGDPL-GP2L*
110109283	143.14 ± 8.59	140.74 ± 0.24	158.33 ± 17.72	135.58 ± 54.41	301.22 ± 99.78		K14207//solute carrier family 38 (sodium-coupled neutral amino acid transporter), member 2	amino acid transporter AVT6A like	*DnAVT6AL*
110109284	197.51 ± 0.99	119.28 ± 2.08	177.97 ± 2.25	178.54 ± 22.47	221.54 ± 34.57		K14207//solute carrier family 38 (sodium-coupled neutral amino acid transporter), member 2	amino acid transporter AVT6A	*DnAVT6A*
110095137	27.74 ± 5.53	42 ± 0.32	265.43 ± 45.56	39.91 ± 7.93	65.86 ± 1.47		K14209//solute carrier family 36 (proton-coupled amino acid transporter)	amino acid transporter ANTL2-like	*DnANTL2L*
110100062	8.01 ± 0.76	52.77 ± 0.81	1.06 ± 0.15	0.23 ± 0.07	2.65 ± 0.29		K14209//solute carrier family 36 (proton-coupled amino acid transporter)	lysine histidine transporter like 8	*DnLHT8L*
110113947	24.93 ± 1.76	29.37 ± 0.2	53.03 ± 7.94	62.71 ± 10.49	188.33 ± 35.59		K14209//solute carrier family 36 (proton-coupled amino acid transporter)	probable proline transporter 2	*DnPROT2L*
110109786	113.36 ± 15.55	3.3 ± 0.01	102.31 ± 32.41	30.35 ± 13.59	198.04 ± 50.53		K14209//solute carrier family 36 (proton-coupled amino acid transporter)	lysine histidine transporter 1	*DnLHT1*
110110983	51.13 ± 0.04	62.16 ± 0.47	862.4 ± 66.22	95.17 ± 29.41	46.08 ± 0.55		K14272//glutamate--glyoxylate aminotransferase	glutamate--glyoxylate aminotransferase 2-like	*DnGGAT2L*
110108718	0.07 ± 0.06	0.08 ± 0.06	0.23 ± 0	2.39 ± 0.11	0 ± 0		K14326//regulator of nonsense transcripts 1	ATP-binding cassette, subfamily B (MDR/TAP), member 1 LIKE	*DnABCB1L2*
114580577	0.37 ± 0	0.03 ± 0.02	0 ± 0	0 ± 0	0.03 ± 0.02		K14326//regulator of nonsense transcripts 1	ATP-binding cassette, subfamily B (MDR/TAP), member 1 LIKE	*DnABCB1L1*
110108723	28.82 ± 1.13	3.85 ± 0.1	20.35 ± 14.21	15.11 ± 7.07	254.96 ± 15.85	C2H2	K14412//alpha-1,4-fucosyltransferase	zinc finger protein STOP1 homolog	*DnC2H2-STOP1*
110102021	117.28 ± 16.95	19.43 ± 1	376.78 ± 29.41	58.7 ± 5.81	176 ± 44.72		K14430//phosphate transporter	SPX domain-containing protein 1-like	*DnSPX-CP1L*
110091793	26.35 ± 0.64	0.15 ± 0.05	0.72 ± 0.02	0.08 ± 0.01	0.66 ± 0.01		K14431//transcription factor TGA	bZIP transcription factor TGA10-like	*DnbZIP-TGA10L*
110113515	263.07 ± 7.56	298.35 ± 8.42	135.8 ± 4.76	204.39 ± 113.09	116.91 ± 1.4	bZIP	K14431//transcription factor TGA+ko04075//Plant hormone signal transduction	transcription factor TGA2.2-like	*DnbZIP-TGA2.2L*

110091922	41 ± 1.54	137.16 ± 0.75	316.3 ± 45.01	72.6 ± 57.71	82.55 ± 54.79		K14445//solute carrier family 13 (sodium-dependent dicarboxylate transporter), member 2/3/5	tonoplast dicarboxylate transporter	*DnTDCT*
110104921	26.02 ± 1.65	136.3 ± 4.07	13.1 ± 0.11	107.1 ± 8.85	121.19 ± 8.4	ARF	K14486//auxin response factor	auxin response factor 17-like	*DnARF17L*
110104630	60.71 ± 1.98	46.21 ± 2.71	43.25 ± 4.61	151.91 ± 69.02	121.49 ± 13.02	ARF	K14486//auxin response factor	auxin response factor 19-like	*DnARF19L*
110108738	0.96 ± 0.03	8.65 ± 0.26	0.2 ± 0	1.07 ± 0.69	0.19 ± 0.01	ARF	K14486//auxin response factor	auxin response factor 4-like	*DnARF4L*
110105349	26.19 ± 1.11	0 ± 0	0 ± 0	0.02 ± 0.02	0 ± 0	G2-like	K14491//two-component response regulator ARR-B family	probable transcription factor KAN4	*DnG2-KAN4*
110103356	9.72 ± 0.28	2.16 ± 0.22	0.03 ± 0.02	0.04 ± 0.03	0.77 ± 0.38	SRS	K14491//two-component response regulator ARR-B family	protein SHI RELATED SEQUENCE 1-like	*DnSRS1L*
110101861	11.63 ± 0.93	0 ± 0	0 ± 0	0 ± 0	0 ± 0	G2-like	K14491//two-component response regulator ARR-B family	putative Myb family transcription factor At1g14600	*DnG2-MYBL1*
110108751	48.78 ± 1.84	0.94 ± 0.13	3.58 ± 0.27	0.45 ± 0.28	5.99 ± 1.35	G2-like	K14491//two-component response regulator ARR-B family	putative Myb family transcription factor At1g14600	*DnG2-MYBL2*
110104535	19.97 ± 0.51	0.08 ± 0.02	3.24 ± 0.33	0.09 ± 0.02	0.08 ± 0.02	G2-like	K14491//two-component response regulator ARR-B family	uncharacterized protein	*DnG2-UNP1*
110101984	63.66 ± 32.5	20.67 ± 0.5	100.16 ± 70.26	15.8 ± 6.74	541.47 ± 332	GRAS	K14494//DELLA protein	chitin-inducible gibberellin-responsive protein 1-like	*DnGRAS-CGRP1L*
110095161	5.46 ± 0.55	6.48 ± 0.24	3.2 ± 0.24	6.87 ± 3.77	157.26 ± 1.04	GRAS	K14494//DELLA protein	DELLA protein SLR1-like	*DnGRAS-SLR1L*
110096572	1.74 ± 0.08	0 ± 0	0 ± 0	0.02 ± 0.02	0.04 ± 0.03	GRAS	K14494//DELLA protein	nodulation-signaling pathway 2 protein-like	*DnGRAS-NSP2L1*
110098490	3.25 ± 0.09	0.03 ± 0	0 ± 0	0 ± 0	0.04 ± 0.03	GRAS	K14494//DELLA protein	nodulation signaling pathway 2 protein like	*DnGRAS-NSP2L2*
110107124	60.49 ± 0.38	41.03 ± 0.25	44.52 ± 3.87	161.53 ± 73.93	91.34 ± 10	EIL	K14514//ethylene-insensitive protein 3	ETHYLENE INSENSITIVE 3-like 1 protein	*DnEIL3-1L1*
110113492	95.94 ± 7.54	102.41 ± 2.56	129.65 ± 15.69	157.74 ± 33.54	228.71 ± 40.45	EIL	K14514//ethylene-insensitive protein 3	ETHYLENE INSENSITIVE 3-like 1 protein	*DnEIL3-1L2*
110103673	100.41 ± 13.22	14.9 ± 0.53	127.93 ± 13.96	38.78 ± 23.54	304.1 ± 110.78		K14638//solute carrier family 15 (peptide/histidine transporter), member 3/4	protein NRT1/ PTR FAMILY 2.11-like	*DnNRT1/PTR2.11L*
110105212	23.91 ± 0.85	5.89 ± 0.36	2.65 ± 0.03	0.32 ± 0.24	0.72 ± 0.05		K14638//solute carrier family 15 (peptide/histidine transporter), member 3/4	protein NRT1/ PTR FAMILY 2.13-like	*DnNRT1-PTR2.13L*
110100095	174.77 ± 20.21	110.87 ± 4.49	177.18 ± 24.7	180.98 ± 33.04	12.72 ± 1.13		K14638//solute carrier family 15 (peptide/histidine transporter), member 3/4	protein NRT1/ PTR FAMILY 4.3-like	*DnNRT1/PTR4.3L*
110104019	7.69 ± 1.16	7.55 ± 0.29	9.17 ± 2.88	0.5 ± 0.08	46.3 ± 0.77		K14638//solute carrier family 15 (peptide/histidine transporter), member 3/4	protein NRT1/ PTR FAMILY 5.2-like	*DnNRT1-PTR5.2L*
110116352	43.25 ± 3.46	80.15 ± 4.12	281 ± 51	129.47 ± 39.32	98.91 ± 35.03		K14638//solute carrier family 15 (peptide/histidine transporter), member 3/4	protein NRT1/ PTR FAMILY 3.1	*DnNRT1/PTR3.1*
110107192	138.15 ± 13.21	121.23 ± 0.32	96.31 ± 2.64	161.73 ± 54.28	105.8 ± 11.98		K14686//solute carrier family 31 (copper transporter), member 1	copper transporter 5.1	*DnCOT5.1*
110093254	23.48 ± 0.35	1.46 ± 0.27	0 ± 0	1.65 ± 1.29	0.13 ± 0		K14686//solute carrier family 31 (copper transporter), member 1	copper transporter 6-like	*DnCOPT6L*
110105793	278.17 ± 4.89	374.69 ± 12.55	191.03 ± 5.21	207.89 ± 0.42	322.65 ± 13.64		K14689//solute carrier family 30 (zinc transporter), member 2	ricin B-like lectin EULS3	*DnEULS3*
110110895	4.48 ± 0.46	0 ± 0	0 ± 0	0 ± 0	0.02 ± 0.01		K14709//solute carrier family 39 (zinc transporter), member 1/2/3	leucine rich repeat extensin like protein 6	*DnLRR-EXT6L*
110101247	1.47 ± 0.42	22.34 ± 0.11	218.53 ± 3.63	22.69 ± 0.24	122.95 ± 14.93		K14709//solute carrier family 39 (zinc transporter), member 1/2/3	zinc transporter 8-like	*DnZINT8L*
110100914	100.12 ± 2.84	182.03 ± 3.08	185.59 ± 24.15	141.08 ± 11.12	274.6 ± 7.81		K14724//solute carrier family 9 (sodium/hydrogen exchanger), member 8	sodium/hydrogen exchanger 2-like	*DnSHE2L*
110109286	7.14 ± 0.21	8.66 ± 0.8	9.72 ± 0.7	75.4 ± 6.34	4.1 ± 1.02		K14759//isochorismate synthase / 2-succinyl-5-enolpyruvyl-6-hydroxy-3-cyclohexene-1-carboxylate synthase / 2-succinyl-6-hydroxy-2,4-cyclohexadiene-1-carboxylate synthase / O-succinylbenzoate synthase	protein PHYLLO, chloroplastic	*DnPHYLLOC*
110093084	221.56 ± 10.92	269.9 ± 8.94	108.4 ± 11.48	80.85 ± 1.56	148.04 ± 23.78	C3H	K14774//U3 small nucleolar RNA-associated protein 25	zinc finger CCCH domain-containing protein 14-like	*DnC3H14L*
110108200	200.76 ± 6.99	16.45 ± 0.09	38.8 ± 2.95	19.95 ± 4.98	77.58 ± 1.69	GRAS	K14777//ATP-dependent RNA helicase DDX47/RRP3	scarecrow-like protein 8	*DnGRAS-SLP8.2*

110094692	2.28 ± 0.1	0.49 ± 0.03	0.14 ± 0.03	0 ± 0	0.43 ± 0.04	GRAS	K14777//ATP-dependent RNA helicase DDX47/RRP3	scarecrow like protein 8	*DnGRAS-SLP8.1*
110104265	5.58 ± 0.4	0.43 ± 0.03	0.27 ± 0.11	0.35 ± 0.18	0.84 ± 0.22		K14794//ribosomal RNA-processing protein 12	protein NUCLEAR FUSION DEFECTIVE 4-like	*DnNFD4L*
110094876	0 ± 0	0 ± 0	0 ± 0	0.03 ± 0	0 ± 0		K14857//AdoMet-dependent rRNA methyltransferase SPB1	uncharacterized protein LOC110094876	*DnUNP1*
110115484	13.32 ± 0.2	0.02 ± 0.02	1 ± 0.04	0.39 ± 0.28	0.45 ± 0.11	bHLH	K14963//COMPASS component SWD3	transcription factor bHLH18-like	*DnbHLH18L*
110113094	21.28 ± 0.43	18.36 ± 0.6	24.75 ± 4.03	163.09 ± 1.9	14.16 ± 0.18		K14985//26-hydroxylase	geraniol 8-hydroxylase-like	*DnG8HL*
110100098	301.67 ± 5.17	263.91 ± 8.42	169.11 ± 18.15	176.54 ± 30.08	307.2 ± 13.2		K15040//voltage-dependent anion channel protein 2	mitochondrial outer membrane porin-like	*DnMOMPL*
110095729	160.64 ± 13.62	164.72 ± 3.77	116.34 ± 6.84	113.55 ± 2.54	125.18 ± 3.2		K15040//voltage-dependent anion channel protein 2	mitochondrial outer membrane protein porin 5	*DnMOMPP5*
110100120	245.68 ± 6.56	267.64 ± 10.37	132.57 ± 15.56	168.27 ± 7.45	322.58 ± 47.29		K15102//solute carrier family 25 (mitochondrial phosphate transporter), member 3	mitochondrial phosphate carrier protein 3, mitochondrial-like	*DnMPCP3L*
110094816	113.65 ± 4.69	139.53 ± 1.14	89.3 ± 0.24	92.25 ± 9.96	98.64 ± 8.27		K15104//solute carrier family 25 (mitochondrial oxoglutarate transporter), member 11	mitochondrial dicarboxylate/tricarboxylate transporter DTC-like	*DnDTCL*
110109147	204.65 ± 64.78	57.68 ± 1.4	49.89 ± 12.79	6.96 ± 3.86	112 ± 42.87		K15104//solute carrier family 25 (mitochondrial oxoglutarate transporter), member 11	mitochondrial uncoupling protein 5-like	*DnMUP5L2*
110116719	2028.99 ± 40.85	2605.98 ± 146.7	7254.51 ± 389.3	169.26 ± 136.12	6149.44 ± 78.51		K15104//solute carrier family 25 (mitochondrial oxoglutarate transporter), member 11	mitochondrial uncoupling protein 5-like	*DnMUP5L1*
110114337	802.09 ± 106.5	515.08 ± 10.39	1318.59 ± 314.6	244.06 ± 80.58	1779.45 ± 642.91		K15104//solute carrier family 25 (mitochondrial oxoglutarate transporter), member 11	mitochondrial uncoupling protein 5, partial	*DnMUP5P*
110092203	146.58 ± 9.83	132.71 ± 7.43	62.79 ± 2.89	100.77 ± 8.72	131.68 ± 5.95		K15280//solute carrier family 35, member C2	probable sugar phosphate/phosphate translocator At2g25520	*DnSPPTL*
110100362	173.24 ± 13.31	202.03 ± 1.81	34.04 ± 7.12	140.22 ± 20.18	127.71 ± 3.19		K15283//solute carrier family 35, member E1	phosphoenolpyruvate/phosphate translocator 2, chloroplastic	*DnPPPT2C*
110116070	106 ± 4.86	165.9 ± 4.12	257.33 ± 11.15	91.84 ± 20.01	213.64 ± 33.19		K15283//solute carrier family 35, member E1	triose phosphate/phosphate translocator, chloroplastic	*DnTPPTC*
110111477	69.94 ± 4.23	24.97 ± 1.15	140.02 ± 37.41	31.71 ± 10.25	200.02 ± 3.69		K15285//solute carrier family 35, member E3	protein LURP-one-related 15-like	*DnLOR15L*
110109113	244.32 ± 38.97	99.47 ± 2.25	38.15 ± 1.43	85.5 ± 7.43	45.66 ± 2.23		K15285//solute carrier family 35, member E3	Triose-phosphate Transporter family like	*DnTPTFL*
110097048	6.93 ± 0.57	0.07 ± 0.04	0.28 ± 0.07	0.25 ± 0.06	0.2 ± 0.1		K15333//tRNA guanosine-2'-O-methyltransferase	cucumisin-like	*DnCUCL1*
110097049	21.55 ± 0.2	0.02 ± 0.02	0.05 ± 0.04	0.18 ± 0.03	0.05 ± 0.04		K15333//tRNA guanosine-2'-O-methyltransferase	cucumisin-like	*DnCUCL2*
110111743	45.33 ± 0.08	0.09 ± 0	0.39 ± 0.29	0.35 ± 0.05	0.16 ± 0.13		K15333//tRNA guanosine-2'-O-methyltransferase	subtilisin-like protease SBT4.4	*DnSBT4.4*
110100367	1.19 ± 0.17	5.73 ± 0.16	0.5 ± 0.03	0.4 ± 0.17	0.73 ± 0.07		K15333//tRNA guanosine-2'-O-methyltransferase	subtilisin-like protease SBT5.6	*DnSBT5.6*
110111323	43.06 ± 3.83	81.54 ± 1.98	53.49 ± 8.23	217.1 ± 13.28	39.67 ± 1.89		K15378//solute carrier family 45, member 1/2/4	sucrose transport protein SUT1-like	*DnSUT1L*
110092000	178.2 ± 27.98	0.03 ± 0.02	0.03 ± 0.02	0.07 ± 0.05	0.41 ± 0.02		K15382//solute carrier family 50 (sugar transporter)	bidirectional sugar transporter SWEET14	*DnSWEET14L*
110108988	6.56 ± 0.67	5.8 ± 0	6.09 ± 1.11	122.38 ± 11.49	0.37 ± 0.18		K15382//solute carrier family 50 (sugar transporter)	bidirectional sugar transporter SWEET16-like	*DnSWEET16L*
110093234	1.48 ± 0.38	31.64 ± 0.67	7.73 ± 0.79	223.92 ± 62.48	6.97 ± 4.72		K15382//solute carrier family 50 (sugar transporter)	bidirectional sugar transporter SWEET17-like	*DnSWEET17L*
110097987	41.36 ± 0.98	92.09 ± 5.81	412.59 ± 79.36	138.65 ± 80.42	73.84 ± 7.46		K15382//solute carrier family 50 (sugar transporter)	bidirectional sugar transporter SWEET1a	*DnSWEET1a*

110112344	1.29 ± 0.54	37.54 ± 1.43	244.97 ± 45.38	14.16 ± 11.44	115.33 ± 32.66		K15382//solute carrier family 50 (sugar transporter)	bidirectional sugar transporter SWEET2a-like	*DnSWEET2aL*
110102273	4.93 ± 0.18	0.07 ± 0	0 ± 0	0 ± 0	0 ± 0		K15382//solute carrier family 50 (sugar transporter)	bidirectional sugar transporter SWEET4-like	*DnSWEET4L2*
110103260	154.52 ± 7.99	155.65 ± 7.22	18.76 ± 3.28	334.63 ± 50.08	543.7 ± 204.25		K15382//solute carrier family 50 (sugar transporter)	bidirectional sugar transporter SWEET4-like	*DnSWEET4L4*
110093459	26.66 ± 1.94	0 ± 0	0.04 ± 0.03	0 ± 0	0 ± 0		K15382//solute carrier family 50 (sugar transporter)	bidirectional sugar transporter SWEET6b-like	*DnSWEET6bL*
110116739	4.78 ± 0.08	0 ± 0	0 ± 0	0 ± 0	0 ± 0		K15382//solute carrier family 50 (sugar transporter)	bidirectional sugar transporter SWEET4 like	*DnSWEET4L1*
114581367	4.4 ± 0.18	0 ± 0	0 ± 0	0 ± 0	0 ± 0		K15382//solute carrier family 50 (sugar transporter)	bidirectional sugar transporter SWEET4 like	*DnSWEET4L3*
110111736	13.9 ± 3.67	272.92 ± 24.64	6.08 ± 1.1	12.18 ± 5.26	10.03 ± 4.05		K15397//3-ketoacyl-CoA synthase	3 ketoacyl CoA synthase 6 like	*DnKCS6L*
110116628	87.89 ± 2.13	542.96 ± 18.49	480.77 ± 86.81	2184.24 ± 168.1[illegible]	232.61 ± 155.6		K15397//3-ketoacyl-CoA synthase	3-ketoacyl-CoA synthase 10	*DnKCS10*
110113540	208.64 ± 6.03	33.9 ± 0.1	50.83 ± 3.57	24.61 ± 2.35	49.05 ± 15		K15397//3-ketoacyl-CoA synthase	3-ketoacyl-CoA synthase 11-like	*DnKCS11L*
110095646	44.3 ± 1.19	249.32 ± 5.09	2.51 ± 0.18	10.49 ± 8.46	4.24 ± 0.62		K15398//fatty acid omega-hydroxylase	cytochrome P450 86A8-like	*DnCYP86A8L*
110098662	0.68 ± 0.09	11.4 ± 0.1	0.2 ± 0.02	1.43 ± 0.35	0 ± 0		K15400//omega-hydroxypalmitate O-feruloyl transferase	omega-hydroxypalmitate O-feruloyl transferase	*DnHPFT*
110103888	288.54 ± 7.24	15.73 ± 1.28	10.87 ± 1.16	1.58 ± 0.73	4.03 ± 0.17		K15400//omega-hydroxypalmitate O-feruloyl transferase	omega-hydroxypalmitate O-feruloyl transferase-like	*DnHPFTL*
110095902	182.03 ± 13.11	2.33 ± 0.48	0 ± 0	0.19 ± 0.09	0.02 ± 0.01		K15400//omega-hydroxypalmitate O-feruloyl transferase	shikimate O-hydroxycinnamoyltransferase-like	*DnSHCTL*
110104795	31.61 ± 3.34	1.38 ± 0.03	0 ± 0	0 ± 0	0.03 ± 0.02		K15401//long-chain fatty acid omega-monooxygenase	cytochrome P450 86A1	*DnCYP86A1*
110108993	0.11 ± 0.01	38.11 ± 1.2	0 ± 0	1.76 ± 1.3	0.03 ± 0.02		K15402//fatty acid omega-hydroxylase	cytochrome P450 86B1	*DnCYP86B1*
110099638	99.51 ± 6.29	4.51 ± 0.13	1.25 ± 0.05	0.21 ± 0.14	0.58 ± 0.09		K15402//fatty acid omega-hydroxylase	cytochrome P450 86B1-like	*DnCYP86B1L1*
110109253	58.2 ± 6.57	1.84 ± 0.2	1.03 ± 0.11	0.12 ± 0.05	0.87 ± 0.46		K15402//fatty acid omega-hydroxylase	cytochrome P450 86B1-like	*DnCYP86B1L2*
110106751	5.42 ± 0.52	166.41 ± 2.71	1.44 ± 0.16	17.17 ± 13.29	3.63 ± 2.74		K15403//fatty acid omega-hydroxy dehydrogenase	protein HOTHEAD	*DnHOTHEAD*
110105062	8.06 ± 1.13	32.25 ± 0.01	4.91 ± 0.91	1.96 ± 0.49	0.09 ± 0.07		K15404//aldehyde decarbonylase	very long chain aldehyde decarbonylase GL1 1	*DnGL1-1*
110116345	10.14 ± 1.25	14.66 ± 0.82	34.09 ± 5.45	481.89 ± 23.71	12.51 ± 5.56		K15406//wax-ester synthase / diacylglycerol O-acyltransferase	O-acyltransferase WSD1-like	*DnWSD1L*
110097810	0 ± 0	133.19 ± 7.77	0.03 ± 0.02	0.05 ± 0.04	28.61 ± 3.32	HB	K15613//homeobox protein Meis1	homeobox protein knotted-1-like LET6	*DnHB-LET6*
110093666	45.51 ± 3.64	8.33 ± 0.04	4.03 ± 0.2	6.47 ± 1.54	8.34 ± 0.75		K15639//PHYB activation tagged suppressor 1	cytochrome P450 734A1-like	*DnCYP734A1L*
110107728	22.65 ± 1.54	1.81 ± 0.05	1.66 ± 0.41	2.39 ± 1.43	0.5 ± 0.1		K15718//linoleate 9S-lipoxygenase	linoleate 9S lipoxygenase 5	*DnL9SL5L1*
110096882	0.34 ± 0.07	1.38 ± 0.09	0.15 ± 0.02	0.03 ± 0	0.04 ± 0.01		K15718//linoleate 9S-lipoxygenase	linoleate 9S-lipoxygenase 5	*DnL9SL5L2*
110108011	316.69 ± 3.3	245.68 ± 5.48	14.88 ± 1.45	2243.06 ± 814.7[illegible]	43.42 ± 24.07		K15718//linoleate 9S-lipoxygenase	probable linoleate 9S-lipoxygenase 5	*DnL9L5L*
110097574	1.03 ± 0.07	35.45 ± 2.36	0.47 ± 0.01	3.99 ± 3.15	0.89 ± 0.03	zf-HD	K15777//4,5-DOPA dioxygenase extradiol	zinc finger homeodomain protein 8	*Dnzf-HD8*
110102832	3.94 ± 0.31	0 ± 0	0 ± 0	0 ± 0	0 ± 0	zf-HD	K15777//4,5-DOPA dioxygenase extradiol	zinc-finger homeodomain protein 11-like	*Dnzf-HD11L*
110098277	0 ± 0	0.28 ± 0.03	0 ± 0	0 ± 0	0 ± 0	zf-HD	K15777//4,5-DOPA dioxygenase extradiol	zinc-finger homeodomain protein 3-like	*Dnzf-HD3L*
110113071	0.33 ± 0	10.31 ± 0.07	2.11 ± 0.95	0.61 ± 0.14	1.46 ± 0.44	zf-HD	K15777//4,5-DOPA dioxygenase extradiol	zinc-finger homeodomain protein 5-like	*Dnzf-HD5L*
110109817	0.69 ± 0.05	64.98 ± 2.25	1.56 ± 1.19	0.06 ± 0.04	0.24 ± 0.2		K15803//(-)-germacrene D synthase	alpha humulene synthase like	*DnAHSL*
110094071	5.04 ± 0.67	0.03 ± 0.02	159.96 ± 37.95	2407.62 ± 1961.29	0.02 ± 0.01		K15803//(-)-germacrene D synthase	alpha humulene synthase like	*DnAHSL1*
110098806	31.46 ± 3.53	185.17 ± 0.74	0.88 ± 0.72	10.78 ± 8.8	0.13 ± 0.02		K15889//prenylcysteine alpha-carboxyl methylesterase	Pollen proteins Ole e I like	*DnPPO1L*
110112611	0.18 ± 0.07	30.74 ± 2.57	1.33 ± 0.31	1.75 ± 0.79	0.04 ± 0.03		K15889//prenylcysteine alpha-carboxyl methylesterase	pollen-specific protein-like At4g18596	*DnPSPL*
110112846	42.6 ± 3.11	46.96 ± 0.77	809.78 ± 43.76	17.87 ± 9.73	39.7 ± 22.5		K15893//glycerate dehydrogenase	glycerate dehydrogenase	*DnGDG*
110110392	5.16 ± 0.22	26.52 ± 0.67	0.35 ± 0.02	2.43 ± 1.83	1.45 ± 0.12		K15920//xylan 1,4-beta-xylosidase	beta-D-xylosidase 7	*DnBXS7*
110108332	5.91 ± 0.11	49.69 ± 0.12	0.04 ± 0	0.1 ± 0.08	0.02 ± 0.02		K16040//trans-resveratrol di-O-methyltransferase	trans-resveratrol di-O-methyltransferase-like	*DnROMTL*
110111843	6.03 ± 0.59	2.49 ± 0.02	5.34 ± 0.08	12.44 ± 4.09	79.64 ± 2.39		K16055//trehalose 6-phosphate synthase/phosphatase	alpha,alpha-trehalose-phosphate synthase [UDP-forming] 5-like	*DnTPS5L*
110103125	59.25 ± 1.82	0.5 ± 0	0.2 ± 0	0.04 ± 0.03	0 ± 0	C2C2-Dof	K16222//Dof zinc finger protein DOF5.5	cyclic dof factor 4-like	*DnC2C2-DOF4L*

110104932	739.32 ± 36.74	492.53 ± 19.49	1220.88 ± 106.9	1081.7 ± 216.41	1852.89 ± 220.88		K16292//KDEL-tailed cysteine endopeptidase	oryzain alpha chain-like	*DnOACL*
110096060	78.05 ± 9.96	57.22 ± 0.56	53.36 ± 4.53	208.14 ± 31.79	145 ± 14.73		K16302//metal transporter CNNM	metal transporter CNNM	*DnCNNM*
110107145	0.16 ± 0	95.55 ± 0.78	0 ± 0	0.15 ± 0.12	23.01 ± 7.96	HB	K16670//homeobox protein Meis2	homeobox protein knotted-1-like 2	*DnHB-KNO1L2*
110097716	7.97 ± 0.18	0 ± 0	0.87 ± 0.71	0 ± 0	0.26 ± 0.14		K16818//phospholipase A1	galactolipase DONGLE, chloroplastic-like	*DnDONGLE*
110111653	88.3 ± 19.09	14.38 ± 0.2	372.81 ± 70.54	26.04 ± 16.61	313.27 ± 25.52		K17471//sulfate transporter 3	sulfate transporter 3.1	*DnSULT3.1*
110097581	68.17 ± 3.4	490.52 ± 13.1	78.67 ± 5.14	112.96 ± 23.29	64.57 ± 5.89		K17497//phosphomannomutase	phosphomannomutase	*DnPPMM*
110106201	115.93 ± 4.89	181.94 ± 0.87	258.89 ± 38.94	1.21 ± 0.14	122.97 ± 16.08	GeBP	K17500//integrin-linked kinase-associated serine/threonine phosphatase 2C	WAT1-related protein At4g08290-like	*DnGeBP-WAT1*
110111194	0.03 ± 0.02	0 ± 0	2.82 ± 0.27	0 ± 0	0.09 ± 0.07		K17545//serine/threonine-protein kinase ULK4	Reverse transcriptase (RNA-dependent DNA polymerase)	*DnRVT2L2*
114578406	0 ± 0	0.06 ± 0	0 ± 0	0 ± 0	0 ± 0		K17545//serine/threonine-protein kinase ULK4	Reverse transcriptase (RNA-dependent DNA polymerase)	*DnRVT2L3*
110110400	2.55 ± 0.13	0 ± 0	0 ± 0	0.2 ± 0.07	0 ± 0	bHLH	K17693//DNA-binding protein inhibitor ID2	transcription factor ILI6-like	*DnbHLH-ILI6L*
110116746	73.73 ± 7.17	74.67 ± 1.67	5.59 ± 4.49	149.15 ± 114.23	7.31 ± 1.17	C2C2-GATA	K17895//GATA-binding protein 3	GATA transcription factor 4-like	*DnC2C2-GATA4L*
110110848	14.91 ± 0.09	0.45 ± 0.05	1.47 ± 0.05	1.18 ± 0.17	1.31 ± 0.36		K18081//myotubularin-related protein 1/2	Domain of unknown function (DUF3475)	*DnDUF3475*
110100507	127.21 ± 11.57	0.15 ± 0.02	0 ± 0	0 ± 0	0.02 ± 0.01		K18134//EGF domain-specific O-GlcNAc transferase	alpha 1,3 arabinosyltransferase XAT3 like	*DnXAT3L*
110096783	0 ± 0	0 ± 0	0 ± 0	0 ± 0	0.03 ± 0		K18195//rhamnogalacturonan endolyase	non-LTR retrovirus reverse transcriptase	*DnRT-NLTRL*
110103843	61.41 ± 2.65	5.75 ± 0.05	5.7 ± 0.08	3.13 ± 0.59	5.61 ± 1.02		K18204//D-2-hydroxyglutarate dehydrogenase	D-2-hydroxyglutarate dehydrogenase, mitochondrial	*DnHGDM*
110100598	45.7 ± 1.02	0.69 ± 0.1	0 ± 0	0.62 ± 0.51	0.57 ± 0.15		K18207//protein O-mannose beta-1,4-N-acetylglucosaminyltransferase	Protein of unknown function (DUF563)	*DnDUF563.1*
110103054	160.34 ± 0.6	11.63 ± 0.3	0.1 ± 0.08	1.32 ± 0.67	4.2 ± 0.12		K18207//protein O-mannose beta-1,4-N-acetylglucosaminyltransferase	Protein of unknown function (DUF563)	*DnDUF563.2*
110115756	2 ± 0.05	25.53 ± 0.01	0.07 ± 0.06	0.21 ± 0.17	0.02 ± 0.02	bHLH	K18485//myogenic factor 6	transcription factor bHLH94-like	*DnbHLH94L1*
110116721	0.22 ± 0.04	1.12 ± 0.04	0.14 ± 0.02	0.16 ± 0.03	0.02 ± 0.02	bHLH	K18485//myogenic factor 6	transcription factor bHLH94-like	*DnbHLH94L2*
110099096	299.18 ± 11.28	63.22 ± 2.29	1302.97 ± 107.8	174.57 ± 24.37	135.09 ± 14.4		K18696//glycerophosphodiester phosphodiesterase	glycerophosphodiester phosphodiesterase GDPD1, chloroplastic-like	*DnGDPD1C*
110106931	39.74 ± 3.65	4.32 ± 0.17	0.04 ± 0.03	0.78 ± 0.56	0.1 ± 0.02	C3H	K18753//butyrate response factor	zinc finger CCCH domain-containing protein 9-like	*DnC3H9L*
110095650	115.77 ± 10.14	2.74 ± 0.15	10.5 ± 2.64	4.97 ± 1.37	0.86 ± 0.66		K18801//glucuronoxylan 4-O-methyltransferase	protein IRREGULAR XYLEM 15-like	*DnIX15L*
110106009	15.38 ± 0.01	0.01 ± 0.01	0.07 ± 0	0.03 ± 0.02	0.33 ± 0.1	WRKY	K18835//WRKY transcription factor 2	probable WRKY transcription factor 72	*DnWRKY72*
110097793	59.17 ± 5.87	143.58 ± 1.08	3.86 ± 0.6	1215.32 ± 491.5	2.72 ± 1.67		K18875//enhanced disease susceptibility 1 protein	Abhydrolase	*DnABHL*
110104088	0.14 ± 0.03	1.34 ± 0.08	0.02 ± 0.01	0.01 ± 0.01	0.25 ± 0.08		K18886//gibberellin A4 carboxyl methyltransferase	gibberellic acid methyltransferase 2	*DnGAMT2*
110110171	28.29 ± 1.4	29.49 ± 0.82	231.09 ± 8.25	32.59 ± 11.94	28.83 ± 7.47		K19269//phosphoglycolate phosphatase	phosphoglycolate phosphatase 1B, chloroplastic-like	*DnPGP1BC*
110092809	19.05 ± 4.08	21.88 ± 0.94	3.31 ± 0.91	143.56 ± 8.2	0.54 ± 0.05		K19706//dihydroceramide fatty acyl 2-hydroxylase	dihydroceramide fatty acyl 2-hydroxylase FAH1	*DnFAH1*
110099878	0.28 ± 0.11	119.69 ± 2.69	8.82 ± 1.39	1.37 ± 0.67	2.05 ± 0.26		K19747//BAHD acyltransferase	BAHD acyltransferase DCR like	*DnBAHD-DCRL*
110093781	136.3 ± 6.04	0.51 ± 0.23	0.1 ± 0.08	0.09 ± 0.07	0.17 ± 0.07		K19891//glucan endo-1,3-beta-glucosidase 1/2/3	major pollen allergen Ole e 10-like	*DnMPAO10L*
110105726	201.44 ± 5.91	0.12 ± 0.03	11.24 ± 6.39	0.57 ± 0.33	23.89 ± 13.53		K19892//glucan endo-1,3-beta-glucosidase 4	glucan endo-1,3-beta-glucosidase-like	*DnGBGSL*
110100046	6.28 ± 1.26	2.41 ± 0.05	5.91 ± 2.11	304.18 ± 33.58	8.12 ± 0.56	C2C2-Dof	K20043//clathrin coat assembly protein AP180	dof zinc finger protein DOF1.2-like	*DnC2C2-DOF1.2L*
110109536	69.7 ± 0.55	32.11 ± 0.13	48.78 ± 7.05	10.02 ± 4.52	258.54 ± 70.44	C2C2-Dof	K20043//clathrin coat assembly protein AP180	dof zinc finger protein DOF3.1-like	*DnC2C2-DOF3.1L*
110110730	1.88 ± 0.16	17.71 ± 1.09	1.13 ± 0.33	1.35 ± 0.37	2.86 ± 0.45	C2C2-Dof	K20043//clathrin coat assembly protein AP180	dof zinc finger protein DOF2.1 like	*DnC2C2-DOF2.1L*
110108102	58.75 ± 13.93	146.44 ± 1.92	139.52 ± 13.37	37.78 ± 11.03	91.36 ± 12.56	C3H	K20283//golgin subfamily A member 4	zinc finger CCCH domain-containing protein 13	*DnC3H13*
110114706	7.54 ± 0.5	0.39 ± 0.16	0.03 ± 0	0.04 ± 0.03	0.06 ± 0.05		K20496//laurate 7-monooxygenase	cytochrome P450 703A2	*DnCYP703A2*
110101228	12.33 ± 0.78	0.06 ± 0.02	0.06 ± 0.02	0.3 ± 0.22	0.19 ± 0		K20506//tuliposide A-converting enzyme	carboxylesterase 2	*DnCAR2*

110113555	1453.28 ± 161.4	441.82 ± 4.69	47.51 ± 9.74	684.5 ± 131.26	221.54 ± 70.15		K20547//basic endochitinase B	chitinase-like protein 1	*DnCHI1*
110102102	3.66 ± 0.87	7.46 ± 0.94	84.61 ± 7.36	1.1 ± 0.88	15.72 ± 2		K20624//12-hydroxyjasmonoyl-L-amino acid 12-hydroxylase / fatty acid hydroxylase	Cytochrome P450 LIKE	*DnCYPL*
110116726	7.25 ± 0.4	0 ± 0	0.09 ± 0.02	0.11 ± 0.03	0 ± 0		K20802//(R)-mandelonitrile lyase	methylesterase 1-like	*DnME1L*
110101086	21.77 ± 1.26	4.92 ± 0.34	0.06 ± 0.04	0.42 ± 0.13	2.61 ± 1.02		K20802//(R)-mandelonitrile lyase	methylesterase 3-like	*DnME3L*
110093655	0.31 ± 0.02	2.39 ± 0.1	0.13 ± 0	0.24 ± 0.02	0 ± 0		K20855//beta-1,3-galactosyltransferase 1/2/3/4/5/7/8	Senescence regulator	*DnSRL1*
110107099	22.38 ± 0.08	147.74 ± 7.09	15.16 ± 1.38	11.92 ± 6.48	33.06 ± 6.09		K20855//beta-1,3-galactosyltransferase 1/2/3/4/5/7/8	Senescence regulator	*DnSRL2*
110115255	12.18 ± 0.2	17.04 ± 0.64	171.98 ± 5.68	5.91 ± 2.08	13.04 ± 0.92		K20884//riboflavin kinase / FMN hydrolase	haloacid dehalogenase-like hydrolase domain-containing protein At3g48420	*DnHD-HDP*
110095710	1.24 ± 0.2	1.28 ± 0.22	11.56 ± 0.74	0.71 ± 0.05	1.05 ± 0.28		K20884//riboflavin kinase / FMN hydrolase	putative phosphatase 3B Provisional (PRK11587)	*DnPRK11587*
114580039	0 ± 0	0.07 ± 0.05	0 ± 0	0 ± 0	1.45 ± 0.15		K20887//xyloglucan glycosyltransferase 4	gibberellin regulated protein 1 like	*DnGRP1L*
110115838	39.22 ± 2.81	0.87 ± 0.07	0 ± 0	0.63 ± 0.51	0.09 ± 0.07		K20888//xyloglucan galactosyltransferase MUR3	xyloglucan galactosyltransferase KATAMARI1 homolog	*DnKATAMARI1H*
110114924	5.58 ± 0.37	0 ± 0	0.02 ± 0.01	0.06 ± 0.03	0.24 ± 0.14		K20888//xyloglucan galactosyltransferase MUR3	xyloglucan specific galacturonosyltransferase 1 like	*DnXSGT1L*
110098291	98.47 ± 7.35	1.87 ± 0.14	0.21 ± 0.05	0.26 ± 0.11	0.77 ± 0.04		K20890//xylan alpha-glucuronosyltransferase	UDP-glucuronate:xylan alpha-glucuronosyltransferase 1-like	*DnYGXGT1L1*
110102381	49.83 ± 4.75	7.61 ± 0.17	0.51 ± 0.01	2.48 ± 1.56	2.12 ± 0.07		K20890//xylan alpha-glucuronosyltransferase	UDP-glucuronate:xylan alpha-glucuronosyltransferase 1-like	*DnYGXGT1L2*
110109313	6.61 ± 0.17	0.01 ± 0	0.19 ± 0.15	0.17 ± 0.09	0.57 ± 0.21		K20924//cellulose synthase-like protein	cellulose synthase like protein D3	*DnCLS-D3.1*
110116295	3.11 ± 0.17	0.07 ± 0.02	0.13 ± 0.02	0.29 ± 0.1	0.4 ± 0.11		K20924//cellulose synthase-like protein	cellulose synthase-like protein D3	*DnCLS-D3.2*
114578721	0.15 ± 0.02	0 ± 0	0 ± 0	0 ± 0	0 ± 0		K20924//cellulose synthase-like protein	zinc-binding in reverse transcriptase	*Dnzf-RVT*
110103047	7.93 ± 0.73	0.01 ± 0	0.01 ± 0.01	0.03 ± 0	0.01 ± 0		K21396//ATP-binding cassette, subfamily G (WHITE), eye pigment precursor transporter	ABC transporter G family member 42 like	*DnABCG42L*
110102612	4.65 ± 0.25	33 ± 0.73	0.36 ± 0.09	0.77 ± 0.05	0.56 ± 0.46	C2C2-GATA	K21630//GATA-type zinc finger protein 1	GATA transcription factor 9-like	*DnC2C2-GATA9L*
110094802	9.76 ± 0.73	13.98 ± 0.73	80.27 ± 5.32	0.52 ± 0.2	3.17 ± 2.16	C2C2-GATA	K21630//GATA-type zinc finger protein 1	putative GATA transcription factor 22	*DnC2C2-GATA22*
110112964	4.65 ± 0.42	0 ± 0	0 ± 0	0 ± 0	0.02 ± 0.02		K21737//acyl-lipid Delta6-acetylenase / acyl-lipid (9-3)-desaturase	acyl-lipid (9-3)-desaturase-like	*DnALDL2*
110112966	1.59 ± 0.11	0 ± 0	0 ± 0	0 ± 0	0 ± 0		K21737//acyl-lipid Delta6-acetylenase / acyl-lipid (9-3)-desaturase	acyl-lipid (9-3)-desaturase-like	*DnALDL1*
110110052	55.69 ± 12.62	74.38 ± 2.31	160.45 ± 37.49	347.12 ± 16.28	64.46 ± 22.9		K21989//calcium permeable stress-gated cation channel	CSC1-like protein ERD4	*DnERD4L*
110099306	14.84 ± 1.69	0.06 ± 0.05	0 ± 0	0 ± 0	0.07 ± 0.05	LOB	K21994//LOB domain-containing protein 18	LOB domain-containing protein 12-like	*DnLOB12L1*
110115007	0 ± 0	2.53 ± 0.16	0 ± 0	0 ± 0	0.09 ± 0.04	LOB	K21994//LOB domain-containing protein 18	LOB domain-containing protein 12-like	*DnLOB12L2*
110100295	0.15 ± 0.04	0.85 ± 0	0 ± 0	0 ± 0	0 ± 0	LOB	K21994//LOB domain-containing protein 18	LOB domain-containing protein 25-like	*DnLOB25L*
110113842	1.79 ± 0.22	255.5 ± 2.53	14 ± 1.98	10.08 ± 7.45	5.14 ± 0.45		K21995//unsaturated fatty acid epoxidase	cytochrome P450 77A4-like	*DnCYP77A4L*
110102081	43.97 ± 0.06	18.61 ± 0.03	92.13 ± 9.55	137.01 ± 63.12	574.8 ± 9.88		K22013//magnesium dechelatase	protein STAY-GREEN 1, chloroplastic	*DnSG1C*
110106770	273.07 ± 23.78	883.24 ± 55.02	2018.56 ± 104.9	109.18 ± 37.93	362 ± 76.14		K22048//mechanosensitive ion channel protein 4/5/6/7/8/9/10	mechanosensitive ion channel protein	*DnMICPL2*
110093565	7.42 ± 0.35	17.58 ± 0.94	117.99 ± 6.03	22.57 ± 4.15	21.07 ± 2.5		K22128//piezo-type mechanosensitive ion channel component 1/2	piezo-type mechanosensitive ion channel	*DnPTMIC*
110093695	129.75 ± 3.28	19.6 ± 0.31	236.63 ± 110.84	213.44 ± 153.64	274.02 ± 136.07		K22139//mitochondrial pyruvate carrier 2	early light-induced protein 1, chloroplastic-like	*DnELIP1C*
110093696	237.18 ± 0.8	2.58 ± 0	251.71 ± 199	333.87 ± 102.17	208.68 ± 96.93		K22139//mitochondrial pyruvate carrier 2	early light-induced protein 1, chloroplastic-like	*DnELIP1CL*
110110948	9.4 ± 0.72	0 ± 0	0 ± 0	0.18 ± 0.15	0.12 ± 0.09		K22395//cinnamyl-alcohol dehydrogenase	berberine bridge enzyme-like 23	*DnBBEL23*
110105716	4.66 ± 0.38	0.22 ± 0.02	0.02 ± 0.01	0.09 ± 0.02	0 ± 0	C2H2	K22410//PR domain zinc finger protein 16	zinc finger protein MAGPIE-like	*DnC2H2-MAGPIE*
110100929	1785.88 ± 149.7	317.02 ± 12.24	67.5 ± 5.23	125.99 ± 39.21	45.77 ± 2.59		K22439//tricetin 3',4',5'-O-trimethyltransferase	tricetin 3',4',5'-O-trimethyltransferase-like	*DnTOTMTL*

110112038	0.05 ± 0.04	1.59 ± 0.09	0.07 ± 0.01	0.08 ± 0.06	0.28 ± 0.07		K22522//cytokinin riboside 5'-monophosphate phosphoribohydrolase	cytokinin riboside 5'-monophosphate phosphoribohydrolase LOGL10	*DnLOGL10*
110104964	280.69 ± 11.03	7.41 ± 0.44	65.84 ± 47.41	24.04 ± 0.26	349.96 ± 50.15	C2H2	K22534//PR domain zinc finger protein 5	zinc finger protein 1-like	*DnC2H2-ZFP1L*
110095530	215.72 ± 28.2	1.82 ± 0.19	44.51 ± 22.9	25.3 ± 11.84	366.28 ± 20.78	C2H2	K22534//PR domain zinc finger protein 5	zinc finger protein ZAT10-like	*DnC2H2-ZAT10*
110095040	20.3 ± 2.18	10.09 ± 0.96	164.42 ± 11.06	4.16 ± 0.39	199.81 ± 48.05	C2H2	K22534//PR domain zinc finger protein 5	zinc finger protein ZAT11-like	*DnC2H2-ZAT11*
110107260	16.95 ± 0.5	2.16 ± 0.06	3.8 ± 0.9	0.57 ± 0.14	119.97 ± 3.08	C2H2	K22534//PR domain zinc finger protein 5	zinc finger protein ZAT5-like	*DnC2H2-ZAT5L*
110092267	13.52 ± 0.19	0.22 ± 0.04	0.26 ± 0.17	1.35 ± 0.68	1.48 ± 0.42		K22706//(R)-mandelonitrile beta-glucosyltransferase	7-deoxyloganetin glucosyltransferase-like	*DnDLGTL*
110112607	14.3 ± 0.67	0.55 ± 0.09	0.22 ± 0.12	0.35 ± 0.14	0.92 ± 0.4		K22733//magnesium transporter	microtubule-associated protein 70-5-like	*DnMAP70-5L*
110098548	2.53 ± 0.11	0.03 ± 0	0.02 ± 0.01	0 ± 0	0.36 ± 0.03		K22771//flavonol-3-O-L-rhamnoside-7-O-glucosyltransferase	UDP-glycosyltransferase 73B4-like	*DnUGT73B4L*
110099012	15.76 ± 0.37	0.2 ± 0.06	0.43 ± 0.35	0.56 ± 0.45	3.84 ± 0.4		K22771//flavonol-3-O-L-rhamnoside-7-O-glucosyltransferase	UDP-glycosyltransferase 90A2-like	*DnUGT90A2L*
110100597	69.18 ± 1.11	0.29 ± 0.01	0.08 ± 0.01	0.03 ± 0.02	0.71 ± 0.22		K22772//flavonol-3-O-glucoside L-rhamnosyltransferase	anthocyanidin 3-O-glucosyltransferase-like	*DnA3GTL1*
114581249	0 ± 0	0 ± 0	0 ± 0	0 ± 0	0.01 ± 0		K22804//structural maintenance of chromosomes protein 6	Domain of unknown function (DUF4283)	*DnDUF4283*
110100615	0.23 ± 0.02	0 ± 0	0.02 ± 0.01	0 ± 0	0 ± 0		K22804//structural maintenance of chromosomes protein 6	mechanosensitive ion channel protein	*DnMICPL1*
114579332	0 ± 0	0 ± 0	0.03 ± 0	0 ± 0	0 ± 0		K22804//structural maintenance of chromosomes protein 6	Reverse transcriptase _like	*DnRTL*
110100189	49.56 ± 1.38	54.46 ± 1.28	274.97 ± 21.25	38.8 ± 5.02	58.03 ± 17.28		K22857//EEF1A lysine methyltransferase 4	Rossmann-fold NAD(P)(+)-binding proteins	*DnNADBR*
110093152	162.56 ± 0.95	13.47 ± 0.15	52.45 ± 26.07	11.99 ± 2.16	618.4 ± 86.02	C3H	K23047//E3 ubiquitin-protein ligase UNKL	zinc finger CCCH domain-containing protein 33-like	*DnC3H33L*
110106932	1782.59 ± 80.15	645.03 ± 20.46	382.9 ± 8.85	517.47 ± 3.39	670.05 ± 143.08		K23050//phenylcoumaran benzylic ether reductase	isoflavone reductase-like protein	*DnIFRL*
110097096	4.72 ± 0.62	102.78 ± 2.99	23.66 ± 4.29	3.65 ± 2.84	2.25 ± 0.9		K23154//2-hydroxyflavanone C-glucosyltransferase	UDP-glucose:2-hydroxyflavanone C-glucosyltransferase-like	*DnUGHGTL*
110114587	121.08 ± 19.84	315.33 ± 10.52	179.11 ± 24.25	44.62 ± 6.81	132.26 ± 61.25	LOB		LOB domain-containing protein 38-like	*DnLOB38L*
110103295	0.14 ± 0.11	0.11 ± 0.09	0 ± 0	0.04 ± 0.03	316.64 ± 125.16	C2C2-YABBY		protein DROOPING LEAF-like	*DnC2C2-YDL*
110112256	0.08 ± 0	0 ± 0	0 ± 0	0 ± 0	0 ± 0	RWP-RK		protein RKD2-like	*DnRKD2L*
110095157	14.86 ± 0.49	0.6 ± 0.18	1.11 ± 0.16	2.49 ± 1.29	0.43 ± 0.22	NOZZLE		protein SPEAR1-like	*DnNOZZLE-SPEAR1L*
110111600	0 ± 0	1.17 ± 0.01	0.15 ± 0.01	0.01 ± 0.01	0 ± 0	SBP		squamosa promoter-binding-like protein 10	*DnSBP10L*
110112799	10.33 ± 0.71	2.07 ± 0.02	0.98 ± 0.19	0.95 ± 0.18	0.01 ± 0.01			S-type anion channel SLAH2-like	*DnSLAH2L*
110096742	0 ± 0	4.45 ± 0.48	0 ± 0	0 ± 0	0 ± 0	TCP		transcription factor TCP13-like	*DnTCP13L*
110113676	0.37 ± 0.12	1.88 ± 0.03	11.11 ± 0.05	0.14 ± 0.04	0.51 ± 0.19	TCP		transcription factor TCP24-like	*DnTCP24L*
110098834	56.71 ± 7.09	81.85 ± 0.52	58.52 ± 7.96	139.41 ± 23.58	34.13 ± 7.09	Trihelix		trihelix transcription factor GTL1-like	*DnTrihelix-GTL1*
110110101	0.16 ± 0.13	11.03 ± 0.21	1.3 ± 0.74	0.35 ± 0.16	0.84 ± 0.6	ABI3VP1		uncharacterized protein LOC110110101	*DnABI3VP1L2*
110114078	0.16 ± 0.03	8.7 ± 0.3	50.39 ± 3.75	5.99 ± 2.24	0.09 ± 0.04	TCP		uncharacterized protein LOC110114078	*DnTCPL1*
114580681	0.31 ± 0.25	15.2 ± 1.66	0.45 ± 0.36	0 ± 0	160.6 ± 6.2	C2H2		zinc finger protein 2	*DnC2H2-ZFP2*
110113041	20.83 ± 0.71	0 ± 0	0.04 ± 0.03	0 ± 0	0 ± 0	NOZZLE		protein SPEAR3 like	*DnNOZZLE-SPEAR3L*
110111251	0.03 ± 0.02	5.67 ± 0.09	0.02 ± 0.02	1.27 ± 0.48	0.24 ± 0.07	C2H2		zinc finger protein WIP2-like	*DnC2H2-WIP2L*
