# Supplementary figures and images for: Metabolic and Transcriptomic Profile Revealing the Differential Accumulating Mechanism in Different Parts of Dendrobium nobile
Source: Int J Mol Sci. 2024 May 14;25(10):5356. doi: 10.3390/ijms25105356 (PMC11121218; doi:10.3390/ijms25105356)

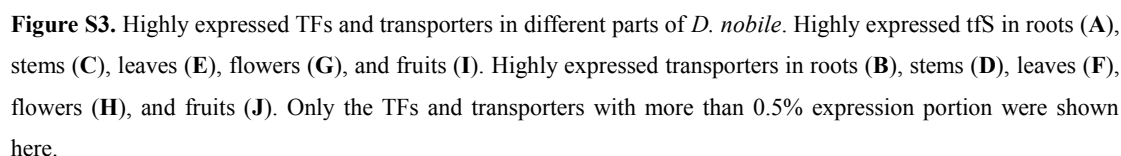

Supplement: Supplementary file 1 [file ijms-25-05356-s001.zip › Figure S3.pdf]
